# A practical way to synthesize chiral fluoro-containing polyhydro-2*H*-chromenes from monoterpenoids

**DOI:** 10.3762/bjoc.12.64

**Published:** 2016-04-06

**Authors:** Oksana S Mikhalchenko, Dina V Korchagina, Konstantin P Volcho, Nariman F Salakhutdinov

**Affiliations:** 1 1N. N. Vorozhtsov Novosibirsk Institute of Organic Chemistry, Siberian Branch, Russian Academy of Science, Lavrentiev ave., 9, 630090 Novosibirsk, Russian Federation; 2 Novosibirsk State University, Pirogova 2, 630090 Novosibirsk, Russian Federation

**Keywords:** chirality, fluorine, halo-Prins reaction, isopulegol, monoterpene

## Abstract

Conditions enabling the single-step preparative synthesis of chiral 4-fluoropolyhydro-2*H*-chromenes in good yields through a reaction between monoterpenoid alcohols with *para*-menthane skeleton and aldehydes were developed for the first time. The BF_3_·Et_2_O/H_2_O system is used both as a catalyst and as a fluorine source. The reaction can involve aliphatic aldehydes as well as aromatic aldehydes containing various acceptor and donor substituents. 4-Hydroxyhexahydro-2*H*-chromenes were demonstrated to be capable of converting to 4-fluorohexahydro-2*H*-chromenes under the developed conditions, the reaction occurs with inversion of configuration.

## Introduction

**Scheme 1:**
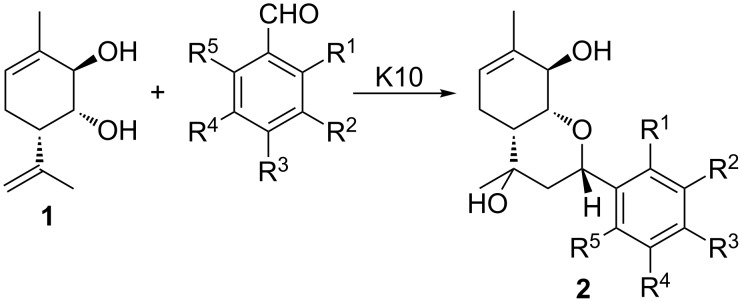
Reaction between monoterpenoid **1** and aromatic aldehydes in the presence of K10 montmorillonite clay.

Recently, we have found that a reaction between *para*-mentha-6,8-dien-2,3-diol (**1**) and aromatic aldehydes in the presence of K10 montmorillonite clay forms chiral heterocyclic compounds with the hexahydro-2*H*-chromene scaffold **2** (Scheme 1) [1–4]. Products of these reactions are of interest as many of them exhibit a significant analgesic activity in vivo [2–4].

In terms of structure–activity relationship studies of hexahydro-2*H*-chromenes and similar compounds it is important to replace the hydroxy group at the C(5) position by another functional group. Thus, the approaches for synthesis of thio- [5] and nitrogen [6] containing analogous with reasonable yields (50−80%) by using a third component (corresponding thiols or acetonitrile) were elaborated. Of particular interest is the introduction of a fluorine atom into the molecule of the biologically active compound. The introduction of a highly electronegative centre can lead to an increase in stability and changes in lipophilicity. Furthermore, it alters the patterns of reactivity of the C–F versus the C–H or the C–OH bond [7–9]. As a result it can have a significant impact on the biological activity of a compound.

**Scheme 2:**
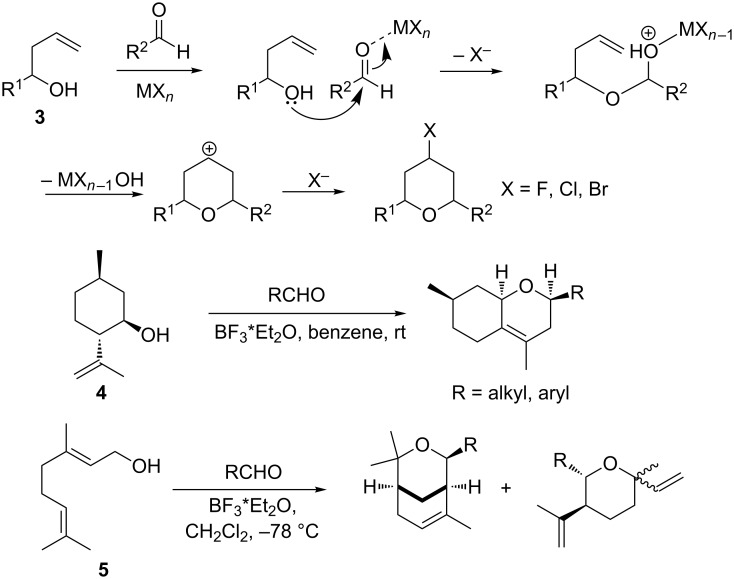
The Prins reaction between homoallylic alcohols and aldehydes.

To create halogenated tetrahydropyranyl rings the Prins reactions between homoallylic alcohols and aldehydes catalyzed by appropriate halogen-containing Lewis acids or ionic liquids are usually used (Scheme 2) [10–16]. However, only a few examples of reactions for introducing a fluorine atom via the halo-Prins cyclization have been reported to date [15–21]. In these reactions, BF_3_·Et_2_O acts both as a catalyst and as a fluorine source. At the same time, as a rule, reactions in the presence of BF_3_·Et_2_O involved relatively simple homoallylic alcohols, such as but-3-en-1-ol (**3**) and its analogues, as substrates (Scheme 2). If more complex substrates, such as isopulegol (**4**) or geraniol (**5**), were involved in transformations, the formation of non-fluorinated heterocyclic products was observed (Scheme 2) [22–23]. The aim of the present study was to find conditions for the synthesis of fluorinated chiral hexahydrochromenes based on monoterpenoids.

## Results and Discussion

The interaction of *para*-mentha-6,8-dien-2,3-diol (**1**) with 3,4,5-trimethoxybenzaldehyde (**6a**) in the presence of BF_3_·Et_2_O was chosen as a model reaction.

The study was started with the investigation of the effect of the reaction conditions and the reactant ratio on the yield of a fluorinated product (Table 1). The addition of an equimolar amount of BF_3_·Et_2_O to a mixture of monoterpenoid **1** and aldehyde **6a** at room temperature (Table 1, entry 1) resulted, after 1 h, in the formation of a reaction mixture containing compounds **2a** and **7a** as the major low molecular weight products and only minor amounts of target compound **8a**. It is important that the reaction is accompanied by a significant resinification. The addition of water to the initial reactants enabled the reduction of the amount of undesirable products **2a** and **7a** and had a minor effect on the amount of compound **8a**. This indicates a significant contribution of side processes that are likely related to the formation of high molecular weight products. Lowering the reaction temperature to 2 °C made it possible to reduce a contribution of resinification processes that led to an increase in the amount of product **8a** to 31%, but with incomplete conversion of monoterpenoid **1**. When the reaction time was increased to 8 h, the conversion increased, but was still not quantitative. The complete conversion of compound **1** was achieved by using a 1.5-fold excess of BF_3_·Et_2_O, and the use of a slight excess of aldehyde led to a marked increase in the amount of fluorinated product **8a** (61%). These conditions (Table 1, entry 6) were chosen as suitable for further research.

**Table 1:** The variation of reaction conditions.

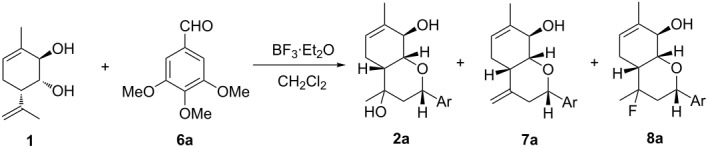							

Entry	Reagent ratio **1**:**6**:BF_3_·Et_2_O:H_2_O	Temp (°C)	Time (h)	Conv. **1** (%)	Yield (%)^a^		

					**2a**	**7a**^b^	**8a**

1	1:1:1:0	rt	1	100	12	13	5
2	1:1:1:7.4	rt	1	85	0	4	6
3	1:1:1:7.4	2	1	78	8	16	31
4	1:1:1:7.4	2	8	88	8	26	50
5	1:1:1.5:7.4	2	8	100	10	20	48
6	1:1.2:1.5:7.4	2	8	100	11	12	61

^a^The yields of products obtained from the GC–MS chromatograms using the standard (2,5-hexanediol) and the correction factors. ^b^Olefin **7a** is a mixture of double bond position isomers, and amount of **7a** shown in the table corresponds to the total yield of the olefin isomers. The structure of **7a** shown on the scheme is the major olefin isomer.

Preparative production under the chosen conditions provided 72% of **8a** by GC–MS and after separation by column chromatography the target fluorinated product **8a** was isolated in 69% yield; the yield of compound **2a** was 7% (Table 2). Thus, we found for the first time the conditions enabling production of chiral fluoro-containing heterocyclic compounds from a monoterpenoid using the halo-Prins cyclisation.

It should also be noted that preparative isolation of compounds of type **7a** is complicated by the presence of their double bond position isomers in the reaction mixtures. In this study, we did not seek to isolate the individual byproducts.

Compounds **2a** and **8a** produced by a reaction of diol **1** with aldehyde **6a** are formed as a mixture of diastereomers differing in the position of substituents at the C(5) carbon. The (*S*)-isomer predominates in the case of compound **2a**, while the (*R*)-isomer is the major one in the case of compound **8a** (Table 2). The ratio of the major and minor diastereomeric products of types **2** and **8** was determined from ^1^H NMR spectra by integration of the Ha-3 signal (Table 2). The methyl group at the C(5) carbon atom is axial in the (*S*)-isomers and equatorial in the (*R*)-isomers. Consequently, in the (*R*)-isomers the axial OH or F group causes a paramagnetic shift to a weak field of the Ha-3 signal due to the 1,3-diaxial interaction in comparison with the corresponding signal in the (*S*)-isomers.

**Table 2:** Yields of products **8a–m** and **2a–m** obtained in the reaction of diol **1** with aldehydes **6a–m**.

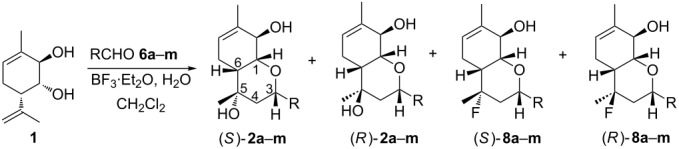				

**6**	R	Reaction time (h)	Yield^a^ (*R*:*S*)	

			**2**	**8**

**6a**	(3,4,5-MeO)C_6_H_2_	8	7% (2:3)	69% (4:1)
**6b**	C_6_H_5_	8	24% (1:3)	55% (7:1)
**6c**	4-MeOC_6_H_4_	8	24% (1:4)	34% (4:1)
**6d**	(3,4-MeO)C_6_H_3_	8	20% (1:5)	35% (3:1)
**6e**	(2,4,6-MeO)C_6_H_2_	8	35% (1:1)	42% (10:1)
**6f^b^**	(2,4,5-MeO)C_6_H_2_	8	8% (1:3)	20% (7:1)
**6g**	4-NO_2_C_6_H_4_	72	17% (1:1)	53% (12:1)
**6h**	4-FC_6_H_4_	72	17% (1:2)	47% (6:1)
**6i**	4-ClC_6_H_4_	72	17% (1:3)	58% (6:1)
**6j**	4-BrC_6_H_4_	72	13% (1:2)	60% (10:1)
**6k**	cyclo-C_6_H_11_	8	27% (1:3)	61% (3:1)
**6l**	CH_3_CH=CH	8	20% (1:2)	57% (3:1)
**6m**	4-OH-3-MeO-C_6_H_3_	8	35% (1:3)	60% (3:1)

^a^Reaction conditions: diol **1** (2.4 mmol), aldehyde (2.9 mmol), BF_3_·Et_2_O (3.6 mmol) and H_2_O (17.8 mmol). ^b^Product **9f** with epoxychromene framework was also isolated in 14% yield.

The next stage of our research was to study the effect of substituents at the aldehyde aromatic ring on the yield and the ratio of the reaction products. Thus, a reaction of monoterpenoid **1** with benzaldehyde (**6b**) under previously chosen conditions provided fluorinated product **8b** in 55% yield. In addition, compound **2b** was isolated from the reaction mixture in 24% yield (Table 2).

Introduction of one or two methoxy groups into the aldehyde aromatic ring reduced the yield of fluorinated products **8c** and **8d** to ca. 35%, without affecting the yields of compounds **2**.

To find out how much this reaction is sensitive to steric hindrances, we studied a reaction of monoterpenoid **1** with 2,4,6-trimethoxybenzaldehyde (**6e**), in which both *ortho*-positions are occupied. Previously, using the aldehyde as a reactant in a reaction with diol **1** catalyzed by K10 montmorillonite clay led to a sharp decrease in the yield of product **2e** [2]. In our case, the yields of products containing a fluorine atom (**8e**) and a hydroxy group (**2e**) were 42% and 35%, respectively (Table 2).

**Scheme 3:**
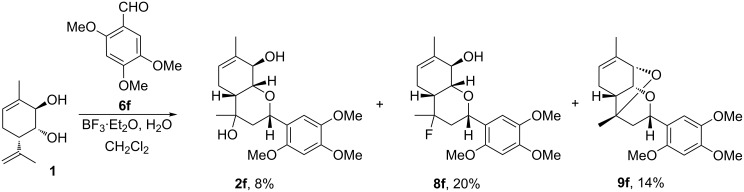
Reaction of compound **1** with aldehyde **6f**.

Interestingly, in the case of 2,4,5-trimethoxybenzaldehyde (**6f**), containing one of the three methoxy groups at the *ortho*-position, the overall yield of reaction products unexpectedly decreased, and the yield of fluorinated product **8f** was only 20%. In addition to the expected products **2f** and **8f**, tricyclic compound **9f** with an epoxychromene scaffold was isolated from the reaction mixture (Scheme 3), whose formation was previously observed only when using K10 clay [24].

The use of 4-nitrobenzaldehyde (**6g**) required the increase of the reaction time to 72 h for achieving complete conversion of monoterpenoid **1**, which is obviously due to the electron-withdrawing effect of the nitro group; the yield of compound **8g** was 53%. Reactions with 4-halogen-substituted aldehydes **6h–j** proceeded similarly and provided target products **8h–j** with yields of 47−60%.

Aliphatic aldehydes **6k** and **6l** were comparable in terms of reactivity with benzaldehyde (**6b**) and its methoxyderivatives; complete conversion was achieved after 8 h. It should be noted that the presence of a double bond in aldehyde **6l** had no significant effect on the reaction; yields of fluorinated products **8k** and **8l** were about 60%.

A reaction of diol **1** with 3-methoxy-4-hydroxybenzaldehyde (**6m**) in the presence of BF_3_·Et_2_O and water for 8 h also produces fluoro-containing hexahydrochromenes **8m** in 60% yield despite the presence of a phenolic group in the aldehyde aromatic ring.

It is known that the presence of water in the reaction medium containing BF_3_·Et_2_O can result in the formation of BF_3_·H_2_O, since the interaction of BF_3_ with H_2_O is relatively stronger than that with Et_2_O [25–26]. BF_3_·H_2_O, in turn, is a strong Brønsted acid and may be presented as H^+^(BF_3_·OH)^−^ [27–28]. Based on these data, it may be supposed that in the case of a 5-fold excess of water relative to BF_3_·Et_2_O, the reaction medium may contain both BF_3_·H_2_O and products of the partial hydrolysis of BF_3_·Et_2_O that may act both as catalysts and as fluorine sources.

**Scheme 4:**
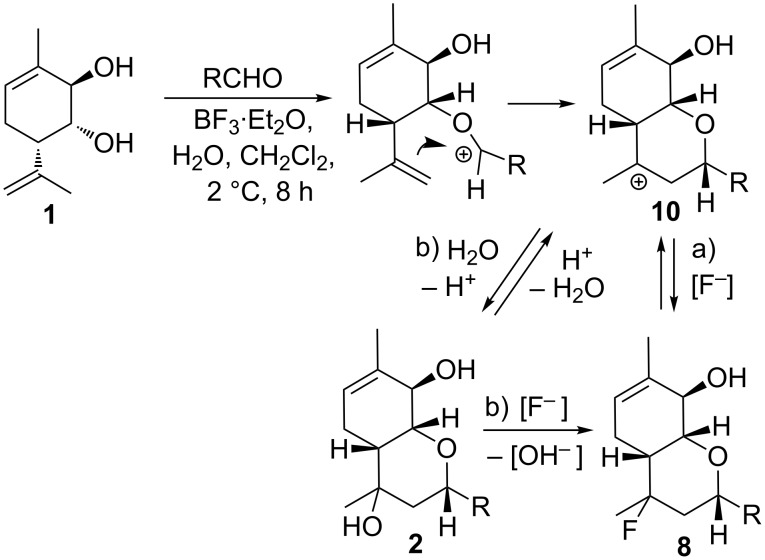
A possible mechanism of the compound **2** and **8** formation.

Presumably, the reaction starts with the Prins cyclisation resulting in the formation of cation **10**. There are then several mechanistic pathways, some or all of them may be in operation: a) cation **10** may undergo stereoselective trap by [F^−^] to form fluoride epimers **8**; b) cation **10** may undergo stereoselective trap by H_2_O to form alcohol epimers **2**; these may then undergo a stereospecific S_N_2 reaction to form fluoride epimers **8**; c) the fluorination and/or hydroxylation of cation **10**, forming fluoride epimers **8** and alcohol epimers **2**, respectively, may be reversible. This has several effects: firstly, reversible hydroxylation means that alcohol epimers **2** may convert to fluoride epimers **8** via cation **10** (pathway (a)); secondly, reversible fluorination and/or hydroxylation means that the diastereoselectivity of formation and/or **2** may be governed by product stability and not inherent stereoselectivity of the trapping of cation **10** (Scheme 4).

Re-subjection of alcohol **2b** epimers ((*R*):(*S*) = 1:4) to the reaction conditions gave fluoride epimers **8b** ((*R*):(*S*) = 8:1), albeit along with a number of side products and resinification processes. This result establishes that the alcohol epimers **2b** can convert to fluoride epimers **8b** under the reaction conditions but the apparent inversion of epimeric ratio (**2b** (*R*):(*S*) = 1:4, **8b** (*R*):(*S*) = 8:1) is not necessarily a specific evidence for the operation of pathway (b): the alcohol epimers **2b** may rect via different pathways and the amplification of the epimeric ratio would be consistent with a stereoselective aspect to the conversion. Further experimentation is therefore needed to elucidate the precise mechanistic details of this reaction.

Isopulegol (**4**), like diol **1**, has a hydroxy group and an isopropenyl group at the 1- and 2-positions; these two groups are *trans*-located in compound **4** (while *cis*-located in monoterpenoid **1**).

**Scheme 5:**
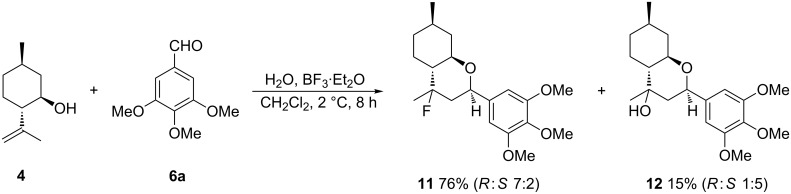
Reaction of isopulegol (**4**) with aldehyde **6a** in the presence BF_3_·Et_2_O.

When a reaction of isopulegol (**4**) with 3,4,5-trimethoxybenzaldehyde (**6a**) was conducted under the conditions previously optimised for diol **1**, fluorinated product **11** with the chromene scaffold and its analogue **12** with a hydroxy group were obtained in 76% and 15% yield, respectively (Scheme 5).

## Conclusion

We developed for the first time conditions enabling the single-step preparative production of chiral 4-fluoropolyhydro-2*H*-chromenes via a reaction between monoterpenoid alcohols with the *para*-menthane skeleton and aldehydes. The BF_3_·Et_2_O/H_2_O system is used both as a catalyst and as a fluorine source. The reaction can involve saturated and unsaturated aliphatic aldehydes as well as aromatic aldehydes containing various acceptor and donor substituents, including a phenolic hydroxy group. The yield of target fluorinated products usually ranges from 53% to 76%; byproducts having a hydroxy group instead of the fluorine atom are formed in smaller quantities. The possibility of a transformation of 4-hydroxyhexahydro-2*H*-chromenes to 4-fluorohexahydro-2*H*-chromenes with inversion of the configuration was demonstrated.

## Supporting Information

File 1 Detailed experimental procedures, compound characterization data, and copies of NMR spectra.
