# Supplementary material for: A practical way to synthesize chiral fluoro-containing polyhydro-2H-chromenes from monoterpenoids
Source: Beilstein J Org Chem. 2016 Apr 6;12:648–53. doi: 10.3762/bjoc.12.64 (PMC4902074; doi:10.3762/bjoc.12.64)
Supplement: File 1 — Detailed experimental procedures, compound characterization data, and copies of NMR spectra. [file Beilstein_J_Org_Chem-12-648-s001.pdf]

**Supporting Information**
**for**

# A practical way to synthesize chiral fluoro-containing polyhydro-2*H*-chromenes from monoterpenoids

Oksana S. Mikhalchenko[1], Dina V. Korchagina[1], Konstantin P. Volcho*[1,2], and Nariman F. Salakhutdinov[1,2]

Address: [1]N. N. Vorozhtsov Novosibirsk Institute of Organic Chemistry, Siberian Branch, Russian Academy of Science, Lavrentiev ave., 9, 630090 Novosibirsk, Russian Federation and [2]Novosibirsk State University, Pirogova 2, 630090 Novosibirsk, Russian Federation

Email: Konstantin P. Volcho* - volcho@nioch.nsc.ru

*Corresponding author

## Detailed experimental procedures, compound characterization data, and copies of NMR spectra

### Table of contents

Experimental part – General procedure and instrumentation ....................s2
Reaction of diol **1** with 3,4,5-trimethoxybenzaldehyde (**6a**) ....................s3
Reaction of diol **1** with benzaldehyde (**6b**) ....................s3
Reaction of diol **1** with 4-methoxybenzaldehyde (**6c**) ....................s4
Reaction of diol **1** with 3,4-dimethoxybenzaldehyde (**6d**) ....................s5
Reaction of diol **1** with 2,4,6-trimethoxybenzaldehyde (**6e**) ....................s6
Reaction of diol **1** with 2,4,5-trimethoxybenzaldehyde (**6f**) ....................s7
Reaction of diol **1** with 4-nitrobenzaldehyde (**6g**) ....................s7
Reaction of diol **1** with 4-fluorobenzaldehyde (**6h**) ....................s9
Reaction of diol **1** with 4-chlorobenzaldehyde (**6i**) ....................s10
Reaction of diol **1** with 4-bromobenzaldehyde (**6j**) ....................s11
Reaction of diol **1** with cyclohexanecarbaldehyde (**6k**) ....................s13
Reaction of diol **1** with (*E*)-but-2-enal (**6l**) ....................s14
Reaction of diol **1** with 4-hydroxy-3-methoxybenzaldehyde (**6m**) ....................s15
Reaction of isopulegol **4** with 3,4,5-trimethoxybenzaldehyde (**6a**) ....................s16
Spectra ....................s18
References ....................s43

**Experimental part**

*General methods*: All the chemicals were of commercial reagent grade. $CH_2Cl_2$ was passed through calcined $Al_2O_3$. (1*R*,2*R*,6*S*)-3-Methyl-6-(prop-1-en-2-yl)cyclohex-3-ene-1,2-diol 1 ($[\alpha]_D^{31}$= −49.1 (*c* = 2.6, $CHCl_3$)) was synthesized according to [1] from (−)-verbenone (*Aldrich*), the content of the main substance was not less than 98.0%. Column chromatography (CC): silica gel ($SiO_2$; 60–200 μ; Macherey-Nagel). GC/MS (purity control and products analysis): Agilent 7890A with a quadrupole mass spectrometer Agilent 5975C as a detector, HP-5MS quartz column, 30000x0.25 mm, He (1 atm) as carrier gas. Optical rotation: *polAAr 3005* spectrometer, $CHCl_3$ soln. HR-MS: DFS-Thermo-Scientific spectrometer in a full scan mode (15–500 *m*/*z*, 70 eV electron-impact ionization, direct sample introduction). $^1H$ and $^{13}C$ NMR: Bruker DRX-500 apparatus at 500.13 MHz ($^1H$) and 125.76 MHz ($^{13}C$) in $CDCl_3$ or $CDCl_3/CD_3OD$ (10:1 v/v); chemical shifts δ in ppm rel. to residual $CHCl_3$ (δ(H) 7.24, δ(C) 76.90 ppm), *J* in Hz; structure determinations by analyzing the $^1H$ NMR spectra, including $^1H$–$^1H$ double resonance spectra and $^1H$–$^1H$ 2D homonuclear correlation, J-modulated $^{13}C$ NMR spectra (JMOD), and $^{13}C$–$^1H$ 2D heteronuclear correlation with one-bond and long-range spin–spin coupling constants (C–H COSY, $^1J$(C,H) = 160 Hz, COLOC, $^{2,3}J$(C,H) = 10 Hz).

*General procedure:* Monoterpenoid (2.4 mmol) and aldehyde (2.9 mmol) were dissolved in $CH_2Cl_2$ (5 ml) and cooled to 2 °C. Then water (17.8 mmol) was added to the $BF_3 \cdot Et_2O$ (3.6 mmol) solution in $CH_2Cl_2$ (5 ml) under vigorous stirring. Resulting cloudy solution of $BF_3 \cdot Et_2O$ was added dropwise to the mixture of aldehyde and monoterpenoid, and then the reaction mixture was stirred for 8 or 72 hours at 2 °C. Then 10 % $NaHCO_3$ solution was added, the layers were separated and the aqueous phase was extracted with $CH_2Cl_2$ (2 x 15 ml). The combined organic layers were dried over $Na_2SO_4$, filtered and concentrated. Reaction mixture was separated on a $SiO_2$ column (hexane/EtOAc 100:0–0:100 as eluent).

**Reaction of 1 with 3,4,5-trimethoxybenzaldehyde (6a):** The reaction of diol **1** and aldehyde **6a** for 8 hours at 2 °C gave products **2a** (*R*:*S* = 2:3, 7%), **8a** (*R*:*S* = 4:1, 69%). Spectral characteristics of compounds **2a** have coincided with those described in the work [2].

**(2*S*,4*R*,4a*R*,8*R*,8a*R*)-4-fluoro-4,7-dimethyl-2-(3,4,5-trimethoxyphenyl) -3,4,4a,5,8,8a-hexahydro-2*H*-chromen-8-ol ((*R*)-8a):** Yellow viscous oil. $^1$H NMR (CDCl$_3$): δ = 1.36 (d, $^3J$(17, F) = 22.4 Hz, 3 H, 17-H); 1.78 (ddd, $^3J$(4a,F) = 39.4 Hz, *J*(4a,4e) = 14.7 Hz, *J*(4a,3a) = 11.7 Hz, 1H, 4-H$_a$); 1.81 (m, all *J* ≤ 2.5 Hz, 3H, 18-H); 1.90 (dddd, *J*(4e,4a) = 14.7 Hz, $^3J$(4e,F) = 11.3 Hz, *J*(4e, 3a) = 3.0 Hz, *J*(4e,6a) = 1.1 Hz, 1H, 4-H$_e$); 1.93-2.03 (m, H$_a$-C(6), 2 H, 7-H); 3.78 (s, 3 H, 20-MeO); 3.82 (s, 6 H, 19-MeO, 21-MeO); 3.94 (br.s, 1 H, 10-H$_e$); 4.14 (m, all *J* ≤ 3.0 Hz, 1 H, 1-H$_e$); 4.67 (dd, *J*(3a,4a) = 11.7 Hz, *J*(3a,4e) = 3.0 Hz, 1 H, 3-H$_a$); 5.55-5.59 (m, 1 H, 8-H); 6.51 (s, 2 H, 12-H, 16-H). $^{13}$C NMR (CDCl$_3$) δ = 20.75 (C-18); 24.04 (d, $^3J$(C-F) = 9.7 Hz, C-7); 24.77 (d, $^2J$(C-F) = 24.3 Hz, C-17); 36.08 (d, $^2J$(C-F) = 20.8 Hz, C-6); 40.13 (d, $^2J$(C-F) = 21.7 Hz, C-4); 56.04 (C-19, C-21); 60.61 (C-20); 70.08 (C-10); 75.51 (C-1); 75.96 (C-3); 95.15 (d, $^1J$(C-F) = 168.5 Hz, C-5); 103.09 (C-12, C-16); 123.12 (C-8); 132.16 (C-9); 137.41, 137.46 (C-11, C-14); 153.17 (C-13, C-15). HR-MS: 366.1833 ($M^+$ $(C_{20}H_{27}O_5F)^+$; calc. 366.1837).

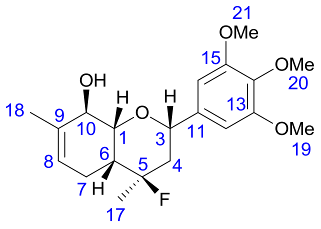

**(2*S*,4*S*,4a*R*,8*R*,8a*R*)-4-fluoro-4,7-dimethyl-2-(3,4,5-trimethoxyphenyl) -3,4,4a,5,8,8a-hexahydro-2*H*-chromen-8-ol ((*S*)-8a):** Yellow viscous oil. $^1$H NMR (CDCl$_3$): δ = 1.61 (d, $^3J$(17, F) = 23.5 Hz, 3 H, 17-H); 1.78 (m, all *J* ≤ 2.5 Hz, 3 H, 18-H); 1.79-1.83 (m, 1 H, 4-H$_e$); 2.04 (br.dd, *J*(6a,7a) = 10.7 Hz, *J*(6a,7e) = 6.6 Hz, 1 H, 6-H$_a$); 2.07-2.21 (m, 3 H, H$_a$-4, 7-2H); 3.68 (m, all *J* ≤ 3.0 Hz, 1 H, 1-H$_e$); 3.75 (s, 3 H, 20-MeO); 3.80 (s, 6 H, 19-MeO, 21-MeO); 3.87 (br.s, 1 H, 10-H$_e$); 4.27 (dd, *J*(3a,4a) = 12.1 Hz, *J*(3a,4e) = 2.6 Hz, 1 H, 3-H$_a$); 5.59-5.63 (m, 1H, 8-H); 6.49 (s, 2 H, 12-H, 16-H). $^{13}$C NMR (CDCl$_3$) δ = 20.53 (C-18); 22.39 (d, $^3J$(C-F) = 6.2 Hz, C-7); 24.68 (d, $^2J$(C-F) = 24.8 Hz, C-17); 37.65 (d, $^2J$(C-F) = 18.7 Hz, C-6); 40.41 (d, $^2J$(C-F) = 20.6 Hz, C-4); 55.97 (C-19, C-21); 60.55 (C-20); 69.74 (C-10); 77.73 (d, $^3J$(C-F) = 11.5 Hz, C-3); 78.05 (d, $^3J$(C-F) = 7.5 Hz, C-1); 95.09 (d, $^1J$(C-F) = 176.1 Hz, C-5); 103.24 (C-12, C-16); 123.97 (C-8); 131.24 (C-9); 137.34, 137.56 (C-11, C-14); 153.06 (C-13, C-15).

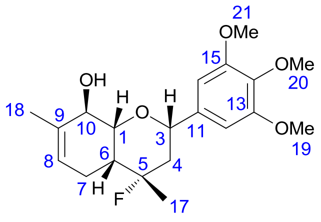

**Reaction of 1 with benzaldehyde (6b):** The reaction of diol **1** and aldehyde **6b** for 8 hours at 2 °C gave products **2b** (*R*:*S* = 1:3, 24%), **8b** (individual *R*-isomer, 10%) and **8b** (*R*:*S* = 5.5:1, 45%). Spectral characteristics of compounds **2b** have coincided with those described in the work [2].

**(2*S*,4*R*,4a*R*,8*R*,8a*R*)-4-fluoro-4,7-dimethyl-2-phenyl-3,4,4a,5,8,8a-hexahydro-2*H*-chromen-8-ol ((*R*)-8b):** Yellow oil. $^{1}$H NMR ($CDCl_3$): δ = 1.36 (d, $^{3}J$(17, F) = 22.3 Hz, 3 H, 17-H); 1.80 (ddd, $^{3}J$(4a, F) = 40.1 Hz, *J*(4a,4e) = 14.7 Hz, *J*(4a, 3a) = 11.6 Hz, 1 H, 4-H$_a$); 1.84 (m, all *J* ≤ 3.0 Hz, 3 H, H-18); 1.92 (dddd, *J*(4e, 4a) = 14.7 Hz, $^{3}J$(4e, F) = 10.6 Hz, *J*(4e,3a) = 3.0 Hz, *J*(4e,6a) = 1.2 Hz, 1 H, 4-H$_e$); 1.94-2.07 (m, 3 H, 6-H$_a$, 7-2H); 3.95 (br.s, 1 H, 10-H$_e$); 4.16 (m, all *J* ≤ 3.0 Hz, 1 H, 1-H$_e$); 4.76 (dd, *J*(3a,4a) = 11.6 Hz, *J*(3a,4e) = 3.0 Hz, 1 H, 3-H$_a$); 5.57-5.61 (m, 1 H, 8-H); 7.22-7.33 (m, 5 H, Ph-H). $^{13}$C NMR ($CDCl_3$) δ = 20.79 (C-18); 24.06 (d, $^{3}J$(C-F) = 9.3 Hz, C-7); 24.76 (d, $^{2}J$(C-F) = 24.3 Hz, C-17); 36.05 (d, $^{2}J$(C-F) = 20.8 Hz, C-6); 40.38 (d, $^{2}J$(C-F) = 21.7 Hz, C-4); 70.08 (C(10)); 75.27 (d, $^{3}J$(C-F) = 1.3 Hz, C-1); 75.66 (C-3); 95.09 (d, $^{1}J$(C-F)=169.0 Hz, C-5); 123.14 (C-8); 125.67 (C-12, C-16); 127.43 (C-14); 128.27 (C-13, C-15); 132.19 (C-9); 141.95 (C-11). HR-MS: 276.1523 ($M^+$ $(C_{17}H_{21}O_2F)^+$; calc. 276.1520).

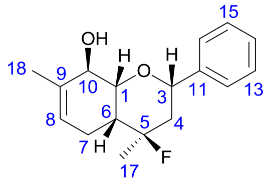


**(2*S*,4*S*,4a*R*,8*R*,8a*R*)-4-fluoro-4,7-dimethyl-2-phenyl-3,4,4a,5,8,8a-hexahydro-2*H*-chromen-8-ol ((*S*)-8b):** Yellow oil. $^{1}$H NMR ($CDCl_3$): δ = 1.65 (dd, $^{3}J$(17, F) = 23.5 Hz, *J*(17, 4a) = 0.8 Hz, 3 H, 17-H); 1.82 (m, all *J* ≤ 2.5 Hz, 3H,18-H); 1.86 (br.dd, *J*(4e,4a) = 13.3 Hz, *J*(4e,3a) = 2.7 Hz, 1 H, 4-H$_e$); 2.07 (br.dd, *J*(6a,7a) = 10.7 Hz, *J*(6a,7e) = 6.5 Hz, 1 H, 6-H$_a$); 2.15 (dddq, $^{3}J$(4a, F) = 14.1 Hz, *J*(4a,4e) = 13.3 Hz, *J*(4a,3a) = 12.1 Hz, *J*(4a, 17) = 0.8 Hz, 1 H, 4-H$_a$); 2.16-2.29 (m, 2 H, 7-H); 3.73 (ddd, *J*(1e,10e) = 2.3 Hz, *J*(1e,6a) = $^{4}J$(1e,F) = 2.1 Hz, 1 H, 1-H$_e$); 3.93 (m, *all J* ≤ 3.0 Hz, 1 H, 10-H$_e$); 4.38 (dd, *J*(3a,4a) = 12.1 Hz, *J*(3a,4e) = 2.7 Hz, 1 H, 3-H$_a$); 5.64-5.67 (m, 1 H, 8-H); 7.24-7.34 (m, 5 H, Ph-H). $^{13}$C NMR ($CDCl_3$) δ = 20.68 (C-18); 22.54 (d, $^{3}J$(C-F) = 6.3 Hz, C-7); 24.80 (d, $^{2}J$(C-F) = 26.0 Hz, C-17); 37.74 (d, $^{2}J$(C-F) = 18.6 Hz, C-6); 40.90 (d, $^{2}J$(C-F) = 20.8 Hz, C-4); 70.20 (d, $^{4}J$(C-F) = 2.3 Hz, C-10); 77.6 (d, $^{3}J$(C-F) = 11.6 Hz, C-3); 77.8 (d, $^{3}J$(C-F) = 7.6 Hz, C-1); 95.14 (d, $^{1}J$(C-F)=176.6 Hz, C-5); 124.41 (C-8); 125.79 (C-12, C-16); 127.73 (C-14); 128.36 (C-13, C-15); 131.29 (C-9); 141.26 (C-11).

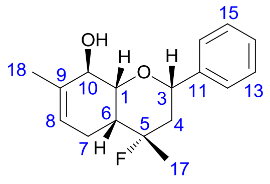


**Reaction of 1 with 4-methoxybenzaldehyde (6c):** The reaction of diol **1** and aldehyde **6c** for 8 hours at 2 °C gave products **2c** (*R*:*S* = 1:4, 24%), **8c** (*R*:*S* = 4:1, 34%). Spectral characteristics of compounds **2c** have coincided with those described in the work [3].

**(2*S*,4*R*,4a*R*,8*R*,8a*R*)-4-fluoro-2-(4-methoxyphenyl)-4,7-dimethyl-3,4,4a,5,8,8a-hexahydro-2*H*-chromen-8-ol ((*R*)-8c):** Yellow viscous oil. $^{1}$H NMR ($CDCl_3$): δ = 1.35 (d, $^{3}J$(17, F) = 22.4 Hz, 3 H, 17-H); 1.80 (ddd, $^{3}J$(4a, F) = 39.4 Hz, *J*(4a,4e) = 14.7 Hz, *J*(4a, 3a) = 11.5 Hz, 1 H, 4-H$_a$); 1.82 (m,

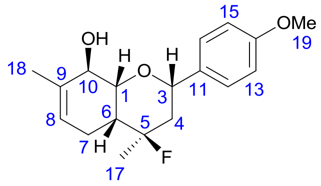


all $J \leq 3.0$ Hz, 3 H, 18-H); 1.84-1.91 (m, 1H, 4-$H_e$); 1.91-2.06 (m, 3H, 6-$H_a$, 7-2H); 3.76 (s, 3 H, 19-MeO); 3.93 (br.s, 1H, 10-$H_e$); 4.14 (m, all $J \leq$ 3.0 Hz, 1 H, 1-$H_e$); 4.70 (dd, $J$(3a,4a) = 11.5 Hz, $J$(3a,4e) = 3.2 Hz, 1 H, 3-$H_a$); 5.55-5.59 (m, 1 H, 8-H); 6.84 (d, $J$(13,12) = $J$(15,16) = 8.8 Hz, 2 H, 13-H, 15-H); 7.23 (d, J(12,13) = J(16,15) = 8.8 Hz, 2 H, 12-H, 16-H). $^{13}$C NMR ($CDCl_3$) δ = 20.79 (C-18); 24.09 (d, $^3J$(C-F) = 9.3 Hz, C-7); 24.79 (d, $^2J$(C-F) = 24.8 Hz, C-17); 36.08 (d, $^2J$(C-F) = 20.8 Hz, C-6); 40.23 (d, $^2J$(C-F) = 21.2 Hz, C-4); 55.17 (C-19); 70.16 (C-10); 75.34 (d, $^3J$(C-F) = 1.3 Hz, C-1); 75.36 (C-3); 95.15 (d, $^1J$(C-F)=168.5 Hz, C-5); 113.74 (C-13, C-15); 123.20 (C-8); 127.08 (C-12, C-16); 132.21 (C-9); 134.10 (C-11); 159.01 (C-14). HR-MS: 306.1623 ($M^+$ $(C_{17}H_{23}O_3F)^+$; calc. 306.1626).

**(2*S*,4*S*,4a*R*,8*R*,8a*R*)-4-fluoro-2-(4-methoxyphenyl)-4,7-dimethyl-3,4,4a,5,8,8a-hexahydro-2*H*-chromen-8-ol ((*S*)-8c):** Yellow viscous oil. $^1$H NMR ($CDCl_3$): δ = 1.63 (dd, $^3J$(17, F) = 23.5 Hz,

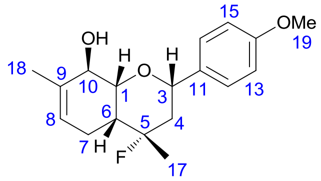


$J$(17,4a) = 0.7 Hz, 3 H, 17-H); 1.80 (m, all $J \leq 2.5$ Hz, 3H, 18-H); 1.82 (br.dd, $J$(4e,4a) = 13.3 Hz, $J$(4e, 3a) = 2.7 Hz, 1 H, 4-$H_e$); 2.05 (br.dd, $J$(6a,7a) = 10.8 Hz, $J$(6a,7e) = 6.5 Hz, 1 H, 6-$H_a$); 2.16 (ddd, $^3J$(4a, F) = 14.1 Hz, $J$(4a,4e) = 13.3 Hz, $J$(4a, 3a) = 12.0 Hz, 1 H, 4-$H_a$); 2.17-2.27 (m, 2 H, 7-H); 3.71 ($J$(1e,10e) = 2.4 Hz, $J$(1e,6a) = $^4J$(1e,F) = 2.1 Hz, 1 H, 1-$H_e$); 3.76 (s, 3 H, 19-MeO); 3.90 (m, all $J \leq 3.0$ Hz, 1 H, 10-$H_e$); 4.32 (dd, $J$(3a,4a) = 12.0 Hz, $J$(3a,4e) = 2.7 Hz, 1 H, 3-$H_a$); 5.62-5.66 (m, 1 H, 8-H); 6.84 (d, $J$(13,12) = $J$(15,16) = 8.8 Hz, 2 H, 13-H, 15-H); 7.23 (d, J(12,13) = J(16,15) = 8.8 Hz, 2 H, 12-H, 16-H). $^{13}$C NMR ($CDCl_3$) δ = 20.65 (C-18); 22.50 (d, $^3J$(C-F) = 6.2 Hz, C-7); 24.77 (d, $^2J$(C-F) = 25.7 Hz, C-17); 37.71 (d, $^2J$(C-F) = 19.0 Hz, C-6); 40.70 (d, $^2J$(C-F) = 20.4 Hz, C-4); 55.15 (C-19); 70.12 (d, $^4J$(C-F) = 2.2 Hz, C-10); 77.26 (d, $^3J$(C-F) = 11.5 Hz, C-3); 77.83 (d, $^3J$(C-F) = 7.5 Hz, C-1); 95.18 (d, $^1J$(C-F)=176.0 Hz, C-5); 113.74 (C-13, C-15); 124.35 (C-8); 127.16 (C-12, C-16); 131.25 (C-9); 133.41 (C-11); 159.15 (C-14).

**Reaction of 1 with 3,4-dimethoxybenzaldehyde (6d):** The reaction of diol **1** and aldehyde **6d** for 8 hours at 2 °C gave products **2d** (*R*:*S* = 1:5, 20%), **8d** (*R*:*S* = 3:1, 35%). Spectral characteristics of compounds **2d** have coincided with those described in the work [3].

**(2*S*,4*R*,4a*R*,8*R*,8a*R*)-2-(3,4-dimethoxyphenyl)-4-fluoro-4,7-dimethyl-3,4,4a,5,8,8a-hexahydro-2*H*-chromen-8-ol ((*R*)-8d):** Yellow viscous oil. $^1$H NMR ($CDCl_3$): δ = 1.35 (d, $^3J$(17, F) = 22.4 Hz, 3 H, 17-H); 1.81 (ddd, $^3J$(4a, F) = 39.2 Hz, $J$(4a,4e) = 14.6 Hz, $J$(4a, 3a) = 11.3 Hz, 1 H, 4-$H_a$); 1.81 (m, all $J \leq 2.5$ Hz, 3 H, 18-H); 1.89 (dddd, $J$(4e, 4a) = 14.6 Hz, $^3J$(4e, F) = 11.2 Hz, $J$(4e,3a) = 3.2 Hz,

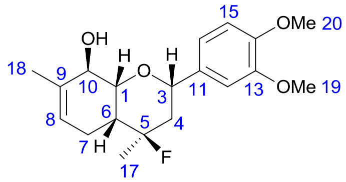


$J$(4e,6a) = 1.0 Hz, 1 H, 4-H$_e$); 1.91-2.06 (m, 3 H, 6-H$_a$, 7-2H); 3.82 (s, 3 H, 20-MeO); 3.85 (s, 3 H, 19-MeO); 3.93 (br.s, 1 H, 10-H$_e$); 4.14 (m, all $J$ < 3.0 Hz, 1 H, 1-H$_e$); 4.68 (dd, $J$(3a,4a) = 11.3 Hz, $J$(3a,4e) = 3.2 Hz, 1 H, 3-H$_a$); 5.55-5.60 (m, 1 H, 8-H); 6.80 (d, $J$(15,16) = 8.2 Hz, 1 H, 15-H); 6.82 (d, $J$(12,16) = 2.0 Hz, 1 H, 12-H); 6.85 (dd, $J$(16,15) = 8.2 Hz, $J$(16,12) = 2.0 Hz, 1 H, 16-H). $^{13}$C NMR (CDCl$_3$) δ = 20.76 (C-18); 24.07 (d, $^3J$(C-F) = 9.7 Hz, C-7); 24.79 (d, $^2J$(C-F) = 24.8 Hz, C-17); 36.10 (d, $^2J$(C-F) = 20.8 Hz, C-6); 40.11 (d, $^2J$(C-F) = 21.2 Hz, C-4); 55.78 (C-19); 55.83 (C-20); 70.12 (C-10); 75.44 (d, $^3J$(C-F) = 1.2 Hz, C-1); 75.58 (C-3); 95.18 (d, $^1J$(C-F)=169.0 Hz, C-5); 109.54 (C-12); 111.13 (C-15); 118.06 (C-16); 123.13 (C-8); 132.20 (C-9); 134.47 (C-11); 148.48 (C-14); 148.85 (C-13). HR-MS: 336.1728 ($M^+$ ($C_{19}H_{25}O_4F)^+$; calc. 336.1731).

**(2*S*,4*S*,4a*R*,8*R*,8a*R*)-2-(3,4-dimethoxyphenyl)-4-fluoro-4,7-dimethyl-3,4,4a,5,8,8a-hexahydro-2*H*-chromen-8-ol ((*S*)-8d):** Yellow viscous oil. $^1$H NMR (CDCl$_3$): δ = 1.63 (dd, $^3J$(17, F) = 23.5 Hz, $J$(17, 4a) = 0.7 Hz, 3 H, 17-H); 1.79 (m, all $J \leq$ 2.5 Hz, 3 H, 18-H); 1.83 (br.dd, $J$(4e, 4a) = 13.3 Hz, $J$(4e,3a) = 2.6 Hz, 1 H, 4-H$_e$); 2.04 (br.dd, $J$(6a, 7a) = 10.6 Hz, $J$(6a,7e) = 6.5, 1 H, 6-H$_a$); 2.17 (ddd, $^3J$(4a, F) = 14.0 Hz, $J$(4a,4e) = 13.3 Hz, $J$(4a,3a) = 12.1 Hz, 1 H, 4-H$_a$); 2.16-2.26 (m, 2 H, 7-H); 3.70 (m, all $J \leq$ 3.0 Hz, 1 H, 1-H$_e$); 3.82 (s, 3 H, 20-MeO); 3.85 (s, 3 H, 19-MeO); 3.89 (br.s, 1 H, 10-H$_e$); 4.31 (dd, $J$(3a,4a) = 12.1 Hz, $J$(3a,4e) = 2.6 Hz, 1 H, 3-H$_a$); 5.60-5.65 (m, 1 H, 8-H); 6.80 (d, $J$(15,16) = 8.2 Hz, 1 H, 15-H); 6.81 (d, $J$(12,16) = 2.0 Hz, 1 H, 12-H); 6.85 (dd, $J$(16,15) = 8.2 Hz, $J$(16,12) = 2.0 Hz, 1 H, 16-H). $^{13}$C NMR (CDCl$_3$) δ = 20.60 (C-18); 22.46 (d, $^3J$(C-F) = 6.2 Hz, C-7); 24.76 (d, $^2J$(C-F) = 26.0 Hz, C-17); 37.71 (d, $^2J$(C-F) = 19.0 Hz, C-6); 40.47 (d, $^2J$(C-F) = 20.3 Hz, C-4); 55.76, 55.79 (C-19, C-20); 70.05 (d, $^4J$(C-F) = 1.8 Hz, C-10); 77.43 (d, $^3J$(C-F) = 11.5 Hz, C-3); 77.91 (d, $^3J$(C-F) = 7.5 Hz, C-1); 95.13 (d, $^1J$(C-F)=176.0 Hz, C-5); 109.57 (C-12); 111.06 (C-15); 118.25 (C-16); 124.26 (C-8); 131.22 (C-9); 133.71 (C-11); 148.65 (C-14); 148.84 (C-13).

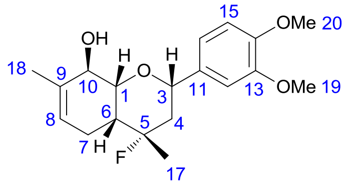


**Reaction of 1 with 2,4,6-trimethoxybenzaldehyde (6e):** The reaction of diol **1** and aldehyde **6e** for 8 hours at 2 °C gave products **2e** (*R*:*S* = 1:1, 35%), **8e** (*R*:*S* = 10:1, 42%). Spectral characteristics of compounds **2e** have coincided with those described in the work [4].

**(2*S*,4*R*,4a*R*,8*R*,8a*R*)-4-fluoro-4,7-dimethyl-2-(2,4,6-trimethoxyphenyl) -3,4,4a,5,8,8a-hexahydro-2*H*-chromen-8-ol ((*R*)-8e):** Brown viscous oil. $^1$H NMR (CDCl$_3$): δ = 1.34 (d, $^3J$(17, F) = 22.2 Hz, 3 H, 17-H); 1.54 (dddd, $J$(4e,4a) = 14.6 Hz, $^3J$(4e,F) = 11.5 Hz, $J$(4e, 3a) = 3.2 Hz, $J$(4e,6a) = 1.3 Hz, 1 H, 4-H$_e$); 1.77 (m, all $J \leq$ 2.5 Hz, 3 H, 18-H); 1.87-2.00 (m, 2 H, 6-H$_a$, 7-H$_e$); 2.14-2.23

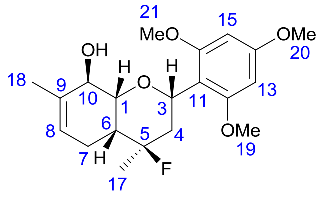


(m, 1 H, 7-H$_a$); 2.60 (ddd, $^3J$(4a,F) = 42.0 Hz, $J$(4a,4e) = 14.6 Hz, $J$(4a,3a) = 12.1 Hz, 1 H, 4-H$_a$); 3.73 (s, 6 H, 19-MeO, 21-MeO); 3.75 (s, 3H, 20-MeO); 3.90 (br.s, 1 H, 10-H$_e$); 4.09 (m, all $J \leq 3.0$ Hz, 1 H, 1-H$_e$); 5.31 (dd, $J$(3a,4a) = 12.1 Hz, $J$(3a,4e) = 3.2 Hz, 1H, 3-H$_a$); 5.56-5.60 (m, 1 H, 8-H); 6.09 (s, 2 H, 13-H, 15-H). $^{13}$C NMR (CDCl$_3$) δ = 20.71 (C-18); 24.00 (d, $^3J$(C-F) = 9.3 Hz, C-7); 25.06 (d, $^2J$(C-F) = 24.8 Hz, C-17); 35.52 (d, $^2J$(C-F) = 20.8 Hz, C-4); 36.62 (d, $^2J$(C-F) = 20.8 Hz, C-6); 55.16 (C-20); 56.10 (C-19, C-21); 67.65 (C-3); 70.34 (C-10); 75.20 (d, $^3J$(C-F) = 1.8 Hz, C-1); 91.97 (C-13, C-15); 95.84 (d, $^1J$(C-F)=167.6 Hz, C-5); 110.45 (C-11); 123.66 (C-8); 132.02 (C-9); 159.74 (C-14); 160.78 (C-12, C-16). HR-MS: 366.1836 ($M^+$ $(C_{20}H_{27}O_5F)^+$; calc. 366.1837).

**Reaction of 1 with 2,4,5-trimethoxybenzaldehyde (6f):** The reaction of diol **1** and aldehyde **6f** for 8 hours at 2 °C gave products **2f** (*R*:*S* = 1:3, 8%), **8f** (*R*:*S* = 7:1, 20%) and **9f** (14%). Spectral characteristics of compounds **2f** and **9f** have coincided with those described in the work [4].

**(2*S*,4*R*,4a*R*,8*R*,8a*R*)-4-fluoro-4,7-dimethyl-2-(2,4,5-trimethoxyphenyl) -3,4,4a,5,8,8a-hexahydro-2*H*-chromen-8-ol ((*R*)-8f):** Brown viscous oil. $^1$H NMR (CDCl$_3$): δ = 1.34 (d, $^3J$(17, F) = 22.3 Hz, 3 H, 17-H); 1.72 (ddd, $J$(4a,F) = 40.7 Hz, $J$(4a,4e) = 14.7 Hz, $J$(4a,3a) = 11.6 Hz, 1 H, 4-H$_a$); 1.83 (m, all $J \leq 2.5$ Hz, 3 H, 18-H); 1.92 (ddd, $J$(4e,4a) = 14.7 Hz, $J$(4e,F) = 10.8 Hz, $J$(4e,3a) = 2.8 Hz, 1 H, 4-H$_e$); 1.93-2.05 (m, 3 H, 6-H, 7-2H); 3.78 (s, 3 H, 19-MeO); 3.80 (s, 3 H, 21-MeO); 3.84 (s, 3 H, 20-MeO); 3.93 (br.s, 1 H, 10-H$_e$); 4.16 (m, all $J \leq 3.0$ Hz, 1 H, 1-H$_e$); 5.05 (dd, $J$(3a,4a) = 11.6 Hz, $J$(3a,4e) = 2.8 Hz, 1 H, 3-H$_a$); 5.57-5.61 (m, 1 H, 8-H); 6.48 (s, 1 H, 13-H); 6.88 (s, 1 H, 16-H). $^{13}$C NMR (CDCl$_3$) δ = 20.81 (C-18); 24.13 (d, $^3J$(C-F) = 9.3 Hz, C-7); 24.81 (d, $^2J$(C-F) = 24.3 Hz, C-17); 36.25 (d, $^2J$(C-F) = 20.8 Hz, C-6); 38.89 (d, $^2J$(C-F) = 21.7 Hz, C-4); 56.09, 56.49, 56.88 (C-19, C-20, C-21); 70.24 (C-10); 70.27 (C-3); 75.35 (d, $^3J$(C-F) = 1.3 Hz, C-1); 95.28 (d, $^1J$(C-F) = 168.5 Hz, C-5); 97.83 (C-13); 111.33 (C-16); 122.23 (C-11); 123.23 (C-8); 132.23 (C-9); 143.30 (C-15); 149.08 (C-14); 150.50 (C-12). HR-MS: 366.1842 ($M^+$ $(C_{20}H_{27}O_5F)^+$; calc. 366.1837).

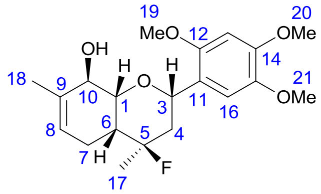


**Reaction of 1 with 4-nitrobenzaldehyde (6g):** The reaction of diol **1** and aldehyde **6g** for 72 hours at 2 °C gave products **2g** (*R*:*S* = 1:1, 17%), **8g** (*R*:*S* = 12:1, 53%).

**(2*S*,4*S*,4a*R*,8*R*,8a*R*)-4,7-dimethyl-2-(4-nitrophenyl)-3,4,4a,5,8,8a-hexahydro-2*H*-chromene-4,8-diol ((*S*)-2g):** Yellow oil. $^1$H NMR (CDCl$_3$): δ = 1.53 (s, 3 H, 17-H); 1.70 (ddd, $J$(4e,4a) = 13.3

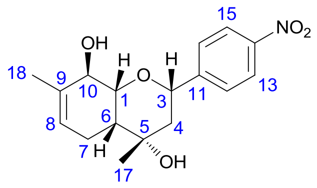

Hz, $J$(4e,3a) = 3.0 Hz, $J$(4e,6a) = 1.1 Hz, 1 H, 4-$H_e$); 1.75-1.81 (1H, m, 4-$H_a$); 1.81 (m, all $J \leq 2.5$ Hz, 3 H, 18-H); 1.86 (br.dd, $J$(6a,7a) = 10.7 Hz, $J$(6a,7e) = 6.7 Hz, 1 H, 6-$H_a$); 2.05-2.22 (m, 2 H, 7-H); 3.82 (br.dd, both $J$~ 2.2 Hz, 1 H, 1-$H_e$); 3.90 (br.s, 1 H, 10-$H_e$); 4.51 (dd, $J$(3a,4a) = 11.8 Hz, $J$(3a,4e) = 3.0 Hz, 1 H, 3-$H_a$); 5.62-5.65 (m, 1 H, 8-H); 7.43 (br.d, $J$ = 8.9 Hz, 2 H, 12-H, 16-H); 8.13 (br.d, $J$ = 8.9 Hz, 2 H, 13-H, 15-H). $^{13}$C NMR ($CDCl_3$) δ = 20.65 (C-18); 22.58 (C-7); 26.93 (C-17); 38.13 (C-6); 42.96 (C-4); 70.35 (C-10); 70.83 (C-5); 76.34 (C-3); 77.64 (C-1); 123.47 (C-13, C-15); 124.43 (C-8); 126.29 (C-12, C-16); 131.34 (C-9); 147.12 (C-14); 149.24 (C-11). HR-MS: 319.1421 ($M^+$ ($C_{17}H_{21}O_5N$)$^+$; calc. 319.1414).

**(2*S*,4*R*,4a*R*,8*R*,8a*R*)-4,7-dimethyl-2-(4-nitrophenyl)-3,4,4a,5,8,8a-hexahydro-2*H*-chromene-4,8-diol ((*R*)-2g):** $^1$H NMR ($CDCl_3$): δ = 1.22 (s, 3 H, 17-H); 1.61 (dd, $J$(4a,4e) = 14.0 Hz, $J$(4a,3a) = 11.3 Hz, 1 H, 4-$H_a$); 1.67 (ddd, $J$(4e,4a) = 14.0 Hz, $J$(4e,3a) = 3.2 Hz, $J$(4e,6a) = 1.2 Hz, 1 H, 4-$H_e$); 1.73 (br.dd, $J$(6a,7a) = 10.8 Hz, $J$(6a,7e) = 6.7 Hz, 1 H, 6-$H_a$); 1.81 (m, all $J \leq 2.5$ Hz, 3 H, 18-H); 1.88-2.04 (m, 2 H, 7-H); 3.92 (br.s, 1 H, 10-$H_e$); 4.26 (br.dd, both $J$~ 2.2 Hz, 1 H, 1-$H_e$); 4.88 (dd, $J$(3a,4a) = 11.3 Hz, $J$(3a,4e) = 3.2 Hz, 1 H, 3-$H_a$); 5.56-5.59 (m, 1 H, 8-H); 7.43 (br.d, $J$ = 8.9 Hz, 2 H, 12-H, 16-H); 8.10 (br.d, $J$ = 8.9 Hz, 2 H, 13-H, 15-H). $^{13}$C NMR ($CDCl_3$) δ = 20.74 (C-18); 24.43 (C-7); 28.16 (C-17); 37.84 (C-6); 41.93 (C-4); 70.36 (C-10); 70.60 (C-5); 74.73 (C-3); 75.10 (C-1); 123.39 (C-13, C-15); 123.81 (C-8); 126.24 (C-12, C-16); 131.81 (C-9); 146.95 (C-14); 150.13 (C-11).

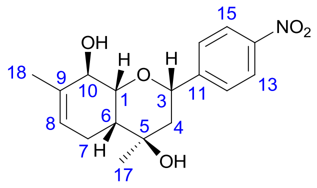

**(2*S*,4*R*,4a*R*,8*R*,8a*R*)-4-fluoro-4,7-dimethyl-2-(4-nitrophenyl)-3,4,4a,5,8,8a-hexahydro-2*H*-chromen-8-ol ((*R*)-8g):** Yellow oil. $^1$H NMR ($CDCl_3$): δ = 1.35 (d, $^3J$(17, F) = 22.4 Hz, 3 H, 17-H); 1.69 (ddd, $^3J$(4a,F) = 39.5 Hz, $J$(4a,4e) = 14.5 Hz, $J$(4a,3a) = 11.8 Hz, 1 H, 4-$H_a$); 1.83 (m, all $J \leq 2.5$ Hz, 3 H, 18-H); 1.84-1.92 (m, 1 H, 7-$H_a$); 1.95 (dddd, $J$(4e,4a) = 14.5 Hz, $^3J$(4e,F) = 10.0 Hz, $J$(4e,3a) = 2.9 Hz, $J$(4e,6a) = 1.2 Hz, 1 H, 4-$H_e$); 1.99-2.07 (m, 2 H, 6-$H_a$, 7-$H_e$); 3.93 (br.s, 1 H, 10-$H_e$); 4.16 (m, all $J \leq 3.0$ Hz, 1 H, 1-$H_e$); 4.84 (dd, $J$(3a,4a) = 11.8 Hz, $J$(3a,4e) = 2.9 Hz, 1 H, 3-$H_a$); 5.56-5.60 (m, 1 H, 8-H); 7.45 (dd, $J$(12,13) = $J$(16,15) = 8.8 Hz, $J$(12,3a) = $J$(16,3a) = 0.6 Hz, 2 H, 12-H, 16-H); 8.14 (d, $J$(13,12) = $J$(15,16) = 8.8 Hz, 2 H, 13-H, 15-H). $^{13}$C NMR ($CDCl_3$) δ = 20.76 (C-18); 23.94 (d, $^3J$(C-F) = 9.3 Hz, C-7); 24.65 (d, $^2J$(C-F) = 24.3 Hz, C-17); 35.84 (d, $^2J$(C-F) = 20.8 Hz, C-6); 40.17 (d, $^2J$(C-F) = 21.7 Hz, C-4); 69.91 (C-10); 74.60 (C-3); 75.27 (d, $^3J$(C-F) = 1.3 Hz, C-1); 94.81 (d, $^1J$(C-F) = 169.8 Hz, C-5); 123.0 (C-8); 123.48 (C-13, C-15);

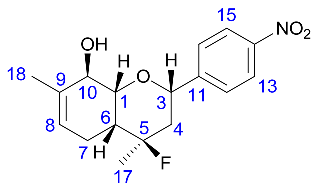

126.23 (C-12, C-16); 132.17 (C-9); 147.11 (C-14); 149.31 (C-11). HR-MS: 321.1369 ($M^+$ $(C_{17}H_{20}O_4NF)^+$; calc. 321.1371).

**Reaction of 1 with 4-fluorobenzaldehyde (6h):** The reaction of diol **1** and aldehyde **6h** for 72 hours at 2 °C gave products **2h** (*R*:*S* = 1:2, 17%), **8h** (*R*:*S* = 6:1, 47%).

**(2*S*,4*S*,4a*R*,8*R*,8a*R*)-2-(4-fluorophenyl)-4,7-dimethyl-3,4,4a,5,8,8a-hexahydro-2*H*-chromene-4,8-diol ((*S*)-2h):** Yellow oil. $^1$H NMR ($CDCl_3$+$CD_3OD$): δ = 1.41 (s, 3 H, 17-H); 1.54 (br. dd, *J*(4e,4a) = 13.3 Hz, *J*(4e,3a) = 2.5 Hz, 1 H, 4-$H_e$); 1.71 (br.s, 3 H, 18-H); 1.74 (br.t, *J*(6a,7) = 8.7 Hz, 1 H, 6-$H_a$); 1.78 (dd, *J*(4a,4e) = 13.3 Hz, *J*(4a,3a) = 12.1 Hz, 1 H, 4-$H_a$); 2.03-2.11 (m, 2 H, 7-H); 3.70 (dd, *J*(1e,6a) ≈ *J*(1e,10e) ≈ 2.2 Hz, 1 H, 1-$H_e$); 3.76 (br.s, 1 H, 10-$H_e$); 4.32 (dd, *J*(3a,4a) = 12.1 Hz, *J*(3a,4e) = 2.5 Hz, 1 H, 3-$H_a$); 5.53-5.57 (m, 1 H, 8-H); 6.89 (dd, *J*(13,12) = $^3J$(13,F) = *J*(15,16) = $^3J$(15,F) = 8.8 Hz, 2 H, 13-H, 15-H); 7.18 (dd, *J*(12,13) = *J*(16,15) = 8.8 Hz, $^4J$(12,F) = $^4J$(16,F) = 5.5 Hz, 2 H, 12-H, 16-H). $^{13}$C NMR ($CDCl_3$+$CD_3OD$) δ = 20.37 (C-18); 22.50 (C-7); 26.39 (C-17); 38.02 (C-6); 42.50 (C-4); 69.90 (C-10); 70.38 (C-5); 76.76 (C-3); 77.79 (C-1); 114.84 (d, $^2J$(C-F) = 21.6 Hz, C-13, C-15); 124.13 (C-8); 127.38 (d, $^3J$(C-F) = 8.3 Hz, C-12, C-16); 131.07 (C-9); 137.67 (d, $^4J$(C-F) = 3.3 Hz, C-11); 161.94 (d, $^1J$(C-F) =245.1 Hz, C-14); HR-MS: 275.1440 ($[M\text{-}OH]^+$ $(C_{17}H_{20}O_2F)^+$; calc. 275.1442).

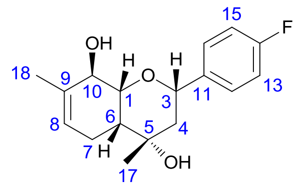

**(2*S*,4*R*,4a*R*,8*R*,8a*R*)-2-(4-fluorophenyl)-4,7-dimethyl-3,4,4a,5,8,8a-hexahydro-2*H*-chromene-4,8-diol ((*R*)-2h):** Yellow oil. $^1$H NMR ($CDCl_3$+$CD_3OD$): δ = 1.12 (s, 3 H, 17-H); 1.51 (ddd, *J*(4e,4a) = 14.1 Hz, *J*(4e,3a) = 3.0 Hz, *J*(4e,6a) = 1.3 Hz, 1 H, 4-$H_e$); 1.60 (dd, *J*(4a,4e) = 14.1 Hz, *J*(4a,3a) = 11.5 Hz, 1 H, 4-$H_a$); 1.58-1.63 (m, 1 H, 6-$H_a$); 1.71 (br.s, 3 H, 18-H); 1.86-1.96 (m, 2 H, 7-H); 3.77 (br.s, 1 H, 10-$H_e$); 4.12 (dd, *J*(1e,6a) ≈ *J*(1e,10e) ≈ 2.2 Hz, 1 H, 1-$H_e$); 4.67 (dd, *J*(3a,4a) = 11.5 Hz, *J*(3a,4e) = 3.0 Hz, 1 H, 3-$H_a$); 5.47-5.51 (m, 1 H, 8-H); 6.88 (dd, *J*(13,12) = $^3J$(13,F) = *J*(15,16) = $^3J$(15,F) = 8.8 Hz, 2 H, 13-H, 15-H); 7.18 (dd, *J*(12,13) = *J*(16,15) = 8.8 Hz, $^4J$(12,F) = $^4J$(16,F) = 5.5 Hz, 2 H, 12-H, 16-H). $^{13}$C NMR ($CDCl_3$+$CD_3OD$) δ = 20.44 (C-18); 24.32 (C-7); 27.61 (C-17); 37.47 (C-6); 41.75 (C-4); 69.90 (C-10); 70.05 (C-5); 75.12 (C-3); 75.24 (C-1); 114.76 (d, $^2J$(C-F) = 21.3 Hz, C-13, C-15); 123.51 (C-8); 127.32 (d, $^3J$(C-F) = 8.3 Hz, C-12, C-16); 131.66 (C-9); 138.31 (d, $^4J$(C-F) = 3.3 Hz, C-11); 161.82 (d, $^1J$(C-F) = 245.0 Hz, C-14).

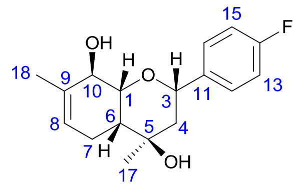

**(2*S*,4*R*,4a*R*,8*R*,8a*R*)-4-fluoro-2-(4-fluorophenyl)-4,7-dimethyl-3,4,4a,5,8,8a-hexahydro-2*H*-chromen-8-ol ((*R*)-8h):** Yellow oil.$^1$H NMR ($CDCl_3$): δ = 1.35 (d, $^3J$(17, F) = 22.4 Hz, 3 H, 17-H);

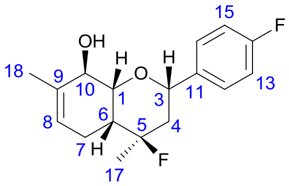


1.75 (ddd, $^3J$(4a, F) = 39.9 Hz, $J$(4a,4e) = 14.6 Hz, $J$(4a, 3a) = 11.7 Hz, 1 H, 4-H$_a$); 1.82 (m, all $J \leq 2.5$ Hz, 3 H, 18-H); 1.89 (dddd, $J$(4e,4a) = 14.6 Hz, $^3J$(4e,F) = 10.4 Hz, $J$(4e,3a) = 2.9 Hz, $J$(4e,6a) = 1.2 Hz, 1 H, 4-H$_e$); 1.88-1.96 (m, 1 H, 7-H$_a$); 1.97-2.06 (m, 2 H, 6-H$_a$, 7-H$_e$); 3.92 (br.s, 1 H, 10-H$_e$); 4.14 (m, all $J \leq 3.0$ Hz, 1 H, 1-H$_e$); 4.73 (dd, $J$(3a,4a) = 11.7 Hz, $J$(3a,4e) = 2.9 Hz, 1 H, 3-H$_a$); 5.56-5.60 (m, 1 H, 8-H); 6.98 (dd, $J$(13,12) = $J$(15,16) = 8.8 Hz, $^3J$(13,F) = $^3J$(15,F) = 8.8 Hz, 2 H, 13-H, 15-H); 7.26 (dd, $J$(12,13) = $J$(16,15) = 8.8 Hz, $^4J$(12,F) = $^4J$(16,F) = 5.4 Hz, 2 H, 12-H, 16-H). $^{13}$C NMR ($CDCl_3$) δ = 20.78 (C-18); 24.04 (d, $^3J$(C-F) = 9.4 Hz, C-7); 24.73 (d, $^2J$(C-F) = 24.4 Hz, C-17); 35.98 (d, $^2J$(C-F) = 20.8 Hz, C-6); 40.38 (d, $^2J$(C-F) = 21.5 Hz, C-4); 70.05 (C-10); 75.04 (C-3); 75.33 (d, $^3J$(C-F) = 1.2 Hz, C-1); 95.05 (d, $^1J$(C-F) = 168.8 Hz, C-5); 115.07 (d, $^2J$(C-F)=21.3 Hz, C-13, C-15); 123.12 (C-8); 127.37 (d, $^3J$(C-F) = 8.1 Hz, C-12, C-16); 132.19 (C-9); 137.73 (d, $^4J$(C-F) = 3.1 Hz, C-11); 162.06 (d, $^1J$(C-F) = 245.4 Hz, C-14). HR-MS: 294.1424 ($M^+$ $(C_{17}H_{20}O_2F_2)^+$; calc. 294.1426).

**Reaction of 1 with 4-chlorobenzaldehyde (6i):** The reaction of diol **1** and aldehyde **6i** for 72 hours at 2 °C gave products **2i** (*R*:*S* = 1:3, 17%), **8i** (*R*:*S* = 6:1, 58%).

**(2*S*,4*S*,4a*R*,8*R*,8a*R*)-2-(4-chlorophenyl)-4,7-dimethyl-3,4,4a,5,8,8a-hexahydro-2*H*-chromene-4,8-diol ((*S*)-2i):** Transparent viscous oil. $^1$H NMR ($CDCl_3$+$CD_3OD$): δ = 1.16 (s, 3 H, 17-H); 1.29

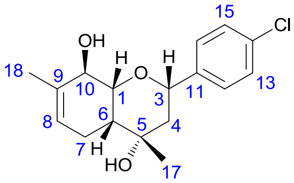


(ddd, $J$(4e,4a) = 13.4 Hz, $J$(4e,3a) = 2.7 Hz, $J$(4e,6a) = 1.1 Hz, 1 H, 4-H$_e$); 1.45 (m, all $J \leq 2.5$ Hz, 3 H, 18-H); 1.48-1.54 (m, 2 H, 4-H$_a$, 6-H$_a$); 1.77-1.89 (m, 2 H, 7-H); 3.45 (dd, $J$(1e,10e) = 2.4 Hz, $J$(1e,6a) = 2.2 Hz, 1 H, 1-H$_e$); 3.48 (br.s, 1 H, 10-H$_e$); 4.10 (dd, $J$(3a,4a) = 12.1 Hz, $J$(3a,4e) = 2.7 Hz, 1 H, 3-H$_a$); 5.27-5.31 (m, 1 H, 8-H); 6.90-6.94 (m, 4 H, 12-H, 13-H, 15-H, 16-H). $^{13}$C NMR ($CDCl_3$+$CD_3OD$) δ = 19.67 (C-18); 22.09 (C-7); 25.59 (C-17); 37.63 (C-6); 41.90 (C-4); 69.37 (C-10); 69.72 (C-5); 76.27 (C-3); 77.53 (C-1); 123.65 (C-8); 126.66 (C-12, C-16); 127.63 (C-13, C-15); 130.59 (C-9); 132.41 (C-14); 140.27 (C-11). HR-MS: 308.1178 ($M^+$ $(C_{17}H_{21}O_3Cl)^+$; calc. 308.1174).

**(2*S*,4*R*,4a*R*,8*R*,8a*R*)-2-(4-chlorophenyl)-4,7-dimethyl-3,4,4a,5,8,8a-hexahydro-2*H*-chromene-4,8-diol ((*R*)-2i):** Transparent viscous oil. $^1$H NMR ($CDCl_3$+$CD_3OD$): δ = 1.05 (s, 3 H, 17-H); 1.46

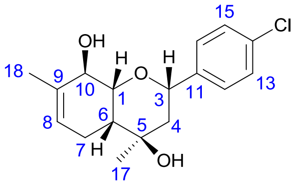


(ddd, $J$(4e,4a) = 14.1 Hz, $J$(4e,3a) = 3.2 Hz, $J$(4e,6a) = 1.1 Hz, 1 H, 4-H$_e$); 1.51 (dd, $J$(4a,4e) = 14.1 Hz, $J$(4a,3a) = 11.4 Hz, 1 H, 4-H$_a$); 1.53 (dddd, $J$(6a,7a) = 10.8 Hz, $J$(6a,7e) = 6.7 Hz, $J$(6a,1e) = 2.1 Hz, $J$(6a,4e) = 1.1 Hz, 1 H, 6-H$_a$); 1.64 (m, all $J \leq 2.5$ Hz, 3 H, 18-H); 1.75-1.91 (m, 2 H, 7-H);

3.69 (br.s, 1 H, 10-$H_e$); 4.04 (dd, *J*(1e,10e) = 2.3 Hz, *J*(1e,6a) = 2.1 Hz, 1 H, 1-$H_e$); 4.60 (dd, *J*(3a,4a) = 11.4 Hz, *J*(3a,4e) = 3.2 Hz, 1 H, 3-$H_a$); 5.45-5.50 (m, 1 H, 8-H); 7.06-7.13 (m, 4 H, 12-H, 13-H, 15-H, 16-H). $^{13}$C NMR ($CDCl_3$+$CD_3OD$) δ = 20.18 (C-18); 24.18 (C-7); 27.34 (C-17); 37.26 (C-6); 41.54 (C-4); 69.83 (C-10); 70.13 (C-5); 75.17 (C-3); 76.64 (C-1); 123.33 (C-8); 126.92 (C-12, C-16); 127.98 (C-13, C-15); 131.54 (C-9); 132.53 (C-14); 141.01 (C-11).

**(2*S*,4*R*,4a*R*,8*R*,8a*R*)-2-(4-chlorophenyl)-4-fluoro-4,7-dimethyl-3,4,4a,5,8,8a-hexahydro-2*H*-chromen-8-ol ((*R*)-8i):** Yellow viscous oil. $^1$H NMR ($CDCl_3$): δ = 1.35 (d, $^3J$(17, F) = 22.4 Hz, 3 H, 17-H); 1.73 (ddd, $^3J$(4a,F) = 39.9 Hz, *J*(4a,4e) = 14.5 Hz, *J*(4a,3a) = 11.7 Hz, 1 H, 4-$H_a$); 1.83 (m, all *J* ≤ 2.5 Hz, 3 H, 18-H); 1.89 (dddd, *J*(4e,4a) = 14.5 Hz, $^3J$(4e,F) = 10.2 Hz, *J*(4e,3a) = 2.9 Hz, *J*(4e,6a) = 1.1 Hz, 1 H, 4-$H_e$); 1.87-1.95 (m, 1 H, 7-$H_a$); 1.97-2.06 (m, 2 H, 6-$H_a$, 7-$H_e$); 3.93 (br.s, 1 H, 10-$H_e$); 4.14 (m, all *J* ≤ 3.0 Hz, 1 H, 1-$H_e$); 4.72 (dd, *J*(3a,4a) = 11.7 Hz, *J*(3a,4e) = 2.9 Hz, 1 H, 3-$H_a$); 5.56-5.60 (m, 1 H, 8-H); 7.23 (d, *J*(12,13) = *J*(16,15) = 8.5 Hz, 2 H, 12-H, 16-H); 7.27 (d, *J*(13,12) = *J*(15,16) = 8.5 Hz, 2 H, 13-H, 15-H). $^{13}$C NMR ($CDCl_3$) δ = 20.78 (C-18); 24.03 (d, $^3J$(C-F) = 9.3 Hz, C-7); 24.74 (d, $^2J$(C-F) = 24.3 Hz, C-17); 35.98 (d, $^2J$(C-F) = 20.8 Hz, C-6); 40.29 (d, $^2J$(C-F) = 21.23 Hz, C-4); 70.07 (C-10); 74.97 (C-3); 75.30 (d, $^3J$(C-F) = 1.1 Hz, C-1); 94.94 (d, $^1J$(C-F) = 168.5 Hz, C-5); 123.13 (C-8); 127.05 (C-12, C-16); 128.41 (C-13, C-15); 132.19 (C-9); 133.10 (C-14); 140.47 (C-11). HR-MS: 310.1133 ($M^+$ $(C_{17}H_{20}O_2FCl)^+$; calc. 310.1130).

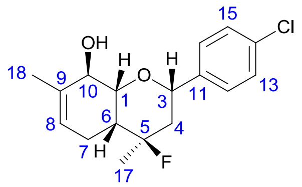


**(2*S*,4*S*,4a*R*,8*R*,8a*R*)-2-(4-chlorophenyl)-4-fluoro-4,7-dimethyl-3,4,4a,5,8,8a-hexahydro-2*H*-chromen-8-ol ((*S*)-8i):** Yellow viscous oil. $^1$H NMR ($CDCl_3$): δ = 1.64 (d, $^3J$(17, F) = 23.4 Hz, 3 H, 17-H); 1.81 (m, all *J* ≤ 2.5 Hz, 3 H, 18-H); 1.82 (br.dd, *J*(4e,4a) = 13.3 Hz, *J*(4e,3a) = 2.6 Hz, 1 H, 4-$H_e$); 1.98-2.12 (m, 2 H, 4-$H_a$, 6-$H_a$); 2.16-2.22 (m, 2 H, 7-H); 3.71 (m, all *J* ≤ 3.0 Hz, 1 H, 1-$H_e$); 3.90 (br.s, 1 H, 10-$H_e$); 4.35 (dd, *J*(3a,4a) = 12.1 Hz, *J*(3a,4e) = 2.6 Hz, 1 H, 3-$H_a$); 5.63-5.67 (m, 1 H, 8-H); 7.23 (d, *J*(12,13) = *J*(16,15) = 8.5 Hz, 2 H, 12-H, 16-H); 7.28 (d, *J*(13,12) = *J*(15,16) = 8.5 Hz, 2 H, 13-H, 15-H). $^{13}$C NMR ($CDCl_3$) δ = 20.65 (C-18); 22.46 (d, $^3J$(C-F) = 6.2 Hz, C-7); 24.73 (d, $^2J$(C-F) = 26.1 Hz, C-17); 37.62 (d, $^2J$(C-F) = 19.0 Hz, C-6); 40.82 (d, $^2J$(C-F) = 20.8 Hz, C-4); 70.07 (d, $^4J$(C-F) = 1.8 Hz, C-10); 76.82 (d, $^3J$(C-F) = 11.5 Hz, C-3); 77.81 (d, $^3J$(C-F) = 7.5 Hz, C-1); 94.88 (d, $^1J$(C-F) = 176.9 Hz, C-5); 124.32 (C-8); 127.11 (C-12, C-16); 128.47 (C-13, C-15); 131.23 (C-9); 133.38 (C-14); 139.76 (C-11).

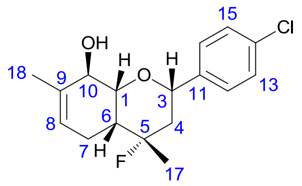

**Reaction of 1 with 4-bromobenzaldehyde(6j):** The reaction of diol **1** and aldehyde **6j** for 72 hours at 2 °C gave products **2j** (*R*:*S* = 1:2, 13%), **8j** (*R*:*S* = 10:1, 60%).

**(2*S*,4*S*,4a*R*,8*R*,8a*R*)-2-(4-bromophenyl)-4,7-dimethyl-3,4,4a,5,8,8a-hexahydro-2*H*-chromene-4,8-diol ((*S*)-2j):** Yellow viscous oil. $^{1}$H NMR ($CD_3OD$): δ = 1.54 (s, 3 H, 17-H); 1.67 (ddd, *J*(4e,4a) = 13.4 Hz, *J*(4e,3a) = 2.8 Hz, *J*(4e,6a) = 1.2 Hz, 1 H, 4-$H_e$); 1.83 (m, all *J* ≤ 2.5 Hz, 3 H, 18-H); 1.85 (dd, *J*(4a,4e) = 13.4 Hz, *J*(4a,3a) = 12.0 Hz, 1 H, 4-$H_a$); 1.88-1.93 (m, 1 H, 6-$H_a$); 2.15-2.21 (m, 2 H, 7-H); 3.82-3.85 (m, 2 H, 1-$H_e$, 10-$H_e$); 4.51 (dd, *J*(3a,4a) = 12.0 Hz, *J*(3a,4e) = 2.8 Hz, 1 H, 3-$H_a$); 5.65-5.69 (m, 1 H, 8-H); 7.26 (d, *J*(12,13) = *J*(16,15) = 8.5 Hz, 2 H, 12-H, 16-H); 7.48 (d, *J*(13,12) = *J*(15,16) = 8.5 Hz, 2 H, 13-H, 15-H). $^{13}$C NMR ($CD_3OD$) δ = 21.13 (C-18); 24.02 (C-7); 27.09 (C-17); 39.67 (C-6); 43.97 (C-4); 71.38 (C-10); 71.53 (C-5); 78.12 (C-3); 79.49 (C-1); 122.04 (C-14); 125.40 (C-8); 128.86 (C-12, C-16); 132.37 (C-13, C-15); 132.62 (C-9); 143.25 (C-11). HR-MS: 334.0569 ($[M\text{-}H_2O]^+$ $(C_{17}H_{19}O_2Br)^+$; calc. 334.0563).

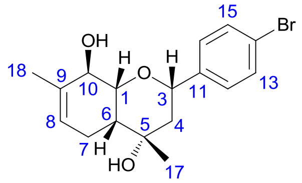


**(2*S*,4*R*,4a*R*,8*R*,8a*R*)-2-(4-bromophenyl)-4,7-dimethyl-3,4,4a,5,8,8a-hexahydro-2*H*-chromene-4,8-diol ((*R*)-2j):** Yellow viscous oil. $^{1}$H NMR ($CD_3OD$): δ = 1.24 (s, 3 H, 17-H); 1.63 (ddd, *J*(4e,4a) = 14.1 Hz, *J*(4e,3a) = 3.0 Hz, *J*(4e,6a) = 1.2 Hz, 1 H, 4-$H_e$); 1.73 (dd, *J*(4a,4e) = 14.1 Hz, *J*(4a,3a) = 11.6 Hz, 1 H, 4-$H_a$); 1.77-1.82 (m, 1 H, 6-$H_a$); 1.83 (m, all *J* ≤ 2.5 Hz, 3 H, 18-H); 1.96-2.10 (m, 2 H, 7-H); 3.83 (br.s, 1 H, 10-$H_e$); 4.26 (dd, *J*(1e,10e) = 2.3 Hz, *J*(1e,6a) = 2.1 Hz, 1 H, 1-$H_e$); 4.77 (dd, *J*(3a,4a) = 11.6 Hz, *J*(3a,4e) = 3.0 Hz, 1 H, 3-$H_a$); 5.60-5.64 (m, 1 H, 8-H); 7.25 (d, *J*(12,13) = *J*(16,15) = 8.5 Hz, 2 H, 12-H, 16-H); 7.47 (d, *J*(13,12) = *J*(15,16) = 8.5 Hz, 2 H, 13-H, 15-H). $^{13}$C NMR ($CD_3OD$) δ = 22.22 (C-18); 25.64 (C-7); 28.23 (C-17); 38.71 (C-6); 43.15 (C-4); 71.19 (C-5); 71.35 (C-10); 76.61 (C-3); 77.08 (C-1); 121.85 (C-14); 124.83 (C-8); 128.88 (C-12, C-16); 132.33 (C-13, C-15); 133.10 (C-9); 143.70 (C-11).

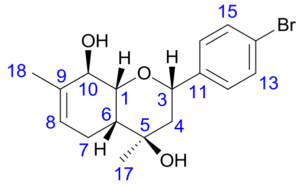


**(2*S*,4*R*,4a*R*,8*R*,8a*R*)-2-(4-bromophenyl)-4-fluoro-4,7-dimethyl-3,4,4a,5,8,8a-hexahydro-2*H*-chromen-8-ol ((*R*)-8j):** Yellow viscous oil. $^{1}$H NMR ($CDCl_3$): δ = 1.35 (d, $^{3}J$(17, F) = 22.3 Hz, 3 H, 17-H); 1.72 (ddd, $^{3}J$(4a,F) = 39.8 Hz, *J*(4a,4e) = 14.6 Hz, *J*(4a,3a) = 11.7 Hz, 1 H, 4-$H_a$); 1.83 (m, all *J* ≤ 2.5 Hz, 3 H, 18-H); 1.89 (dddd, *J*(4e,4a) = 14.6 Hz, $^{3}J$(4e,F) = 10.2 Hz, *J*(4e,3a) = 3.0 Hz, *J*(4e,6a) = 1.1 Hz, 1 H, 4-$H_e$); 1.87-1.95 (m, 1 H, 7-$H_a$); 1.97-2.07 (m, 2 H, 6-$H_a$, 7-$H_e$); 3.93 (br.s, 1 H, 10-$H_e$); 4.14 (m, all *J* ≤ 3.0 Hz, 1 H, 1-$H_e$); 4.71 (dd, *J*(3a,4a) = 11.7

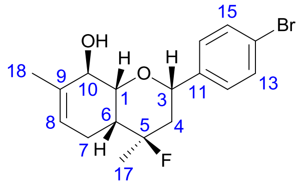

Hz, $J$(3a,4e) = 2.9 Hz, 1 H, 3-H$_a$); 5.56-5.60 (m, 1 H, 8-H); 7.17 (d, $J$(12,13) = $J$(16,15) = 8.5 Hz, 2 H, 12-H, 16-H); 7.42 (d, $J$(13,12) = $J$(15,16) = 8.5 Hz, 2 H, 13-H, 15-H). $^{13}$C NMR (CDCl$_3$) δ = 20.79 (C-18); 24.04 (d, $^3J$(C-F) = 9.3 Hz, C-7); 24.74 (d, $^2J$(C-F) = 24.3 Hz, C-17); 35.99 (d, $^2J$(C-F) = 20.8 Hz, C-6); 40.26 (d, $^2J$(C-F) = 21.2 Hz, C-4); 70.09 (C-10); 75.01 (C-3); 75.30 (d, $^3J$(C-F) = 1.3 Hz, C-1); 94.98 (d, $^1J$(C-F) = 169.4 Hz, C-5); 121.21 (C-14); 123.15 (C-8); 127.40 (C-12, C-16); 131.38 (C-13, C-15); 132.21 (C-9); 141.00 (C-11). HR-MS: 354.0620 ($M^+$ ($C_{17}H_{20}O_2FBr$)$^+$; calc. 354.0625).

**Reaction of 1 with cyclohexanecarbaldehyde (6k):** The reaction of diol **1** and aldehyde **6k** for 8 hours at 2 °C gave products **2k** (*R*:*S* = 1:3, 27%), **8k** (*R*:*S* = 3:1, 61%).

**(2*S*,4*S*,4a*R*,8*R*,8a*R*)-2-cyclohexyl-4,7-dimethyl-3,4,4a,5,8,8a-hexahydro-2*H*-chromene-4,8-diol ((*S*)-2k):** Transparent oil. $^1$H NMR (CDCl$_3$+CD$_3$OD): δ = 0.74-0.87 (m, 2 H, 12-H$_a$, 16-H$_a$); 0.93-1.10

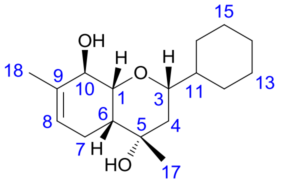


(m, 3 H, 13-H, 14-H, 15-H); 1.15-1.25 (m, 1 H, 11-H); 1.24 (d, $J$(17,4a) = 0.6 Hz, 3 H, 17-H); 1.29 (ddd, $J$(4e,4a) = 13.2 Hz, $J$(4e,3a) = 2.6 Hz, $J$(4e,6a) = 1.0 Hz, 1 H, 4-H$_e$); 1.44 (dd, $J$(4a,4e) = 13.2 Hz, $J$(4a,3a) = 11.8 Hz, 1 H, 4-H$_a$); 1.46-1.59 (m, 4 H, 12-H$_e$ or 16-H$_e$, 13-H’, 14-H’, 15-H’); 1.58 (br.dd, $J$(6a,7a) = 10.8 Hz, $J$(6a,7e) = 6.6 Hz, 1 H, 6-H$_a$); 1.65 (m, all $J$ ≤ 2.5 Hz, 3 H, 18-H); 1.74-1.81 (m, 1 H, 16-H$_e$ or 12-H$_e$); 1.86 (dddq, $J$(7a,7e) = 18.0 Hz, $J$(7a,6a) = 10.8 Hz, $J$(7a,8) = 2.5 Hz, $J$(7a,18) = 2.5 Hz, 1 H, 7-H$_a$); 1.94 (dddq, $J$(7e,7a) = 18.0 Hz, $J$(7e,6a) = 6.6 Hz, $J$(7e,8) = 5.2 Hz, $J$(7e,18) = 1.6 Hz, 1 H, 7-H$_e$); 2.95 (ddd, $J$(3a,4a) = 11.8 Hz, $J$(3a,11) = 6.7 Hz, $J$(3a,4e) = 2.6 Hz, 1 H, 3-H$_a$); 3.41 (dd, $J$(1e,10e) = 2.3 Hz, $J$(1e,6a) = 2.1 Hz, 1 H, 1-H$_e$); 3.63 (br.s, 1 H, 10-H$_e$); 5.44-5.47 (m, 1 H, 8-H). $^{13}$C NMR (CDCl$_3$+CD$_3$OD) δ = 20.32 (C-18); 22.36 (C-7); 25.62, 25.77, 26.18 (C-13, C-14, C-15); 26.54 (C-17); 28.06, 28.91 (C-12, C-16); 37.29 (C-4); 38.40 (C-6); 42.27 (C-11); 70.02 (C-10); 70.53 (C-5); 77.32 (C-1); 79.66 (C-3); 124.30 (C-8); 131.02 (C-9). HR-MS: 262.1923 ([$M$-$H_2O$]$^+$ ($C_{17}H_{26}O_2$)$^+$; calc. 262.1927).

**(2*S*,4*R*,4a*R*,8*R*,8a*R*)-2-cyclohexyl-4,7-dimethyl-3,4,4a,5,8,8a-hexahydro-2*H*-chromene-4,8-diol ((*R*)-2k):** Transparent oil. $^1$H NMR (CDCl$_3$+CD$_3$OD): δ = 0.74-0.87 (m, 2 H, 12-H$_a$, 16-H$_a$); 0.94-1.11

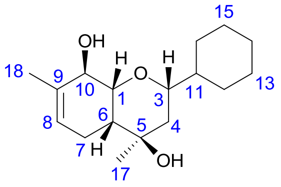


(m, 3 H, 13-H, 14-H, 15-H); 1.04 (s, 3 H, 17-H); 1.15-1.23 (m, 1 H, 11-H); 1.25-1.31 (m, 2 H, 4-H); 1.44-1.59 (m, 5 H, 6-H$_a$, 12-H$_e$ or 16-H$_e$, 13-H’, 14-H’, 15-H’); 1.65 (m, all $J$ ≤ 2.5 Hz, 3 H, 18-H); 1.74-1.79 (m, 2 H, 7-H); 3.25-3.30 (m, 1 H, 3-H$_a$); 3.65 (br.s, 1 H, 10-H$_e$); 3.81 (dd, $J$(1e,10e) = 2.4 Hz, $J$(1e,6a) = 2.2 Hz, 1 H, 1-H$_e$); 5.38-5.41 (m, 1 H, 8-H). $^{13}$C NMR (CDCl$_3$+CD$_3$OD) δ = 20.39 (C-18); 24.14 (C-7); 25.65, 25.79, 26.21 (C-13, C-14, C-15); 27.81 (C-17); 27.93, 28.94 (C-12, C-16);

36.36 (C-4); 37.83 (C-6); 42.14 (C-11); 70.02 (C-10); 70.53 (C-5); 74.85 (C-1); 77.87 (C-3); 123.66 (C-8); 131.61 (C-9).

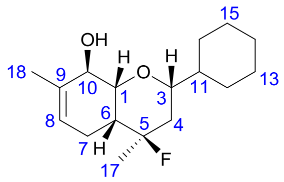


**(2*S*,4*R*,4a*R*,8*R*,8a*R*)-2-cyclohexyl-4-fluoro-4,7-dimethyl-3,4,4a,5,8,8a-hexahydro-2*H*-chromen-8-ol ((*R*)-8k):** Yellow oil. $^{1}$H NMR (CDCl$_3$): δ = 0.85-0.98 (m, 2 H, 12-H$_a$, 16-H$_a$); 1.04-1.23 (m, 3 H, 13-H, 14-H, 15-H); 1.29 (d, $^{3}J$(17,F) = 22.3 Hz, 3 H, 17-H); 1.24-1.34 (m, 1 H, 11-H); 1.45 (ddd, $^{3}J$(4a,F) = 40.7 Hz, *J*(4a,4e) = 14.3 Hz, *J*(4a,3a) = 11.7 Hz, 1 H, 4-H$_a$); 1.55-1.71 (m, 5 H, 4-H$_e$, 13-H', 14-H', 15-H', 16-H$_e$ or 12-H$_e$); 1.78 (m, all *J* ≤ 2.5 Hz, 3 H, 18-H); 1.72-1.80 (m, 1 H, 7-H); 1.82-1.95 (m, 3 H, 6-H, 7-H', 12-H$_e$ or 16-H$_e$); 3.36 (ddd, *J*(3a,4a) = 11.7 Hz, *J*(3a,11) = 7.1 Hz, *J*(3a,4e) = 2.6 Hz, 1 H, 3-H$_a$); 3.80 (br.s, 1 H, 10-H$_e$); 3.86 (m, all *J* ≤ 3.0 Hz, 1 H, 1-H$_e$); 5.48-5.52 (m, 1 H, 8-H). $^{13}$C NMR (CDCl$_3$) δ = 20.80 (C-18); 23.91 (d, $^{3}J$(C-F) = 9.6 Hz, C-7); 25.01 (d, $^{2}J$(C-F) = 24.5 Hz, C-17); 25.73, 25.92, 26.36 (C-13, C-14, C-15); 28.11, 28.92 (C-12, C-16); 35.33 (d, $^{2}J$(C-F) = 21.6 Hz, C-4); 36.40 (d, $^{2}J$(C-F) = 21.0 Hz, C-6); 42.19 (C-11); 70.09 (C-10); 74.97 (C-1); 77.85 (C-3); 95.19 (d, $^{1}J$(C-F) = 167.8 Hz, C-5); 123.24 (C-8); 132.17 (C-9). HR-MS: 263.2005 ([*M-F*]$^{+}$ ($C_{17}H_{27}O_2$)$^{+}$; calc. 263.2006).

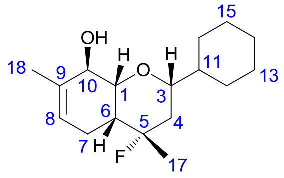


**(2*S*,4*S*,4a*R*,8*R*,8a*R*)-2-cyclohexyl-4-fluoro-4,7-dimethyl-3,4,4a,5,8,8a-hexahydro-2*H*-chromen-8-ol ((*S*)-8k):** Yellow oil. $^{1}$H NMR (CDCl$_3$): δ = 1.49 (d, $^{3}J$(17, F) = 23.7 Hz, 3 H, 17-H); 1.78 (m, all *J* ≤ 2.5 Hz, 3 H, 18-H); 2.02-2.08 (m, 2 H, 7-H); 3.00 (ddd, *J*(3a,4a) = 12.0 Hz, *J*(3a,11) = 6.7 Hz, *J*(3a,4e) = 2.4 Hz, 1 H, 3-H$_a$); 3.44 (m, all *J* ≤ 3.0 Hz, 1 H, 1-H$_e$); 3.78 (m, 1 H, 10-H$_e$); 5.55-5.59 (m, 1 H, 8-H). The signals of the others protons are overlapped by those of the main isomer (*R*)-7k in the areas 0.86-0.98, 1.05-1.23, 1.28-1.36, 1.56-1.71 and 1.87-1.95 ppm. $^{13}$C NMR (CDCl$_3$) δ = 20.67 (C-18); 22.36 (d, $^{3}J$(C-F) = 6.2 Hz, C-7); 24.93 (d, $^{2}J$(C-F) = 25.9 Hz, C-17); 25.75, 25.91, 26.32 (C-13, C-14, C-15); 28.28, 28.94 (C-12, C-16); 36.01 (d, $^{2}J$(C-F) = 20.0 Hz, C-4); 38.01 (d, $^{2}J$(C-F) = 18.5 Hz, C-6); 42.35 (C-11); 70.09 (C-10); 77.43 (d, $^{3}J$(C-F) = 7.3 Hz, C-1); 79.92 (d, $^{3}J$(C-F) = 10.7 Hz, C-3); 95.67 (d, $^{1}J$(C-F) = 175.2 Hz, C-5); 124.39 (C-8); 131.22 (C-9).

**Reaction of 1 with (*E*)-but-2-enal 6l:** The reaction of diol **1** and aldehyde **6l** for 8 hours at 2°C gave products **2l** (*R*:*S* = 1:2, 20%), **8l** (*R*:*S* = 3:1, 57%). Spectral characteristics of compounds **2l** have coincided with those described in the work [1].

**(2*S*,4*R*,4a*R*,8*R*,8a*R*)-4-fluoro-4,7-dimethyl-2-((*E*)-prop-1-en-1-yl)-3,4,4a,5,8,8a-hexahydro-2*H*-chromen-8-ol ((*R*)-8l):** Yellow oil. $^{1}$H NMR (CDCl$_3$): δ = 1.29 (d, $^{3}J$(14, F) = 22.3 Hz, 3 H, 14-H);

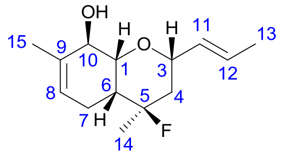


1.58 (ddd, $^3J$(4a,F) = 39.7 Hz, $J$(4a,4e) = 14.6 Hz, $J$(4a,3a) = 11.4 Hz, 1 H, 4-H$_a$); 1.64 (dd, $J$(13,12) = 6.5 Hz, $J$(13,11) = 1.7 Hz, 3 H, 13-H); 1.60-1.70 (m, 1 H, 4-H$_e$); 1.77 (m, all $J \leq$ 2.5 Hz, 3 H, 15-H); 1.76-1.84 (m, 1 H, 7-H$_a$); 1.86-1.96 (m, 2 H, 6-H$_a$, 7-H$_e$); 3.85 (m, 1 H, 10-H$_e$); 3.96 (m, all $J \leq$ 3.0 Hz, 1 H, 1-H$_e$); 4.12 (ddd, $J$(3a,4a) = 11.2 Hz, $J$(3a,11) = 7.0 Hz, $J$(3a,4e) = 3.1 Hz, 1 H, 3-H$_a$); 5.42 (ddq, $J$(11,12) = 15.4 Hz, $J$(11,3a) = 7.0 Hz, $J$(11,13) = 1.7 Hz, 1 H, 11-H); 5.48-5.52 (m, 1 H, 8-H); 5.68 (dqd, $J$(12,11) = 15.4 Hz, $J$(12,13) = 6.5 Hz, $J$(12,3a) = 1.0 Hz, 1 H, 12-H). $^{13}$C NMR ($CDCl_3$) δ = 17.57 (C-13); 20.75 (C-15); 23.89 (d, $^3J$(C-F) = 9.6 Hz, C-7); 24.71 (d, $^2J$(C-F) = 24.5 Hz, C-14); 35.95 (d, $^2J$(C-F) = 20.9 Hz, C-6); 38.26 (d, $^2J$(C-F) = 21.8 Hz, C-4); 69.94 (C-10); 74.48 (C-3); 74.79 (C-1); 94.85 (d, $^1J$(C-F) = 168.1 Hz, C-5); 123.22 (C-8); 127.99 (C-12); 131.08 (C-11); 132.02 (C-9). HR-MS: 240.1524 ($M^+$ $(C_{14}H_{21}O_2F)^+$; calc. 240.1520).

**(2*S*,4*S*,4a*R*,8*R*,8a*R*)-4-fluoro-4,7-dimethyl-2-((*E*)-prop-1-en-1-yl)-3,4,4a,5,8,8a-hexahydro-2*H*-chromen-8-ol ((*S*)-8l):** Yellow oil. $^1$H NMR ($CDCl_3$): δ = 1.53 (dd, $^3J$(14, F) = 22.3 Hz, $J$(14, 4a) = 0.8 Hz, 3 H, 14-H); 1.59-1.70 (m, 5 H, 4-2H, 13-3H); 1.77 (m, 3 H, 15-H); 1.93-1.98 (m, 1 H, 6-H$_a$); 2.05-2.12 (m, 2 H, 7-H); 3.54 (m, all $J \leq$ 3.0 Hz, 1 H, 1-H$_e$); 3.76 (br.ddd, $J$(3a,4a) = 12.0 Hz, $J$(3a,11) = 6.7 Hz, $J$(3a,4e) = 2.5 Hz, 1 H, 3-H$_a$); 3.83 (m, 1 H, 10-H$_e$); 5.45 (ddq, $J$(11,12) = 15.3 Hz, $J$(11,3a) = 6.7 Hz, $J$(11,13) = 1.6 Hz, 1 H, 11-H); 5.56-5.60 (m, 1 H, 8-H); 5.64-5.72 (m, 1 H, 12-H). $^{13}$C NMR ($CDCl_3$) δ = 17.51 (C-13); 20.62 (C-15); 22.35 (d, $^3J$(C-F) = 6.2 Hz, C-7); 24.74 (d, $^2J$(C-F) = 25.9 Hz, C-14); 37.57 (d, $^2J$(C-F) = 18.7 Hz, C-6); 38.95 (d, $^2J$(C-F) = 20.1 Hz, C-4); 69.97 (C-10); 76.33 (d, $^3J$(C-F) = 11.6 Hz, C-3); 77.36 (d, $^3J$(C-F) = 7.5 Hz, C-1); 94.91 (d, $^1J$(C-F) = 175.9 Hz, C-5); 124.40 (C-8); 128.22 (C-12); 130.68 (C-11); 131.08 (C-9).

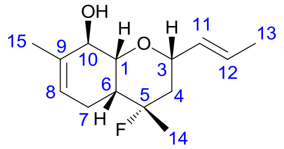


**Reaction of 1 with 4-hydroxy-3-methoxybenzaldehyde (6m):** The reaction of diol **1** and aldehyde **6m** for 8 hours at 2 °C gave products **2m** (*R*:*S* = 1:3, 35%), **8m** (*R*:*S* = 3:1, 60%). Spectral characteristics of compounds **2m** have coincided with those described in the work [2].

**(2*S*,4*R*,4a*R*,8*R*,8a*R*)-4-fluoro-2-(4-hydroxy-3-methoxyphenyl)-4,7-dimethyl-3,4,4a,5,8,8a-hexahydro-2*H*-chromen-8-ol ((*R*)-8m):** Yellow viscous oil. $^1$H NMR ($CDCl_3$): δ = 1.36 (d, $^3J$(17, F) = 22.4 Hz, 3 H, 17-H); 1.81 (ddd, $^3J$(4a, F) = 39.2 Hz, $J$(4a,4e) = 14.6 Hz, $J$(4a, 3a) = 11.5 Hz, 1 H, 4-H$_a$); 1.82 (m, all $J \leq$ 2.5 Hz, 3 H, 18-H); 1.85-1.92 (m, 1 H, 4-H$_e$); 1.92-2.06 (m, 3 H, 6-H$_a$, 7-2H); 3.86 (s, 3 H, 19-MeO); 3.94 (br.s, 1 H, 10-H$_e$); 4.15 (m, all $J$ < 3.0 Hz, 1 H, 1-H$_e$); 4.67 (dd, $J$(3a,4a) = 11.5 Hz, $J$(3a,4e) = 3.3 Hz, 1 H, 3-H$_a$); 5.56 (br.s, 1 H, 14-HO); 5.56-5.60 (m, 1 H, 8-H); 6.79-6.82

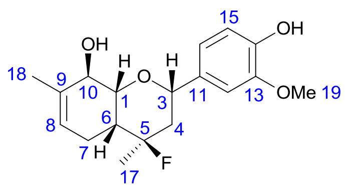


(m, 2 H, 12-H, 16-H); 6.85 (d, $J$(15,16) = 8.5 Hz, 1 H, 15-H). $^{13}$C NMR (CDCl$_3$) δ = 20.77 (C-18); 24.10 (d, $^3J$(C-F) = 9.7 Hz, C-7); 24.81 (d, $^2J$(C-F) = 24.5 Hz, C-17); 36.14 (d, $^2J$(C-F) = 20.9 Hz, C-6); 40.17 (d, $^2J$(C-F) = 21.6 Hz, C-4); 55.84 (C-19); 70.20 (C-10); 75.47 (d, $^3J$(C-F) = 1.0 Hz, C-1); 75.72 (C-3); 95.21 (d, $^1J$(C-F)=168.8 Hz, C-5); 108.82 (C-12); 114.21 (C-15); 118.79 (C-16); 123.22 (C-8); 132.23 (C-9); 133.81 (C-11); 145.10 (C-14); 146.34 (C-13). HR-MS: 322.1573 ($M^+$ $(C_{18}H_{23}O_4F)^+$; calc. 322.1575).

**(2*S*,4*S*,4a*R*,8*R*,8a*R*)-4-fluoro-2-(4-hydroxy-3-methoxyphenyl)-4,7-dimethyl-3,4,4a,5,8,8a-hexahydro-2*H*-chromen-8-ol ((*S*)-8m):** Yellow viscous oil. $^1$H NMR (CDCl$_3$): δ = 1.64 (dd, $^3J$(17, F) = 23.5 Hz, $J$(17, 4a) = 0.8 Hz, 3 H, 17-H); 1.81 (m, all $J \leq 2.5$ Hz, 3 H, 18-H); 1.83 (br.dd, $J$(4e, 4a) = 13.3 Hz, $J$(4e,3a) = 2.6 Hz, 1 H, 4-H$_e$); 2.05 (br.dd, $J$(6a,7a) = 10.6 Hz, $J$(6a,7e) = 6.5, 1 H, 6-H$_a$); 2.17 (dddq, $^3J$(4a, F) ~ 14.0 Hz, $J$(4a,4e) = 13.3 Hz, $J$(4a,3a) = 12.1 Hz, $J$(4a,17) = 0.8 Hz, 1 H, 4-H$_a$); 2.15-2.28 (m, 2 H, 7-H); 3.72 (m, all $J \leq 2.5$ Hz, 1 H, 1-H$_e$); 3.87 (s, 3 H, 19-MeO); 3.92 (br.s, 1 H, 10-H$_e$); 4.30 (dd, $J$(3a,4a) = 12.1 Hz, $J$(3a,4e) = 2.6 Hz, 1 H, 3-H$_a$); 5.58 (br.s, 1 H, 14-HO); 5.63-5.66 (m, 1 H, 8-H); 6.79-8.82 (m, 2 H, 12-H, 16-H); 6.85 (d, $J$(15,16) = 8.5 Hz, 1 H, 15-H). $^{13}$C NMR (CDCl$_3$) δ = 20.64 (C-18); 22.54 (d, $^3J$(C-F) = 6.4 Hz, C-7); 24.84 (d, $^2J$(C-F) = 25.9 Hz, C-17); 37.80 (d, $^2J$(C-F) = 18.9 Hz, C-6); 40.62 (d, $^2J$(C-F) = 20.4 Hz, C-4); 55.85 (C-19); 70.23 (d, $^4J$(C-F) = 2.1 Hz, C-10); 77.65 (d, $^3J$(C-F) = 11.6 Hz, C-3); 77.93 (d, $^3J$(C-F) = 7.9 Hz, C-1); 95.18 (d, $^1J$(C-F)=176.2 Hz, C-5); 108.77 (C-12); 114.19 (C-15); 119.09 (C-16); 124.46 (C-8); 131.27 (C-9); 133.09 (C-11); 145.35 (C-14); 146.40 (C-13).

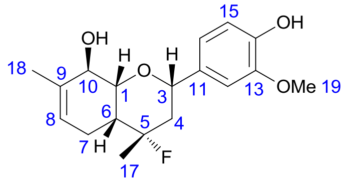


**Reaction of isopulegol 4 with 3,4,5-trimethoxybenzaldehyde (6a):** The reaction of isopulegol **4** and aldehyde **6a** for 8 hours at 2 °C gave products **11** (*R*:*S* = 7:2, 76%), **12** (*R*:*S* = 1:5, 15%). Spectral characteristics of compound **12** have coincided with those described in the work [5].

**(2*R*,4*R*,4a*R*,7*R*,8a*R*)-4-fluoro-4,7-dimethyl-2-(3,4,5-trimethoxyphenyl)octahydro-2*H*-chromene ((*R*)-11):** Yellow oil. $^1$H NMR (CDCl$_3$): δ = 0.85-1.00 (m, 1 H, 8-H$_a$); 0.93 (d, $J$(18, 9) = 6.6 Hz, 3 H, 18-H); 1.09 (ddd, $J$(10a,10e) = $J$(10a,9a) = 12.2 Hz, $J$(10a,1a) = 11.2 Hz, 1 H, 10-H$_a$); 1.18-1.34 (m, 2 H, 6-H$_a$, 7-H$_a$); 1.34 (d, $^3J$(17,F) = 21.4 Hz, 3 H, 17-H); 1.45-1.54 (m, 1 H, 9-H$_a$); 1.71 (ddd, $^3J$(4a,F) = 39.3 Hz, $J$(4a,4e) = 14.2 Hz, $J$(4a,3a) = 11.7 Hz, 1 H, 4-H$_a$); 1.70-1.78 (m, 1 H, 8-H$_e$); 1.89 (dm, $J$(7e,7a) = 13.1 Hz, 1 H, 7-H$_e$); 1.98-2.04 (m, 2 H, 4-H$_e$,

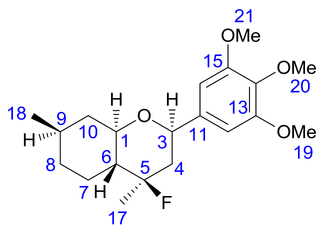

10-$H_e$); 3.56 (dddd, $J$(1a,10a) = 11.2 Hz, $J$(1a,6a) = 9.8 Hz, $J$(1a,10e) = 4.2 Hz, $^4J$(1a,F) = 1.5 Hz, 1 H, 1-$H_a$); 3.79 (s, 3 H, 20-MeO); 3.84 (s, 6 H, 19-MeO, 21-MeO); 4.68 (dd, $J$(3a,4a) = 11.7 Hz, $J$(3a,4e) = 2.3 Hz, 1 H, 3-$H_a$); 6.56 (s, 2 H, 12-H, 16-H). $^{13}$C NMR ($CDCl_3$) δ = 22.10 (C-18); 23.17 (C-7); 24.18 (d, $^2J$(C-F) = 24.8 Hz, C-17); 31.10 (C-9); 34.32 (C-8); 41.11 (C-10); 45.76 (d, $^2J$(C-F) = 21.7 Hz, C-4); 48.43 (d, $^2J$(C-F) = 20.3 Hz, C-6); 55.99 (C-19, C-21); 60.63 (C-20); 74.87 (C-3); 75.84 (C-1); 93.40 (d, $^1J$(C-F) = 171.7 Hz, C-5); 103.01 (C-12, C-16); 137.27 (C-14); 137.92 (C-11); 153.15 (C-13, C-15).

**(2*R*,4*S*,4a*R*,7*R*,8a*R*)-4-fluoro-4,7-dimethyl-2-(3,4,5-trimethoxyphenyl)octahydro-2*H*-chromene ((*S*)-11):** Yellow oil. $^1$H NMR ($CDCl_3$): δ = 0.85-1.00 (m, 1 H, 8-$H_a$); 0.94 (d, $J$(18, 9) = 6.5 Hz, 3 H, 18-H); 1.05-1.13 (m, 1 H, 7-$H_a$); 1.12-1.21 (m, 1 H, 10-$H_a$); 1.43 (d, $^3J$(17,F) = 23.1 Hz, 3 H, 17-H); 1.41-1.49 (m, 1 H, 9-$H_a$); 1.59 (dddd, $J$(6a,7a) = $J$(6a,F) = 12.2 Hz, $J$(6a,1a) = 10.2 Hz, $J$(6a,7e) = 3.3 Hz, 1 H, 6-$H_a$); 1.70-1.78 (m, 1 H, 8-$H_e$); 1.94 (dm, $J$(7e,7a) = 13.1 Hz, 1 H, 7-$H_e$); 1.96-2.08 (m, 3 H, 4-2H, 10-$H_e$); 3.21 (ddd, $J$(1a,10a) = 10.8 Hz, $J$(1a,6a) = 10.2 Hz, $J$(1a,10e) = 4.3 Hz, 1 H, 1-$H_a$); 3.79 (s, 3 H, 20-MeO); 3.85 (s, 6 H, 19-MeO, 21-MeO); 4.30 (dd, $J$(3a,4a) = 11.4 Hz, $J$(3a,4e) = 2.6 Hz, 1 H, 3-$H_a$); 6.55 (s, 2 H, 12-H, 16-H). $^{13}$C NMR ($CDCl_3$) δ = 19.44 (d, $^2J$(C-F) = 26.5 Hz, C-17); 22.04 (C-18); 22.40 (d, $^3J$(C-F) = 2.7 Hz, C-7); 31.26 (C-9); 33.92 (C-8); 41.23 (C-10); 47.13 (d, $^2J$(C-F) = 19.5 Hz, C-4); 49.88 (d, $^2J$(C-F) = 19.5 Hz, C-6); 56.02 (C-19, C-21); 60.63 (C-20); 76.91 (d, $^3J$(C-F) = 11.9 Hz, C-3); 77.54 (C-1); 94.88 (d, $^1J$(C-F) = 173.3 Hz, C-5); 103.24 (C-12, C-16); 137.12 (C-11); 137.54 (C-14); 153.17 (C-13, C-15). HR-MS: 352.2047 ($M^+$ $(C_{20}H_{29}O_4F)^+$; calc. 352.2044).

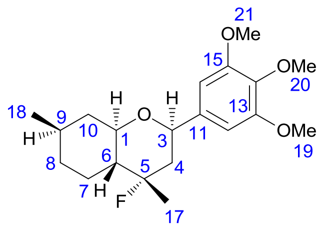

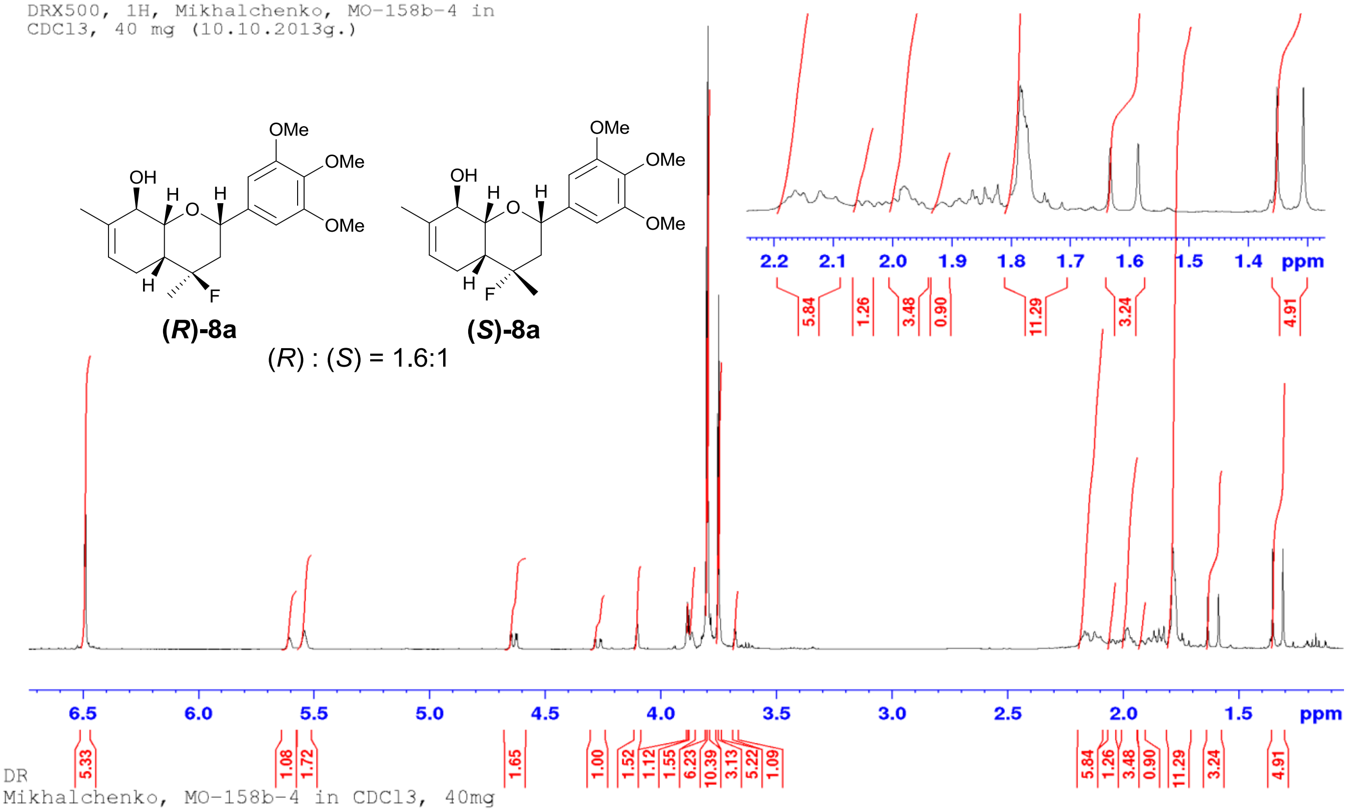
DRX500, 1H, Mikhalchenko, MO-158b-4 in CDCl3, 40 mg (10.10.2013g.)
(R)-8a
(S)-8a
(R) : (S) = 1.6:1
ppm

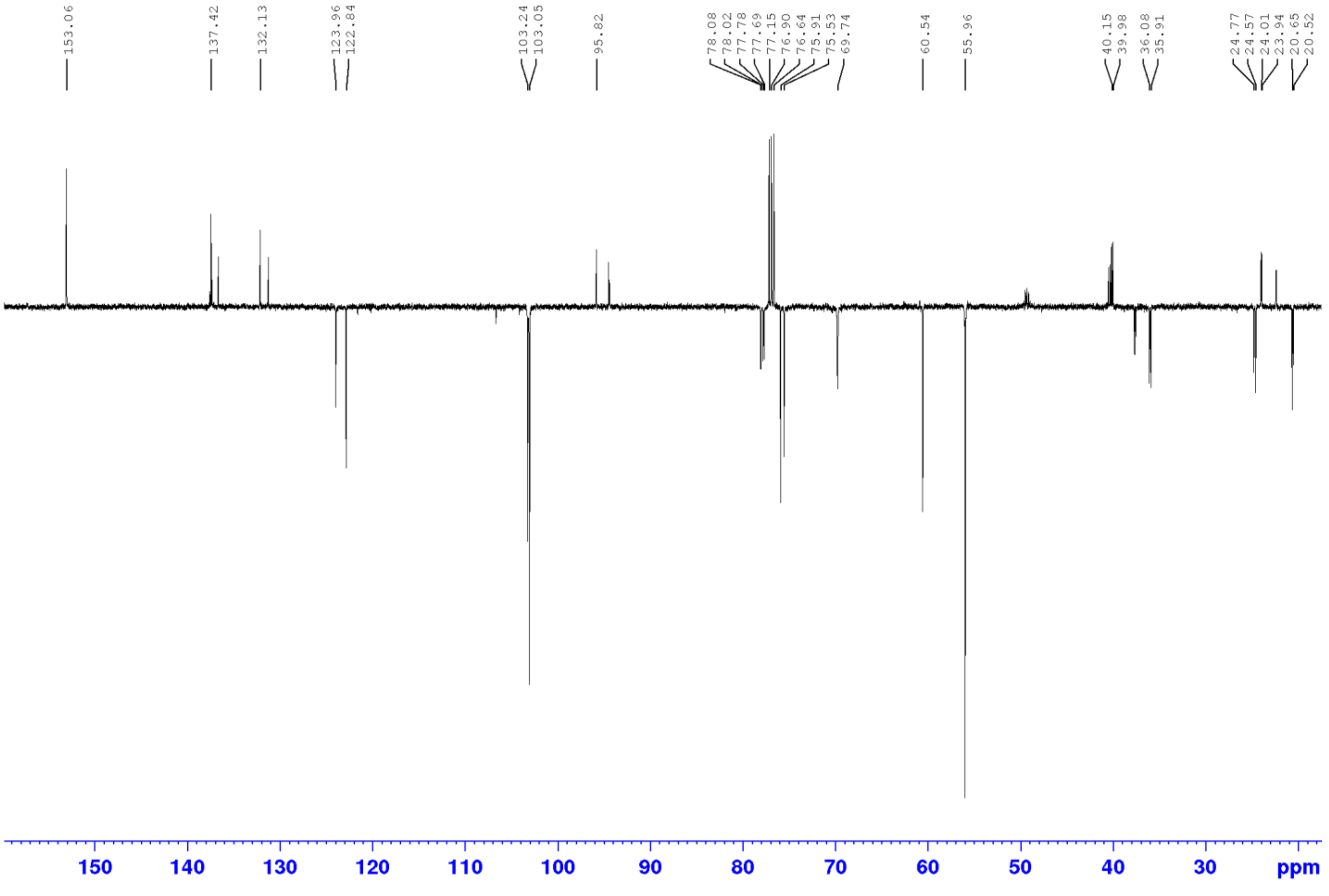
DR
Mikhalchenko, MO-158b-4 in CDCl3, 40mg
153.06
137.42
132.13
123.96
122.84
103.24
103.05
95.82
78.08
78.02
77.78
77.69
77.15
76.90
76.64
75.91
75.53
69.74
60.54
55.96
40.15
39.98
36.08
35.91
24.77
24.57
24.01
23.94
20.65
20.52
ppm

DRX500, 1H, Mikhalchenko, MO-150-1-3 in
CDCl3, 60mg (23.10.2013g.)

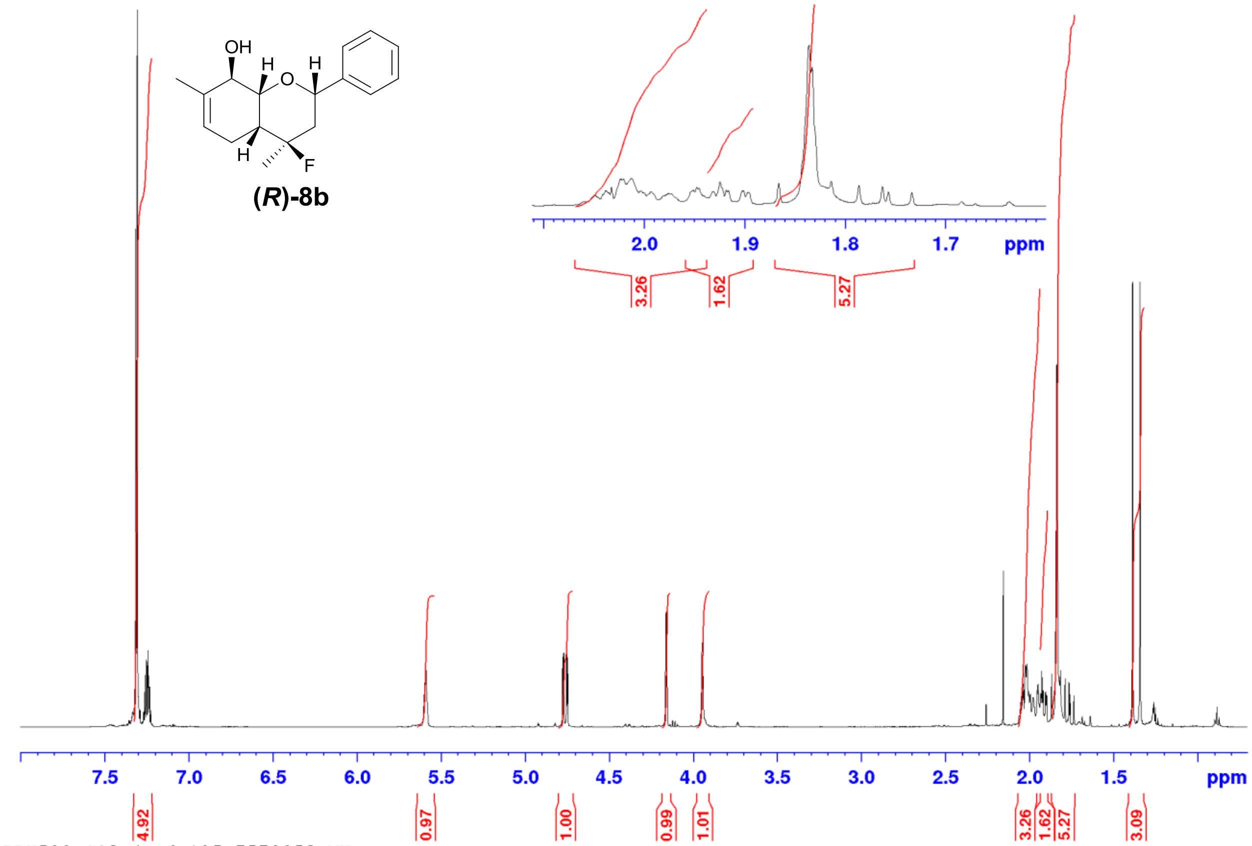


DRX500 C13 jmod 125.7578053 MHz,
Mikhalchenko, MO-150-1-3 in CDCl3, 60mg

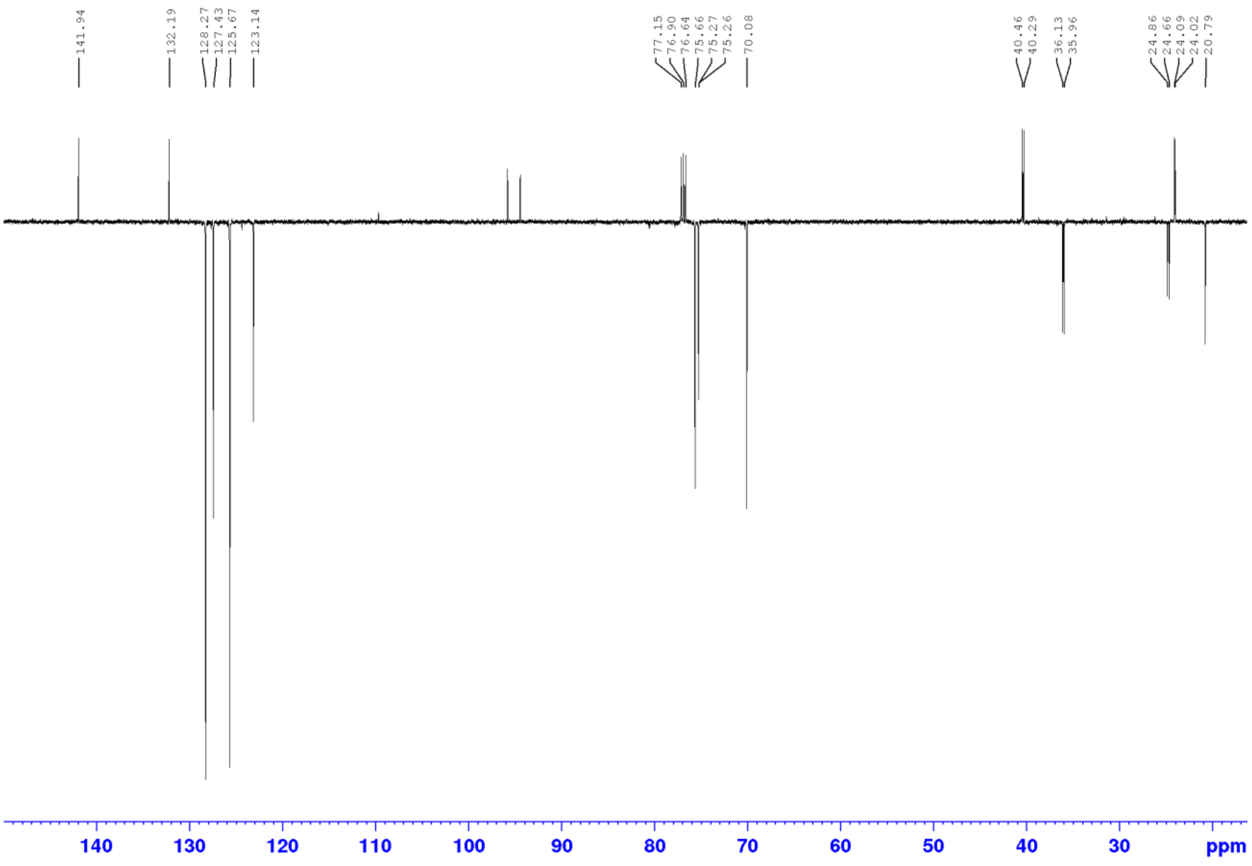

DRX500, 1H, Mikhalchenko, MO-150-1-5 in CDCl3, 17mg (23.10.2013g.)

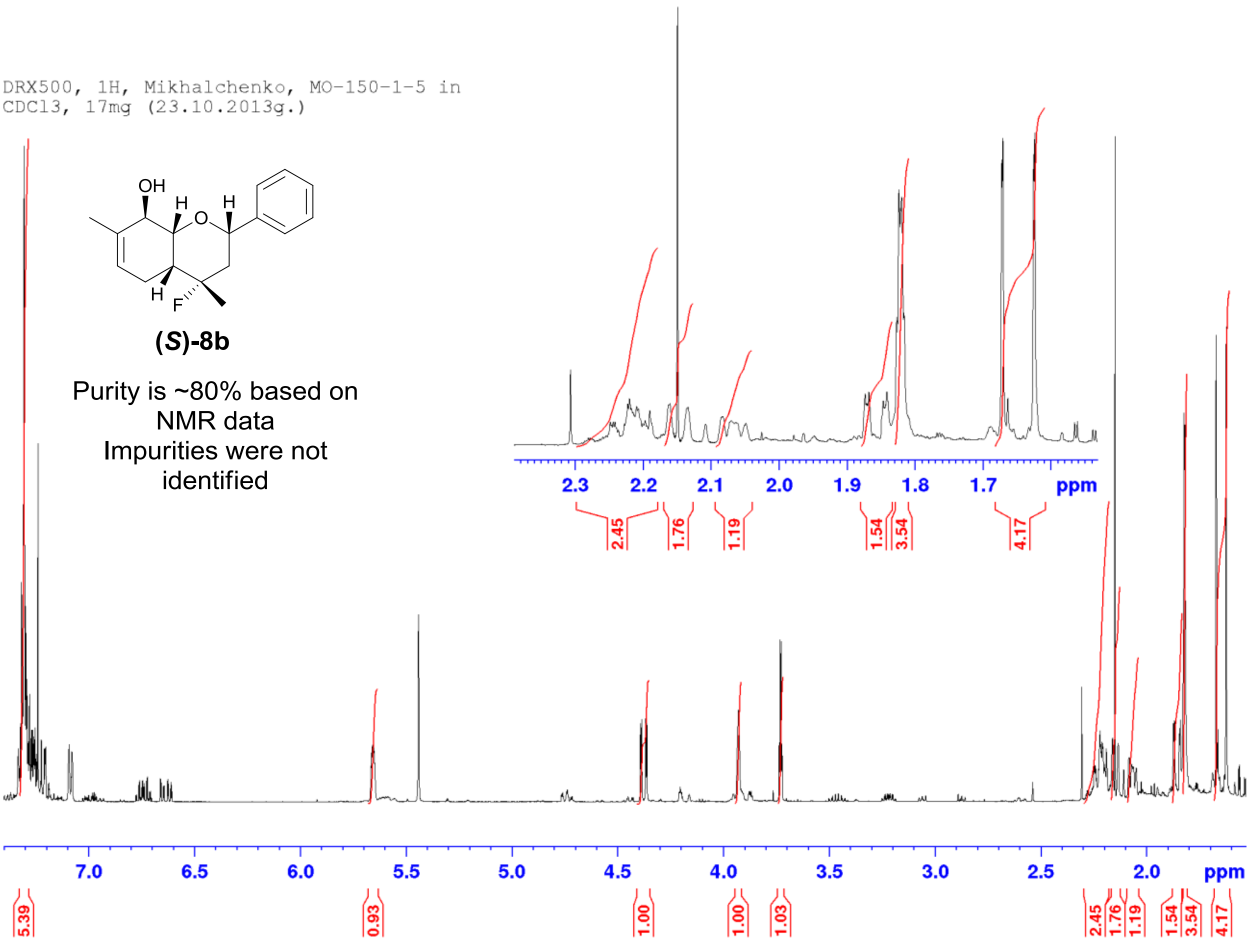

**(*S*)-8b**

Purity is ~80% based on NMR data
Impurities were not identified

DRX500 C13 jmod 125.7578053 MHz, Mikhalchenko, MO-150-1-5 in CDCl3, 17mg

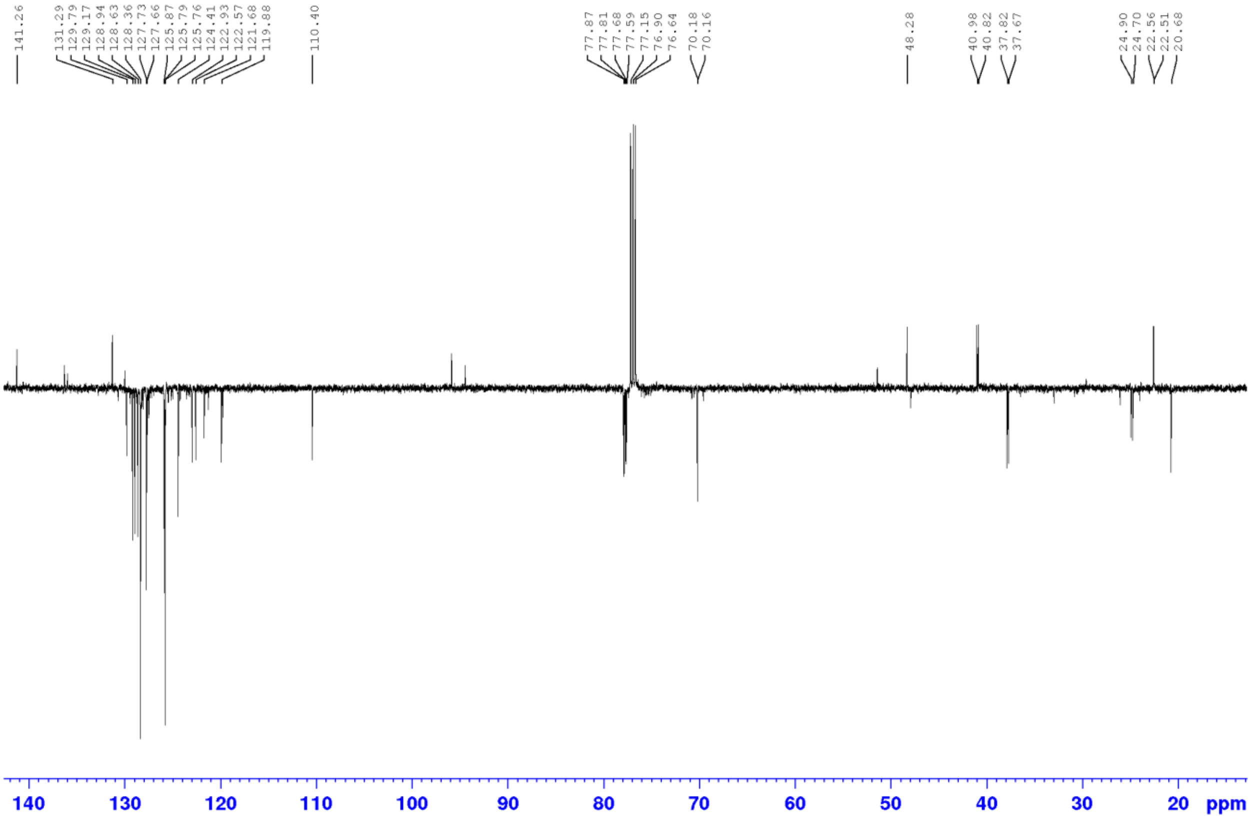

DRX500, 1H, Mikhalchenko, MO-151-2 in CDCl3, 30mg (09.10.2013g.)

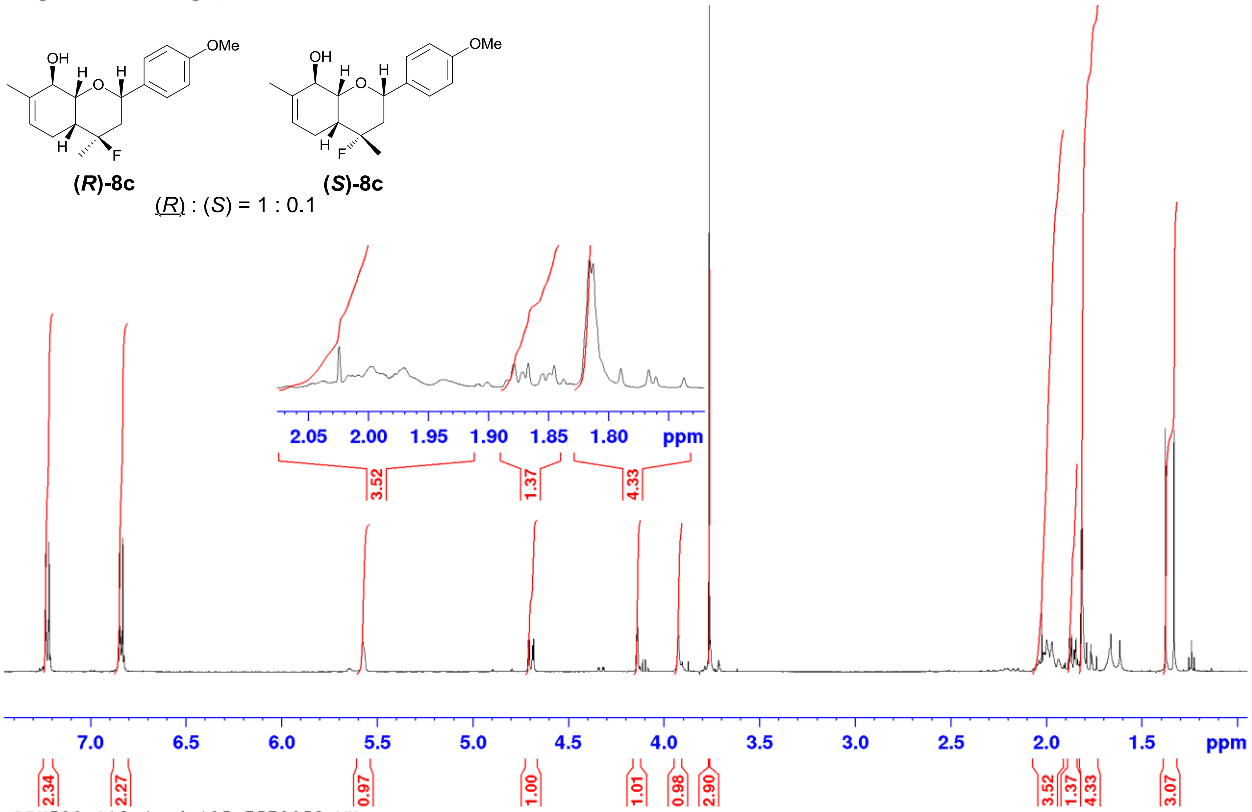


DRX500 C13 jmod 125.7578053 MHz, Mikhalchenko, MO-151-2 in CDCl3, 30mg

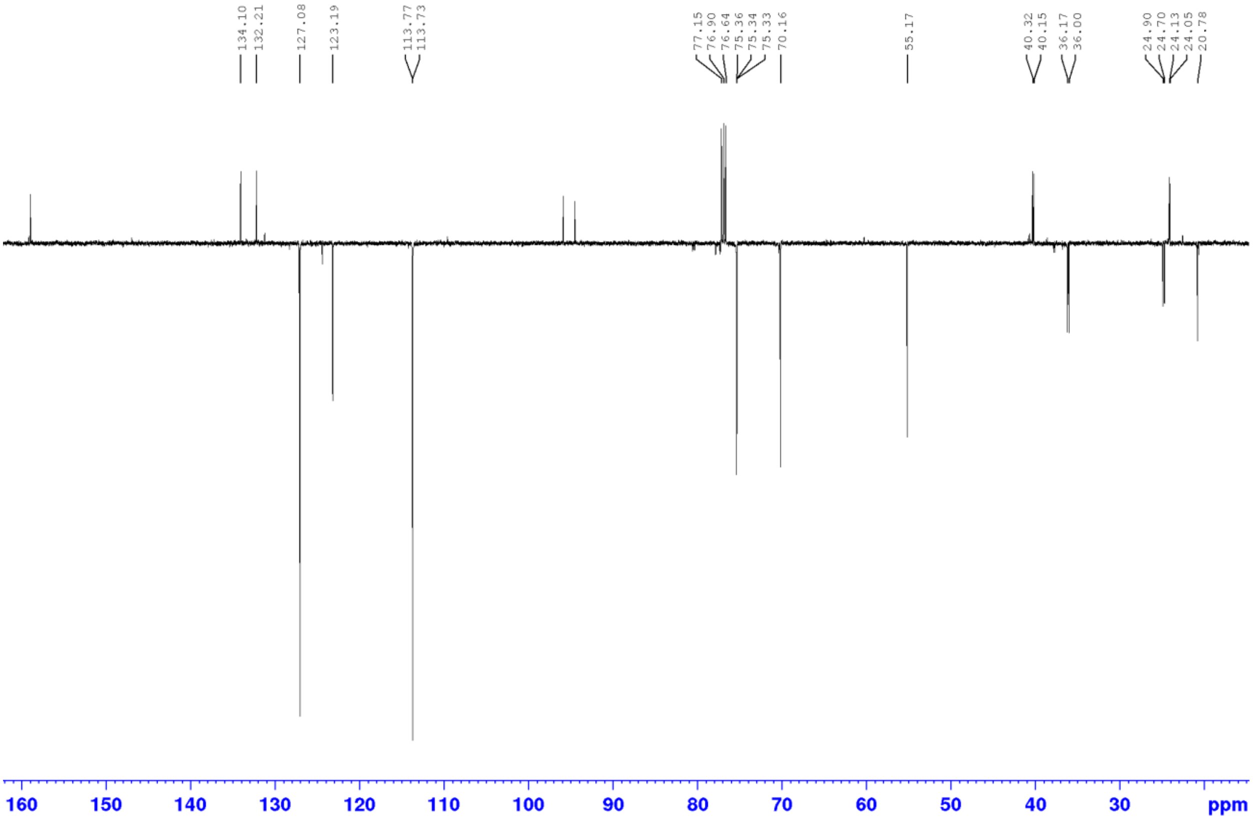

MO-151-3p; CDCl3

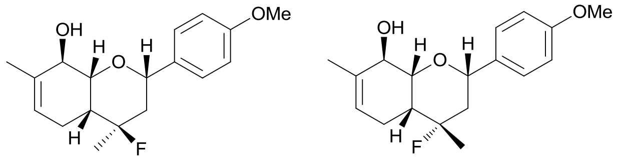


(*R*)-8c (**_S_**)-8c

(*R*) : **_(S)_** = 1 : 5

impurity is diethyl ether 4% by weight

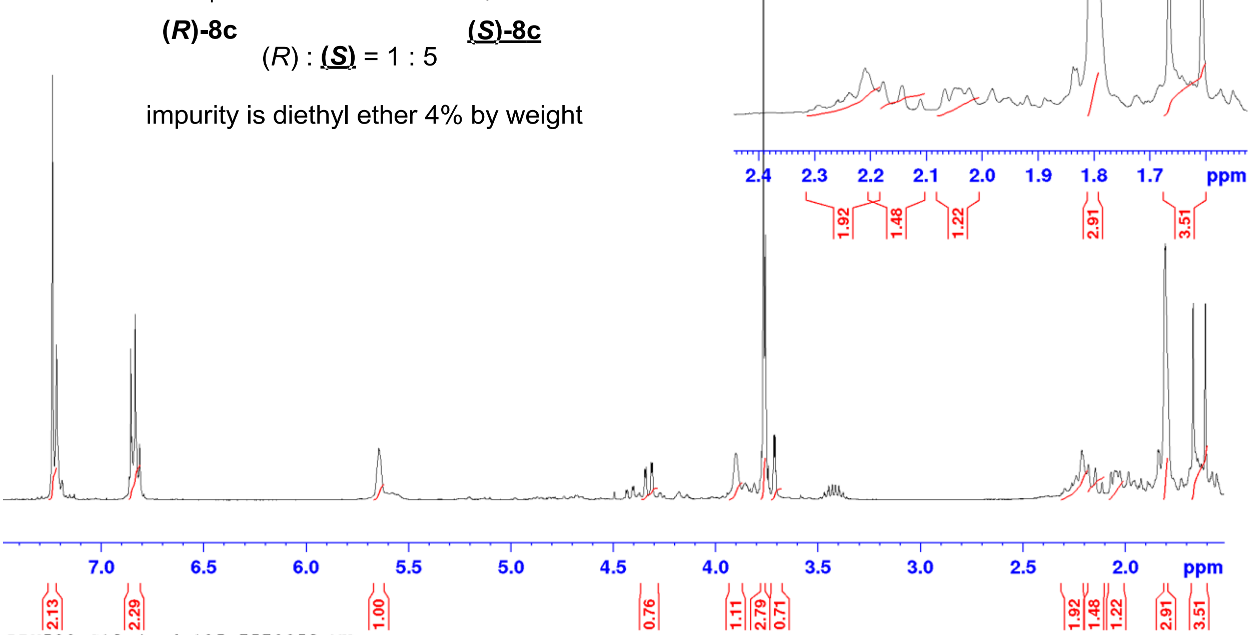


DRX500 C13 jmod 125.7578053 MHz,
Mikhalchenko, MO-151-3p in CDCl3, 40mg

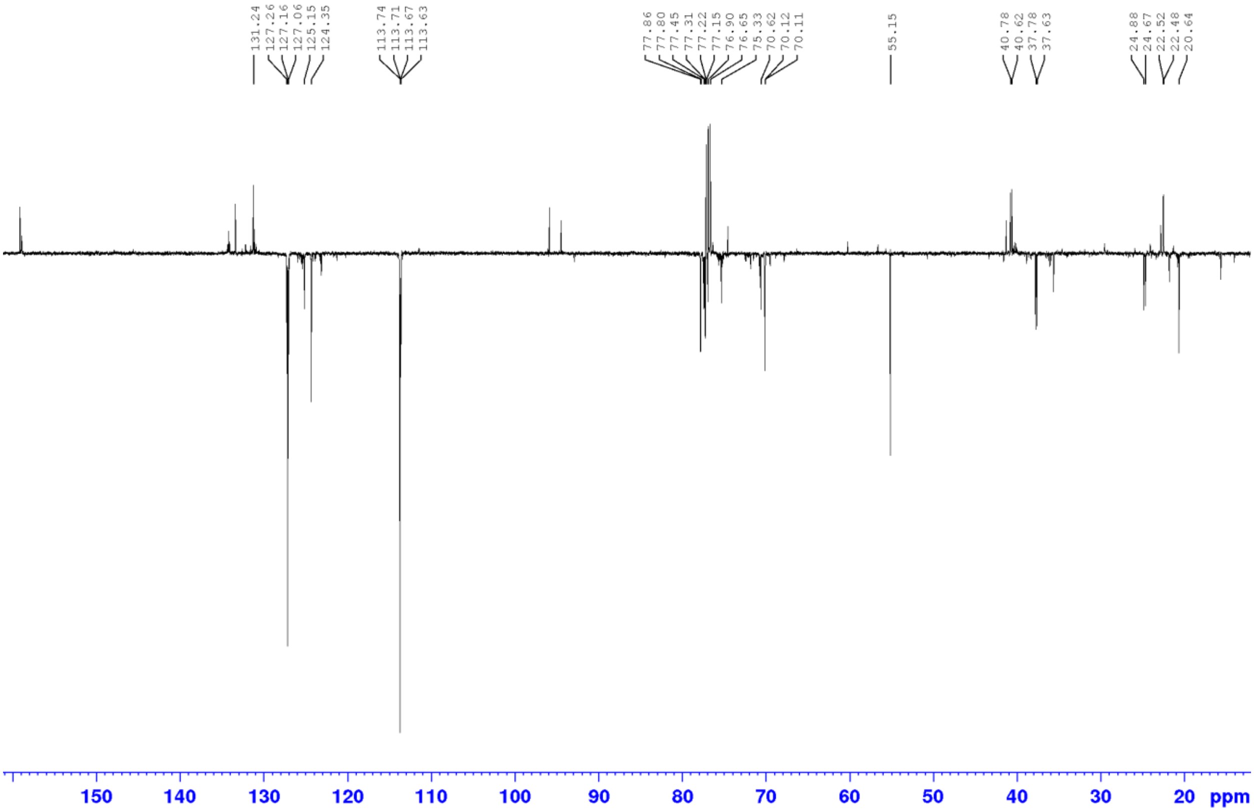

DRX500, 1H, Mikhalchenko, MO-152-2-2 in
CDCl3, 40mg (16.10.2013g.)

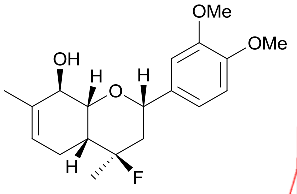


**(*R*)-8d**

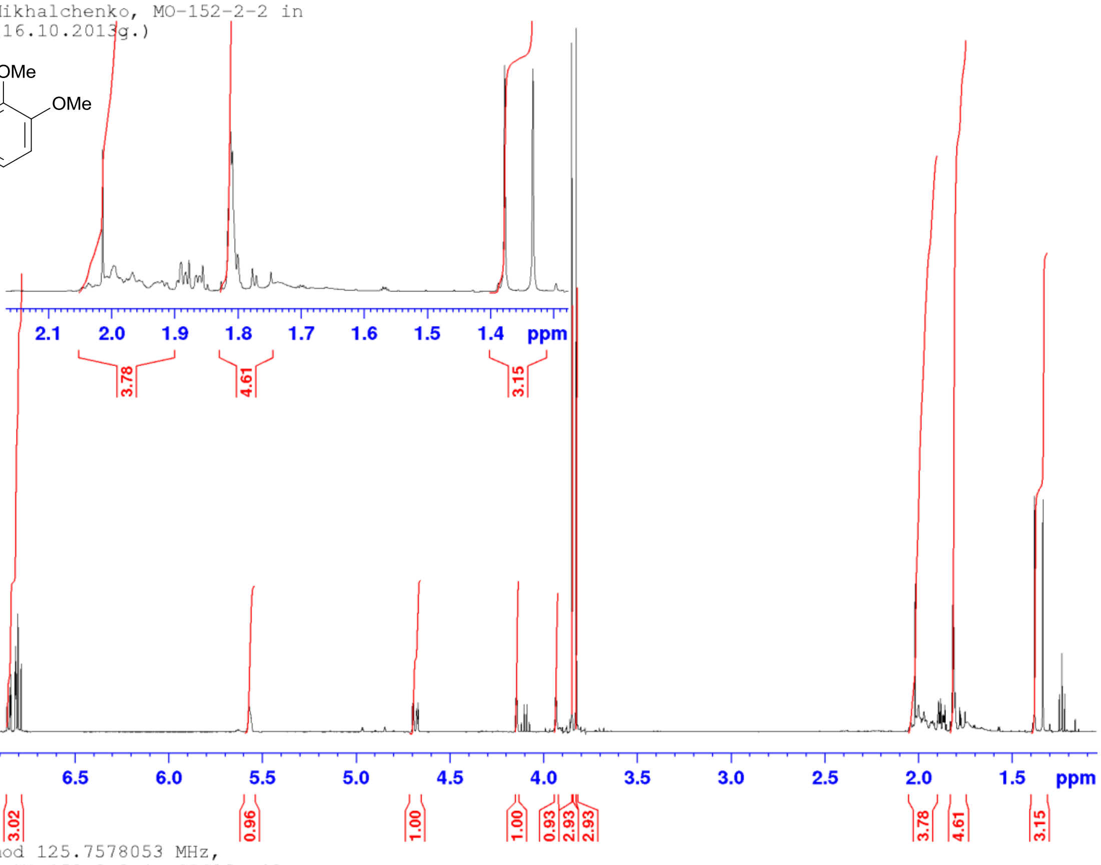


DRX500 C13 jmod 125.7578053 MHz,
Mikhalchenko, MO-152-2-2 in CDCl3, 40mg

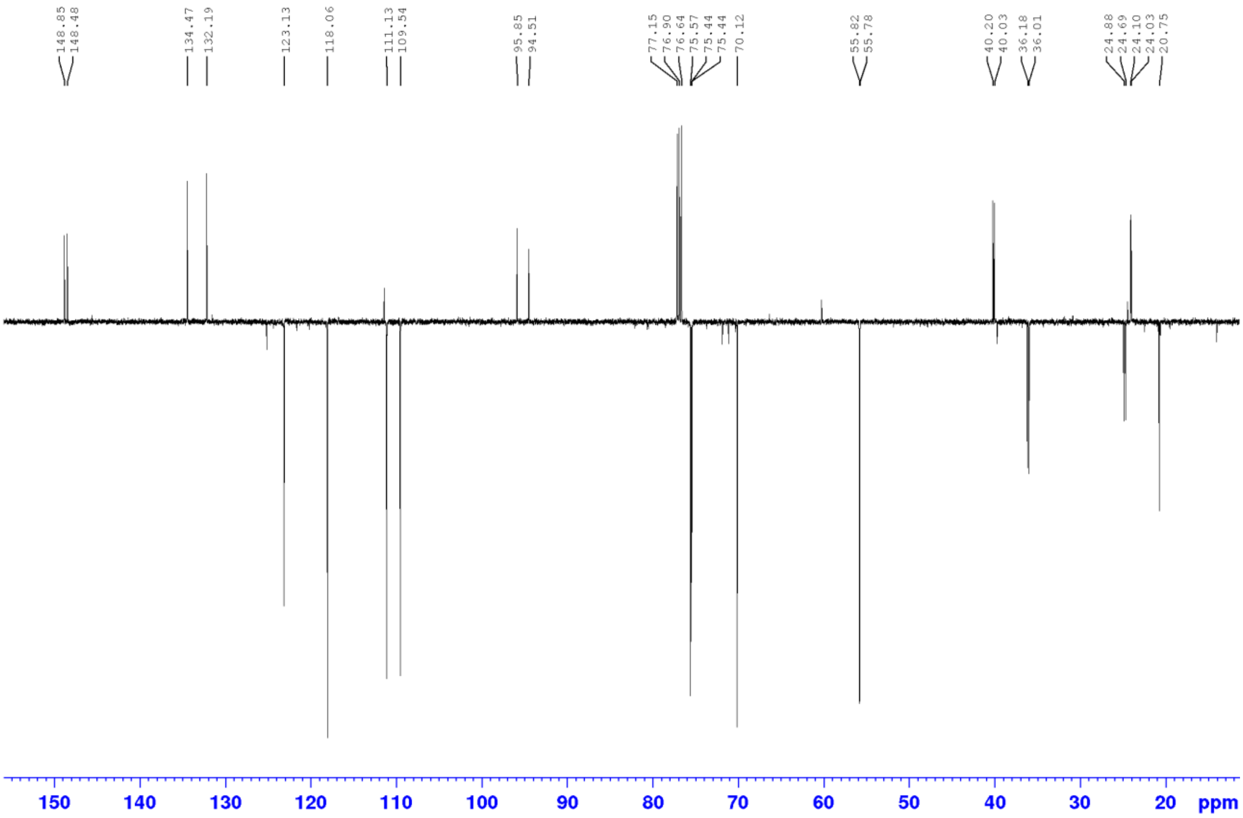

DRX500, 1H, Mikhalchenko, MO-152-2-3b in CDCl3, 30mg (16.10.2013g.)

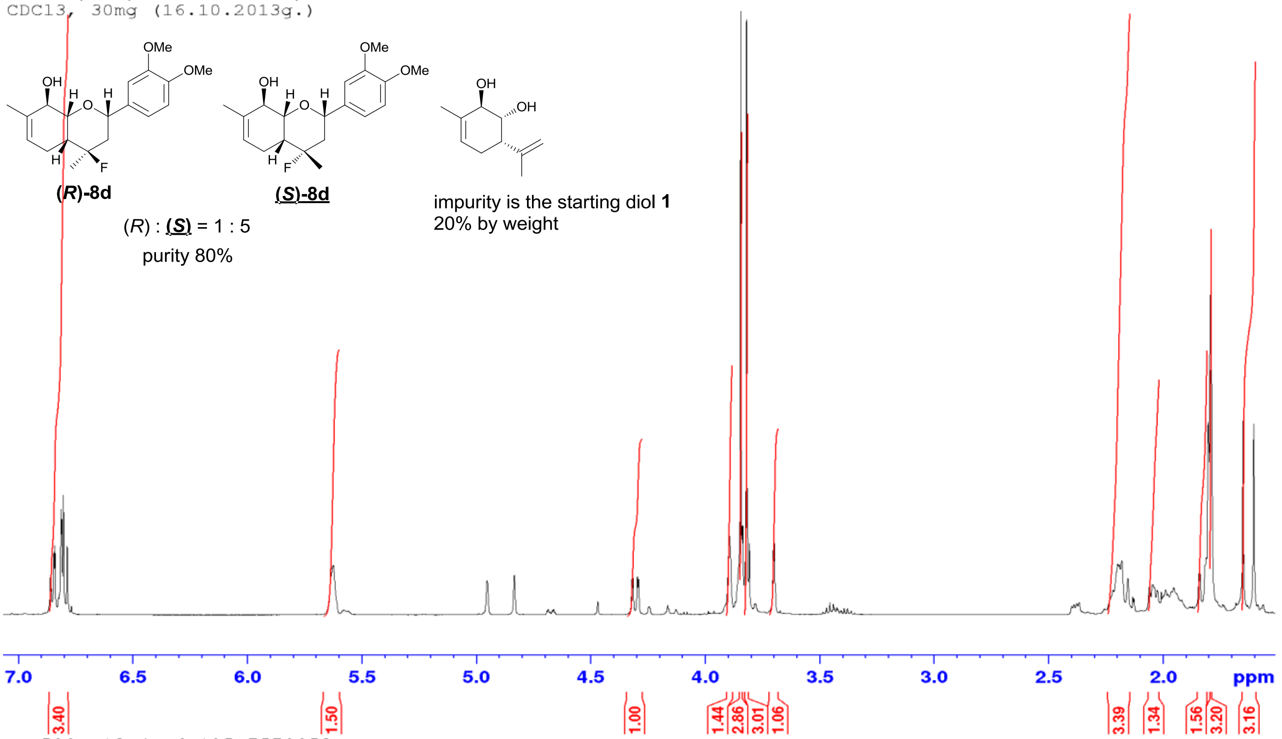


DRX500 C13 jmod 125.7578053 MHz, Mikhalchenko, MO-152-2-3b in CDCl3, 30mg

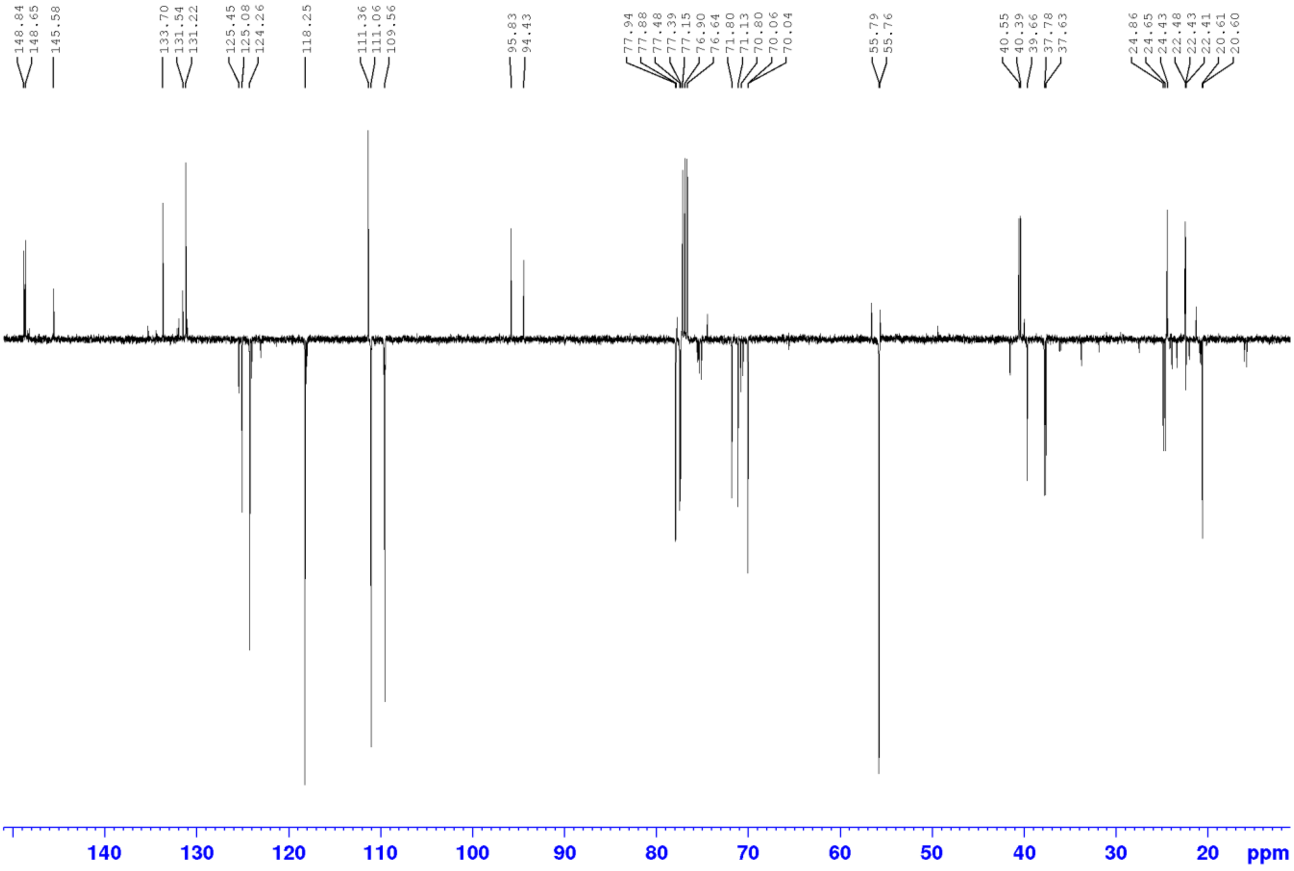

MO-164-2;CDCl3

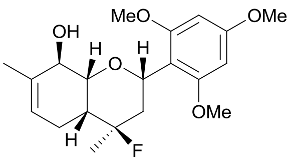


(*R*)-8e

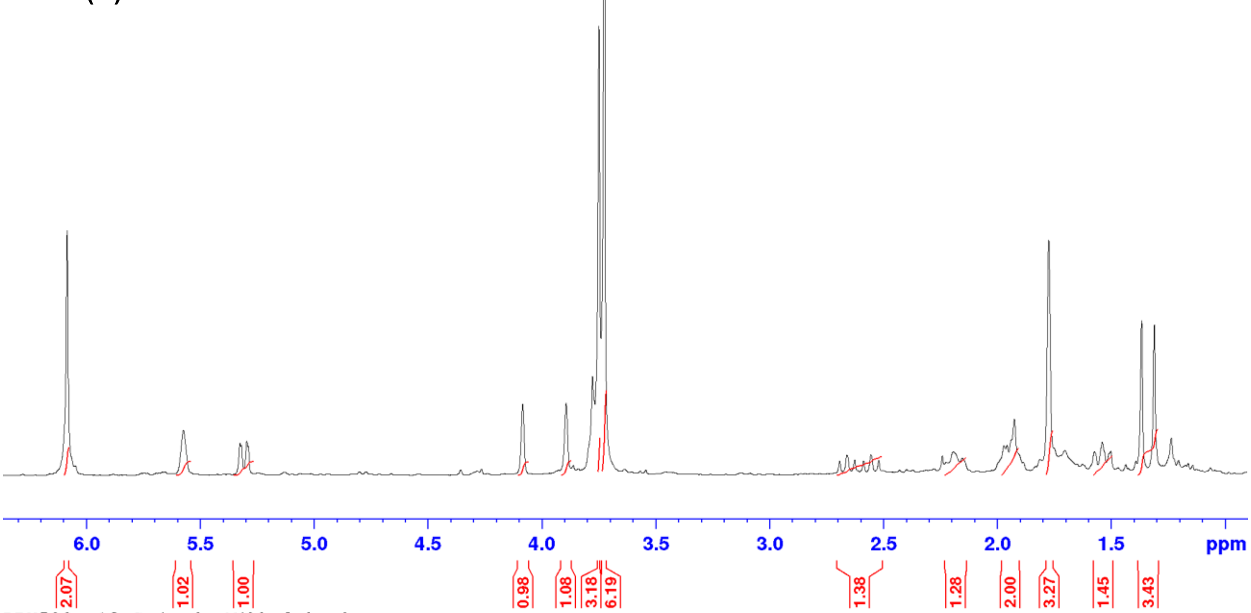


DRX500, 13 C-jmod, Mikhalchenko,
MO-164-2 in CDCl3, 20mg

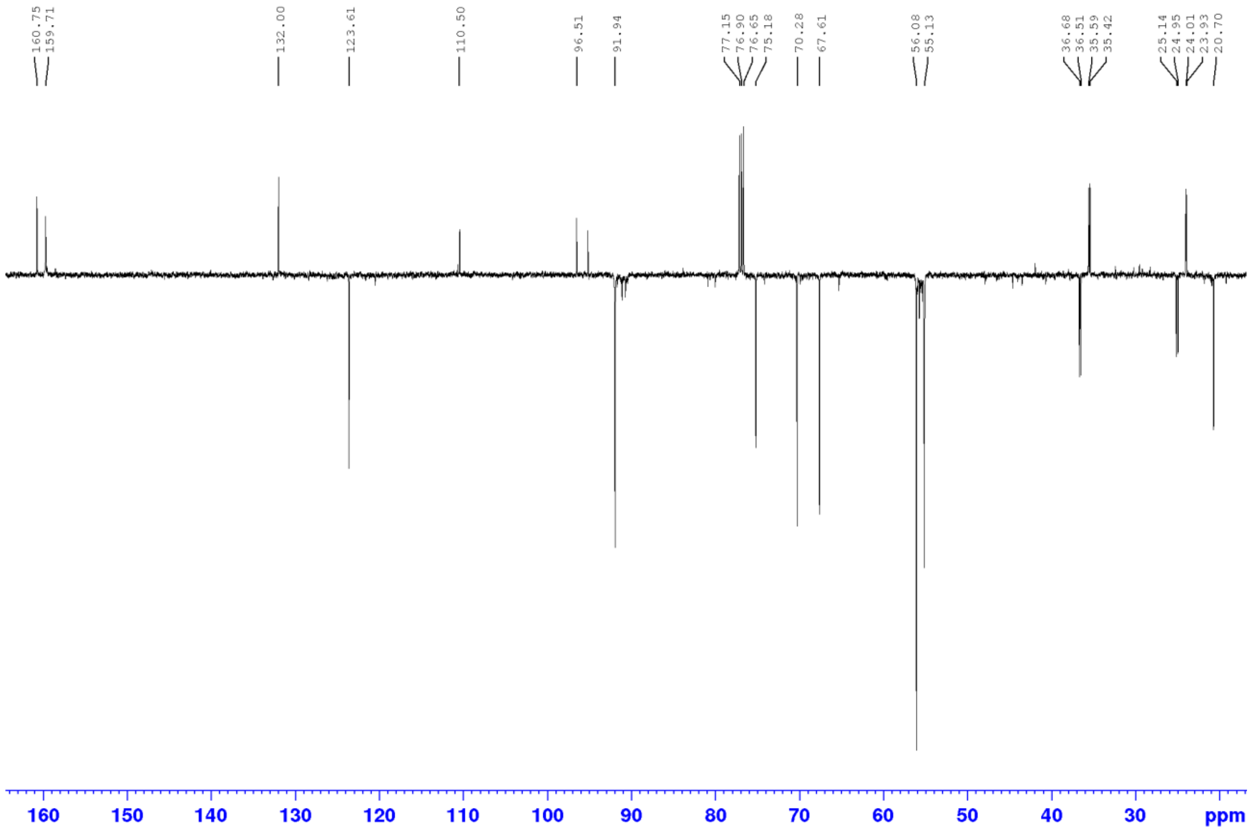

DRX500, 1H, Mikhalchenko, MO-165-2-4 in
CDCl3, 17mg (16.10.2013g)

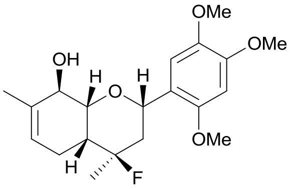


(*R*)-8f

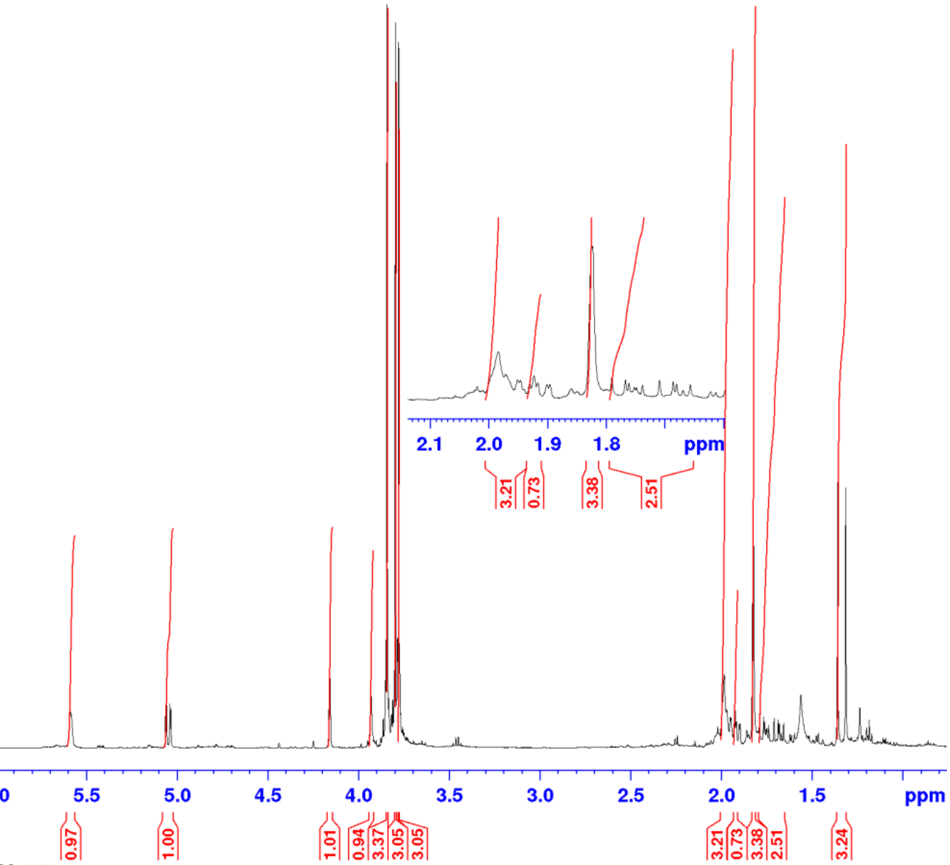


DRX500 C13 jmod 125.7578053 MHz,
Mikhalchenko, MO-165-2-4 in CDCl3, 17mg

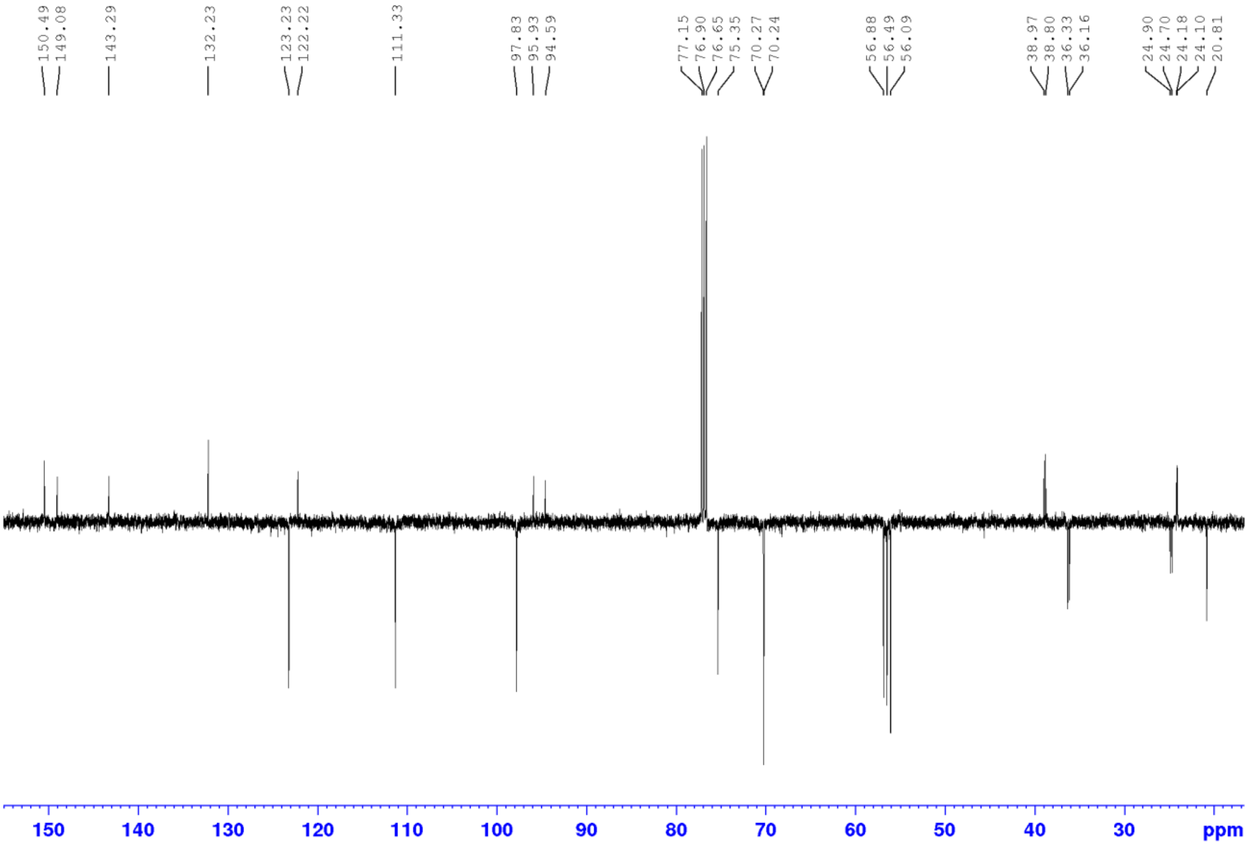

MO-174-7-1;CDCl3

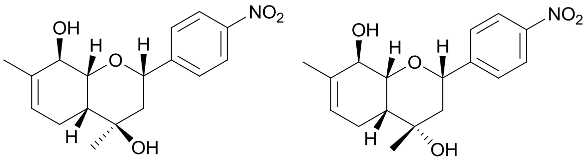


(*R*)-2g (*S*)-2g

(*R*) : (*S*) = 1 : 1.4

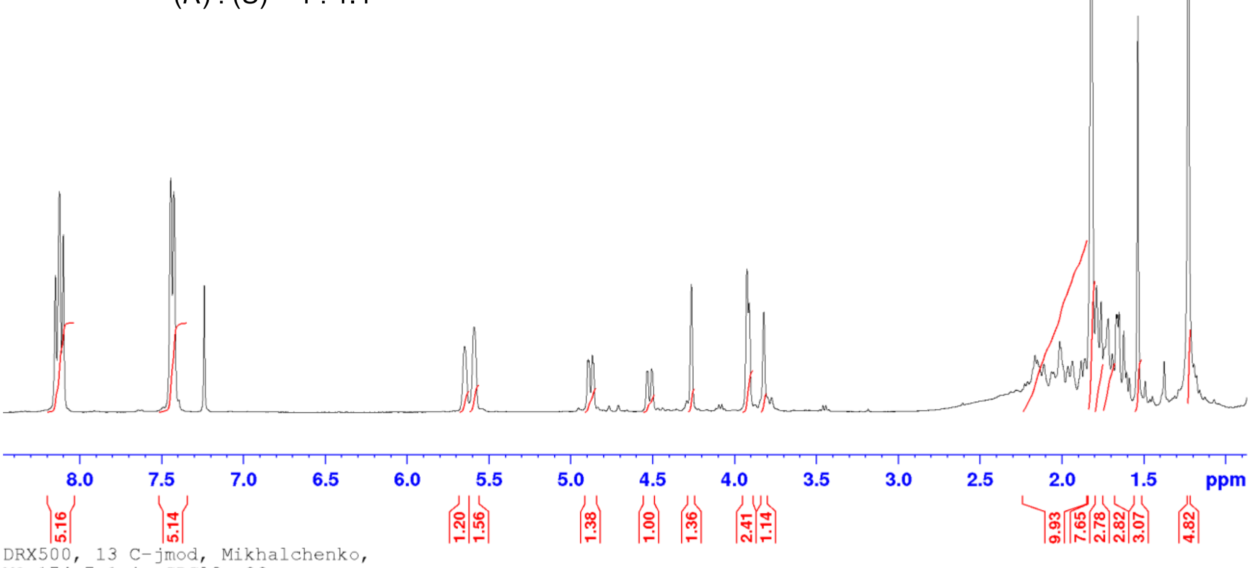


DRX500, 13 C-jmod, Mikhalchenko,
MO-174-7-1 in CDCl3, 20mg

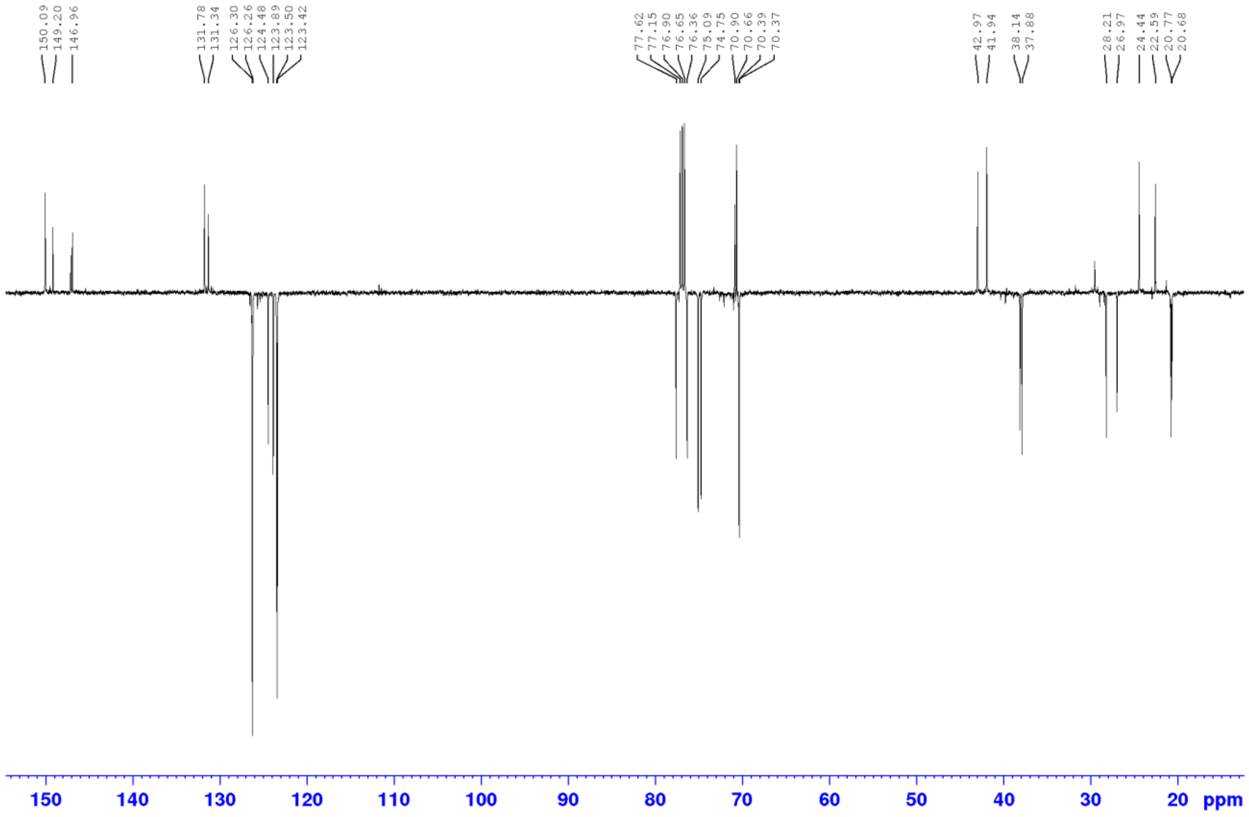

MO-174-4;CDCl3

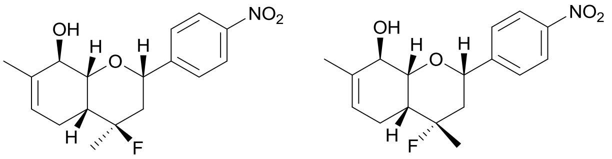


(*R*)-8g (*S*)-8g

(*R*) : (*S*) = 12 : 1

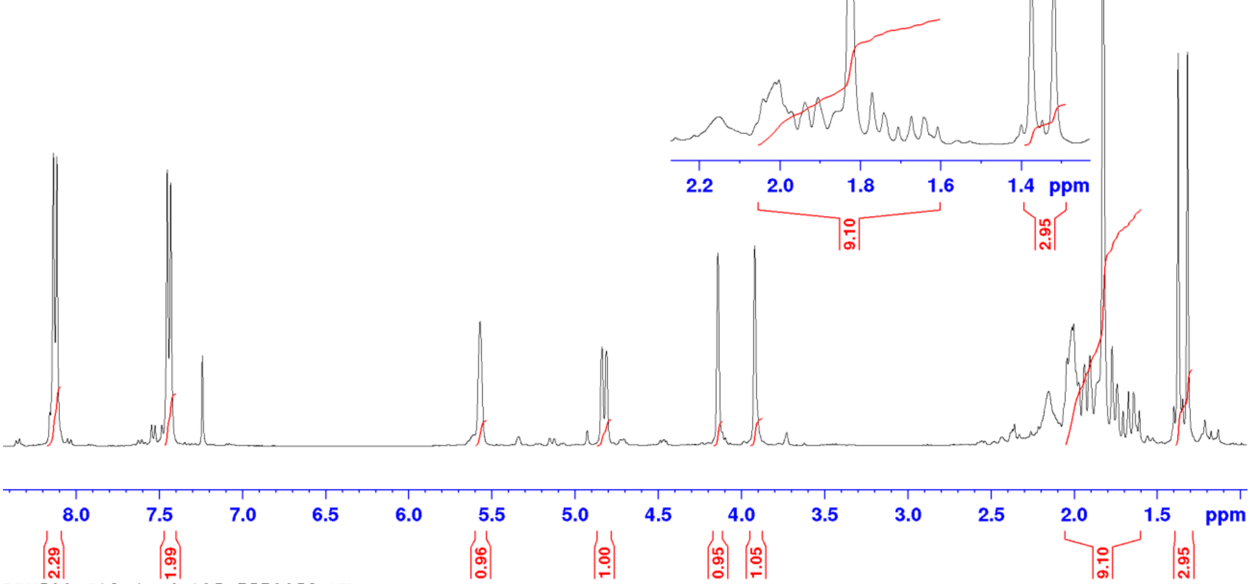


DRX500 C13 jmod 125.7578053 MHz,
Mikhalchenko, MO-174-4 in CDCl3, 55mg

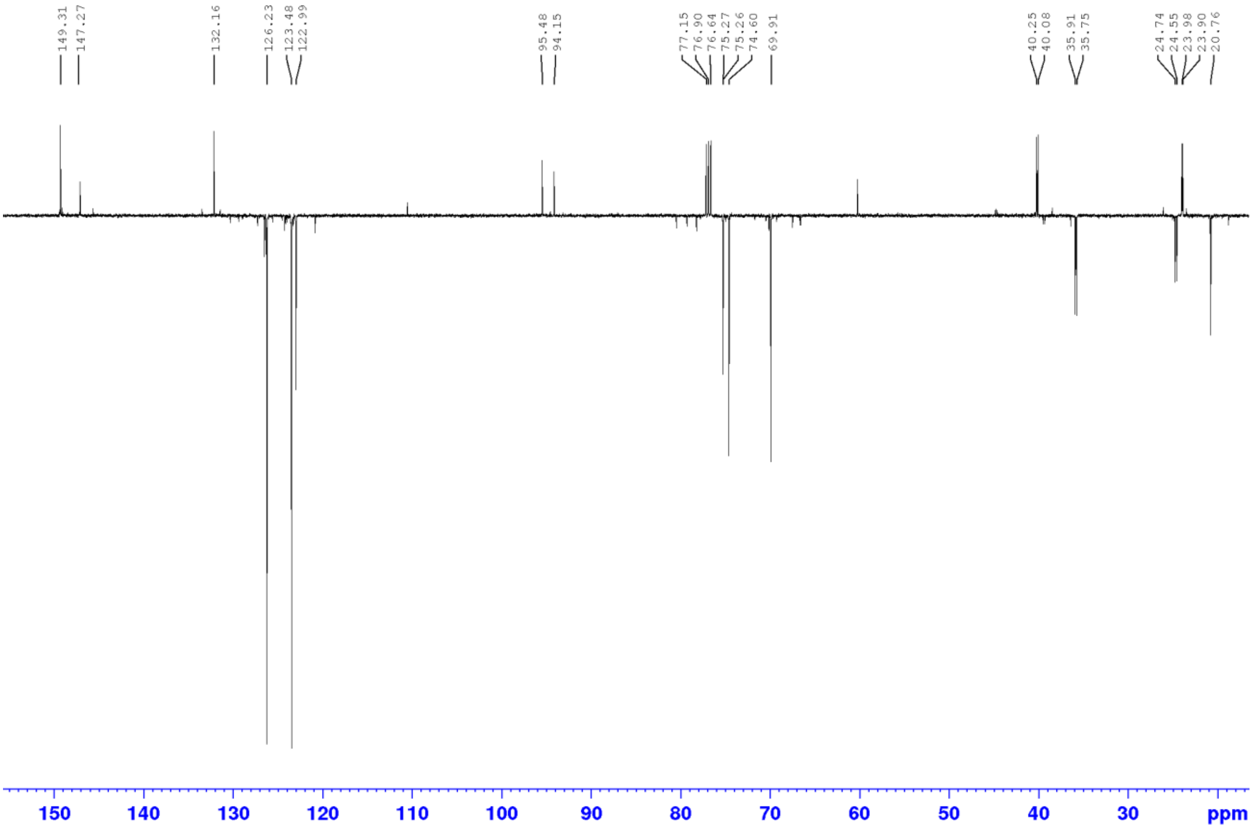

DRX500, 1H, Mikhalchenko, MO-187-6
in CDCl3+CD3OD, 60mg (26.05.2015g.)

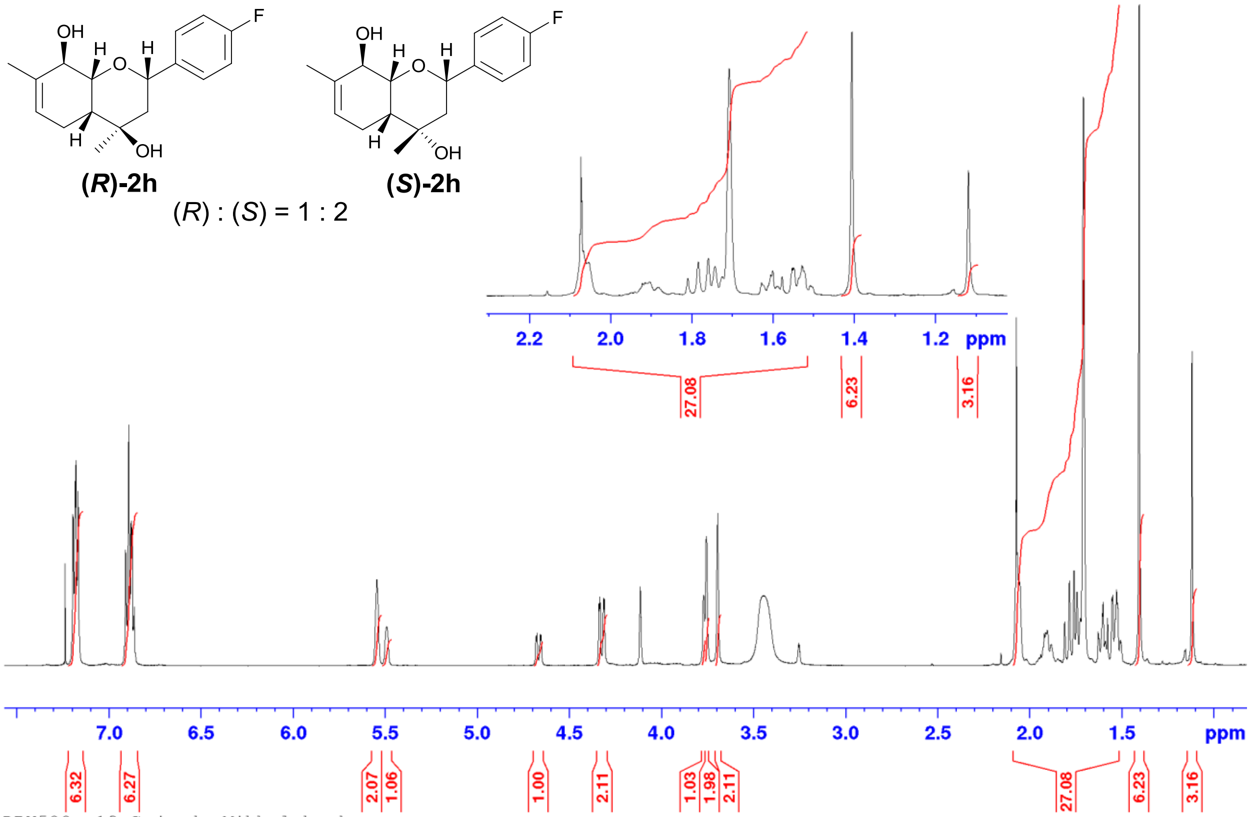


DRX500, 13 C-jmod, Mikhalchenko,
MO-187-6 in CDCl3+CD3OD, 60mg

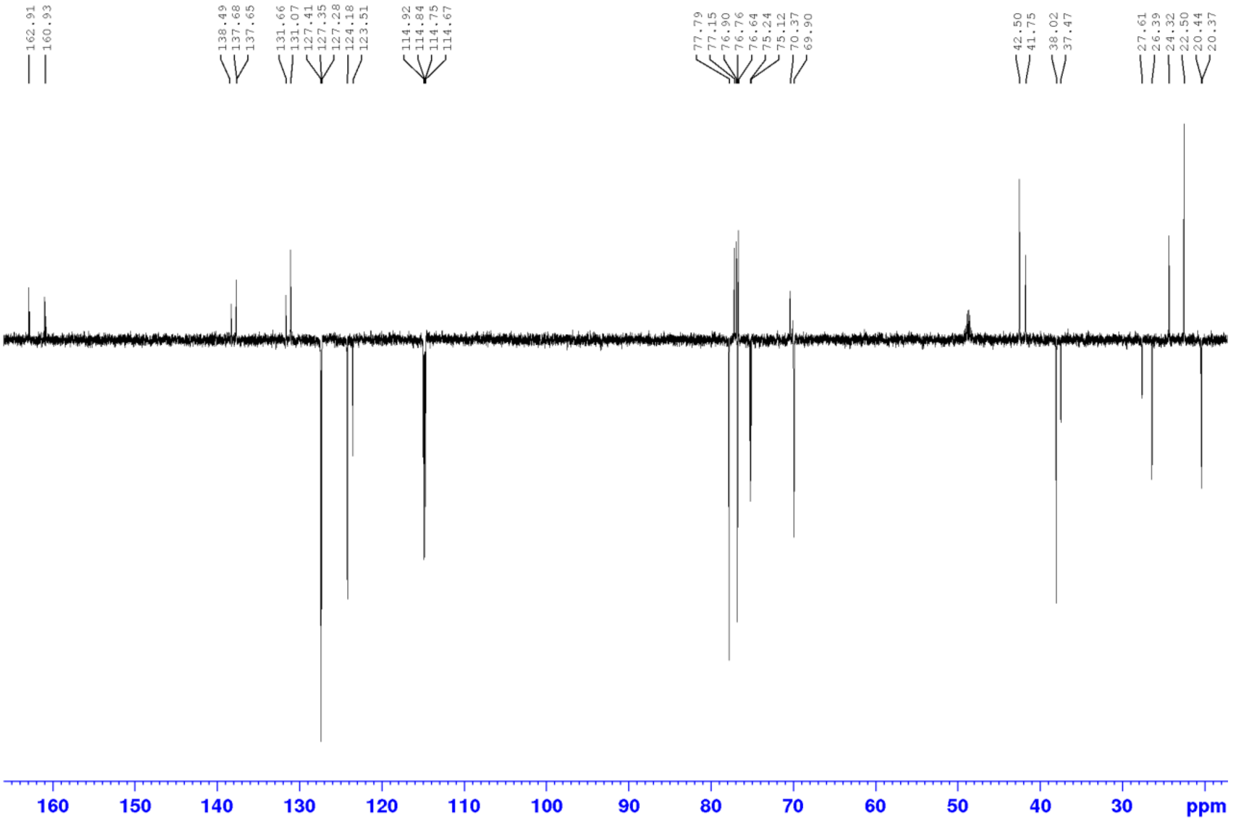

MO - 187 - 4; CDCL3

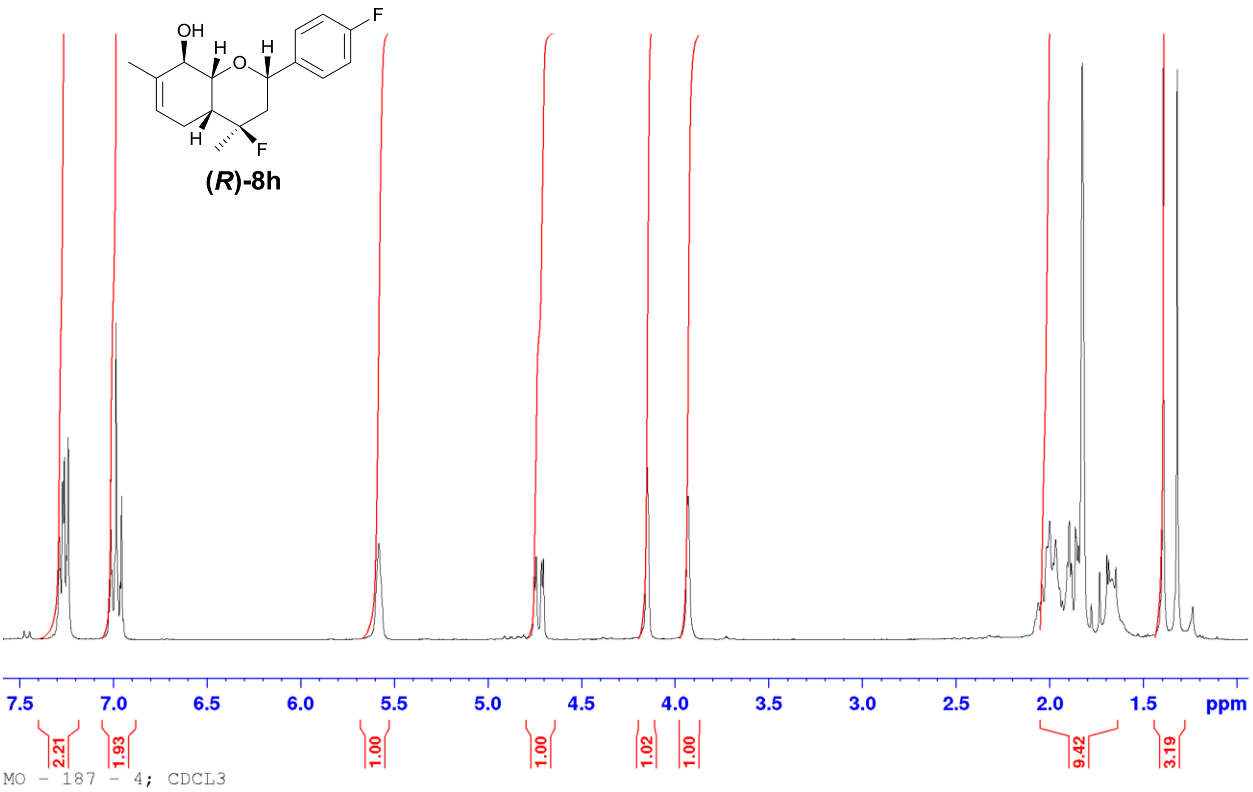


MO - 187 - 4; CDCL3

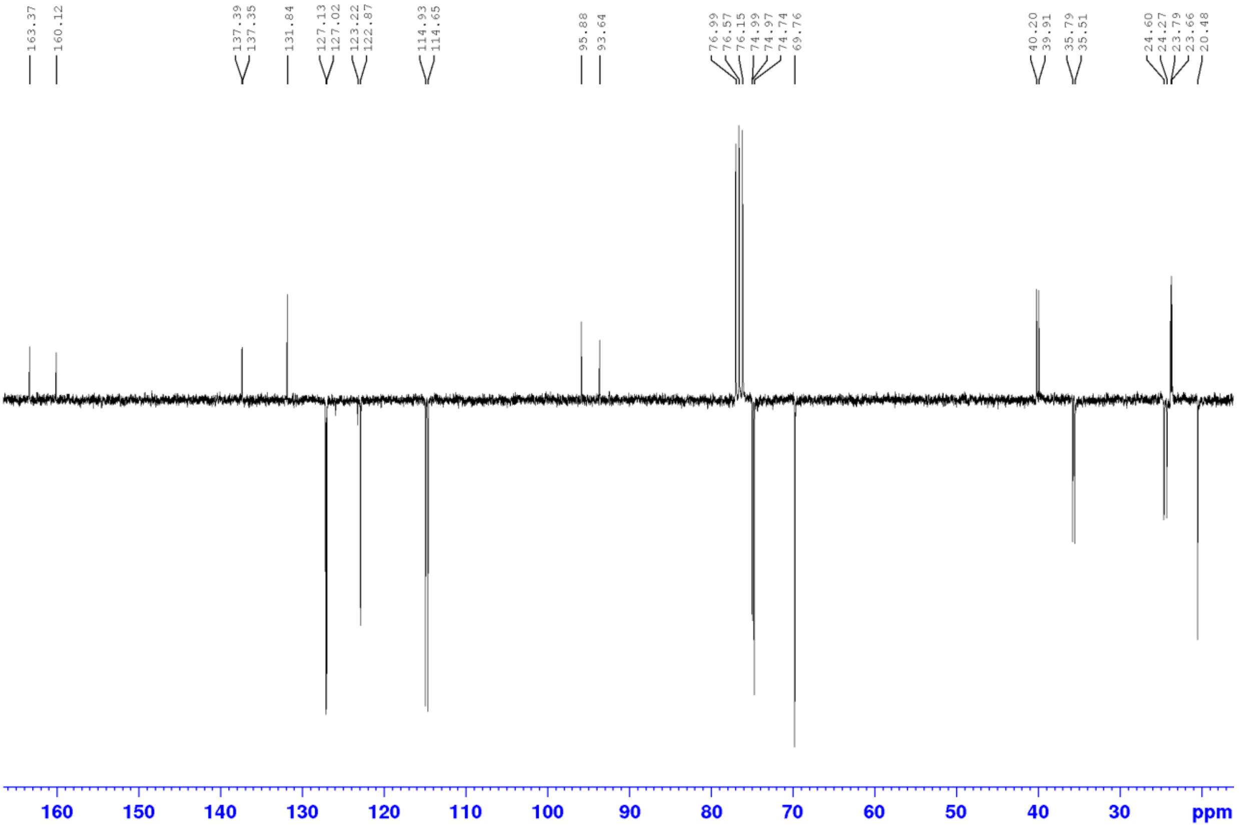

DRX500, 1H, Mikhalchenko, MO-173-7 in
CDCl3+CD3OD, 38mg (19.11.2013g.)

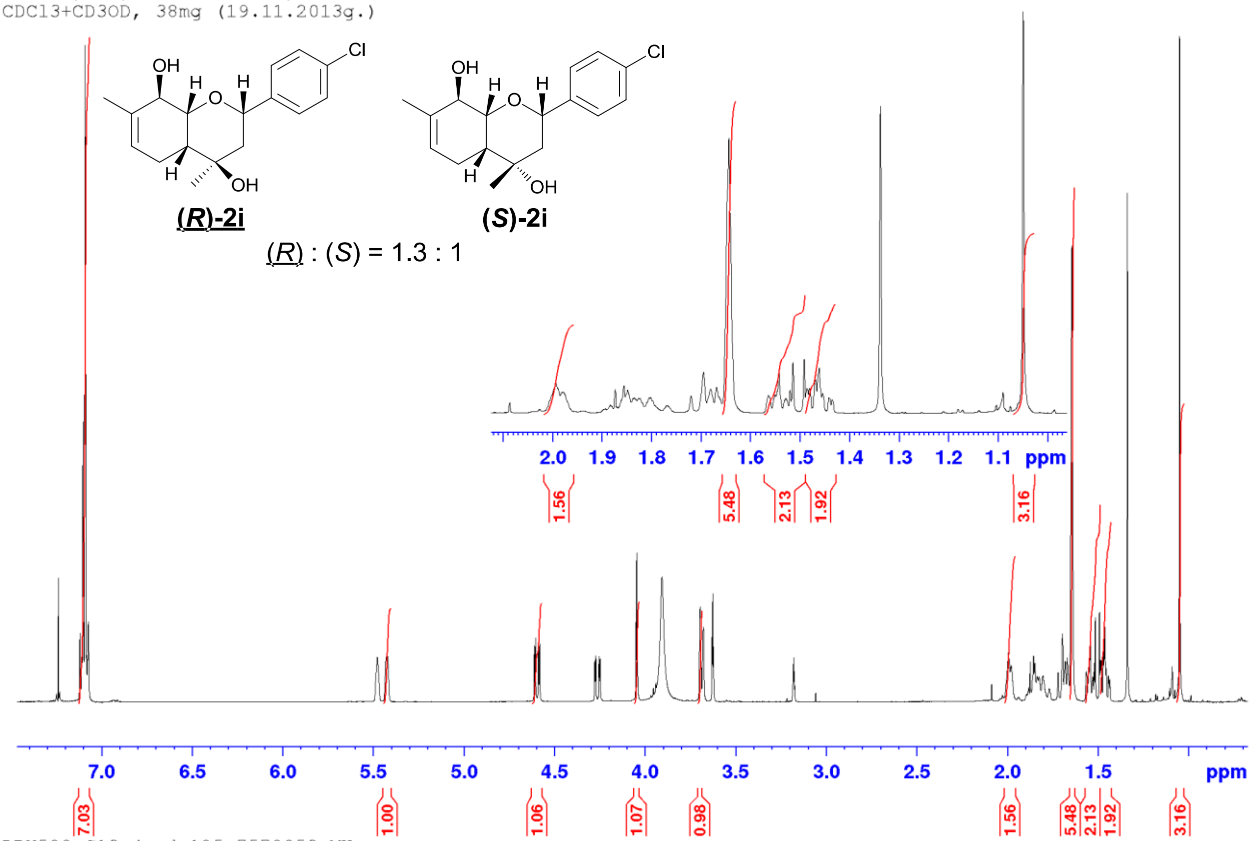


DRX500 C13 jmod 125.7578053 MHz,
Mikhalchenko, MO-173-7 in CDCl3+CD3OD, 38mg

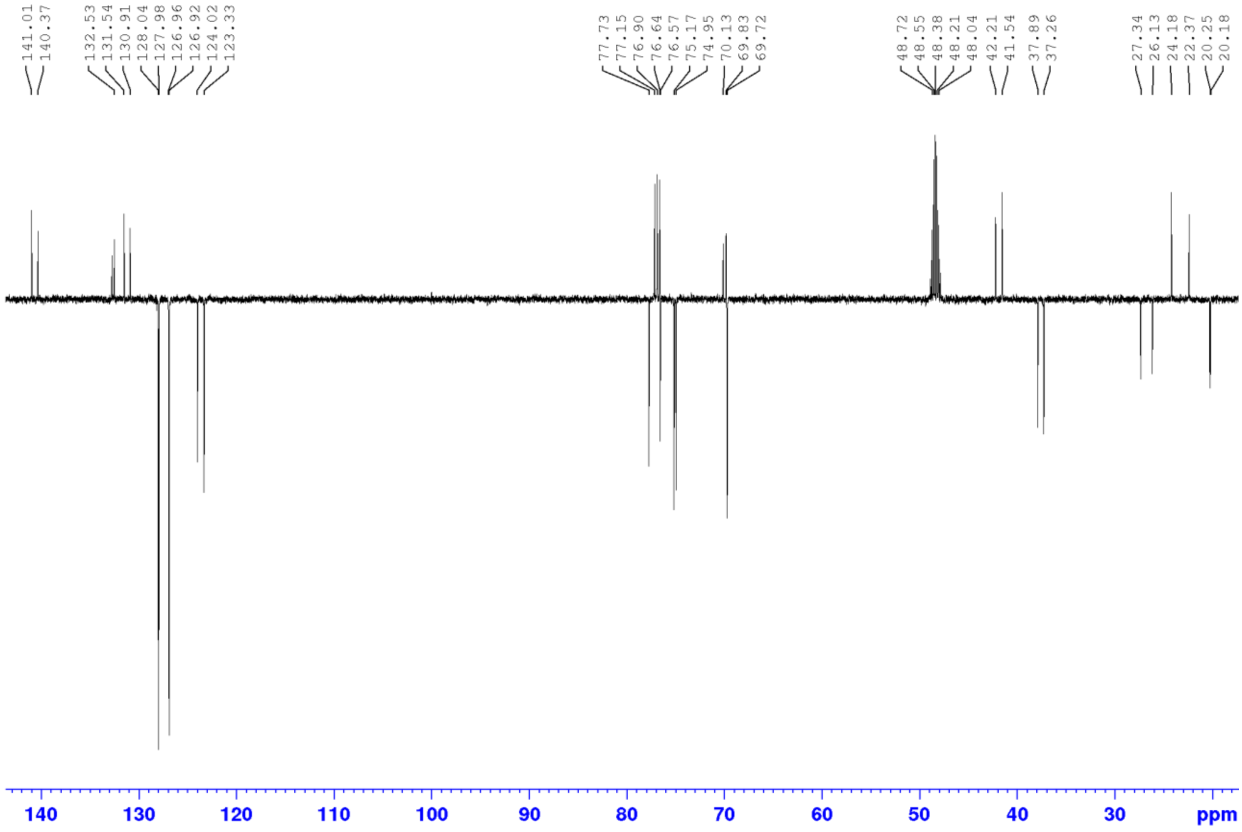

DRX500, 1H, Mikhalchenko, MO-173-8 in
CDCl3+CD3OD, 60mg (19.11.2013g.)

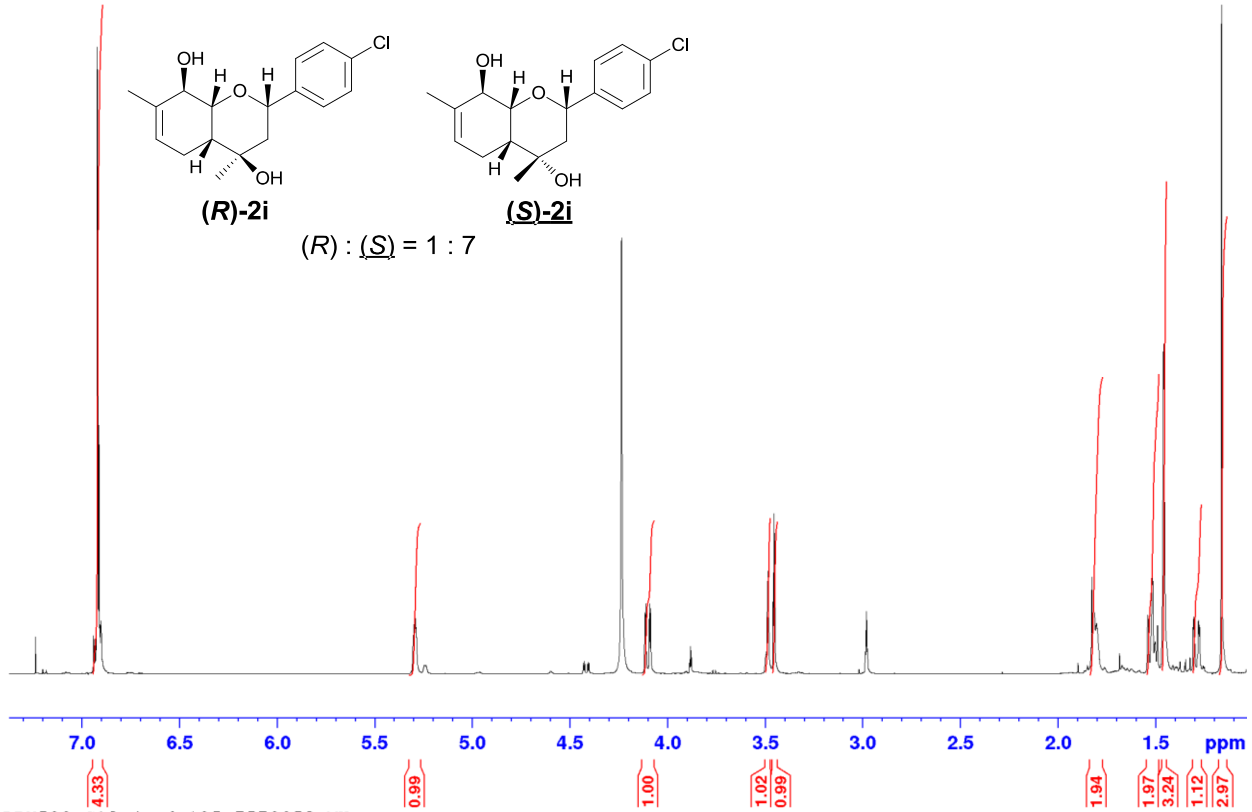


DRX500 C13 jmod 125.7578053 MHz,
Mikhalchenko, MO-173-8 in CDCl3+CD3OD, 60mg

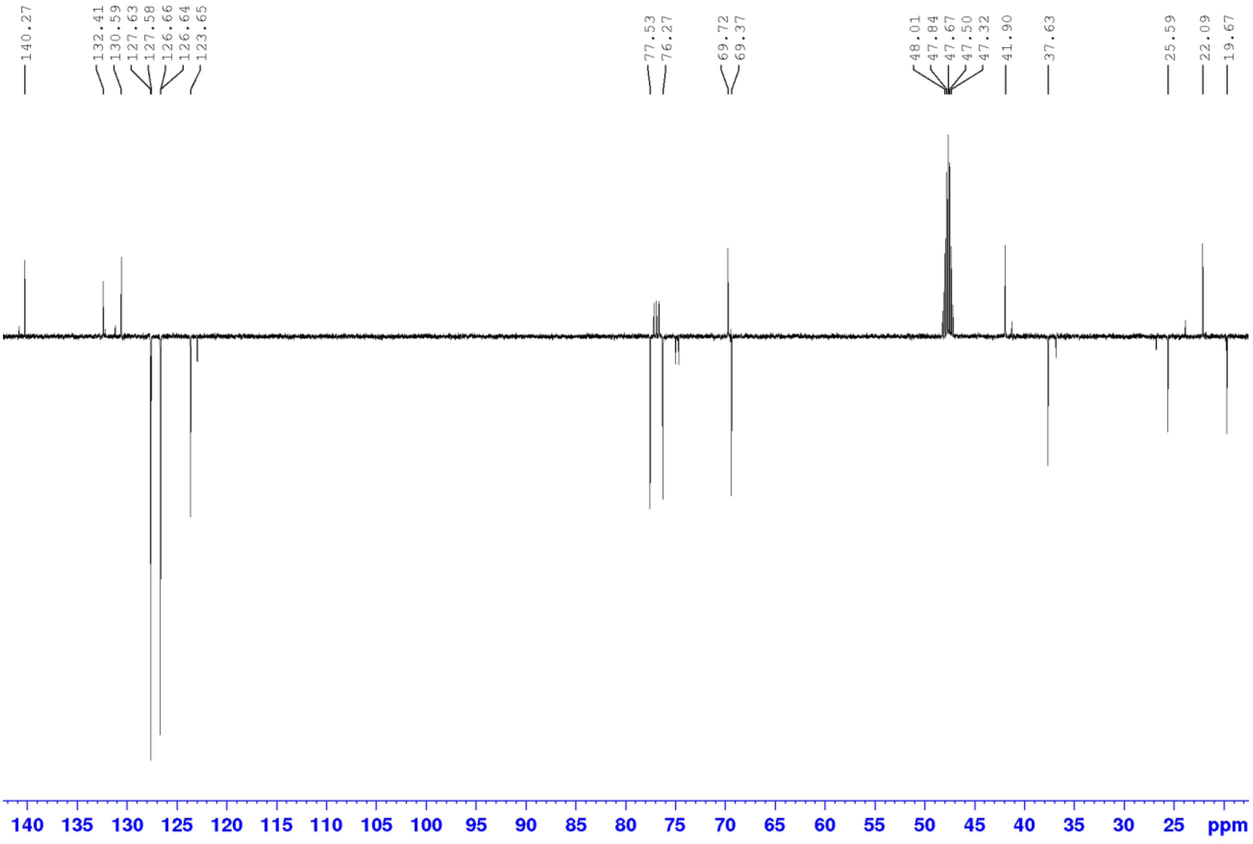

MO-173-3;CDCl3

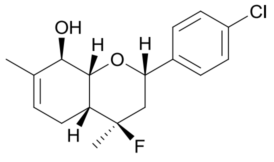


(*R*)-8i

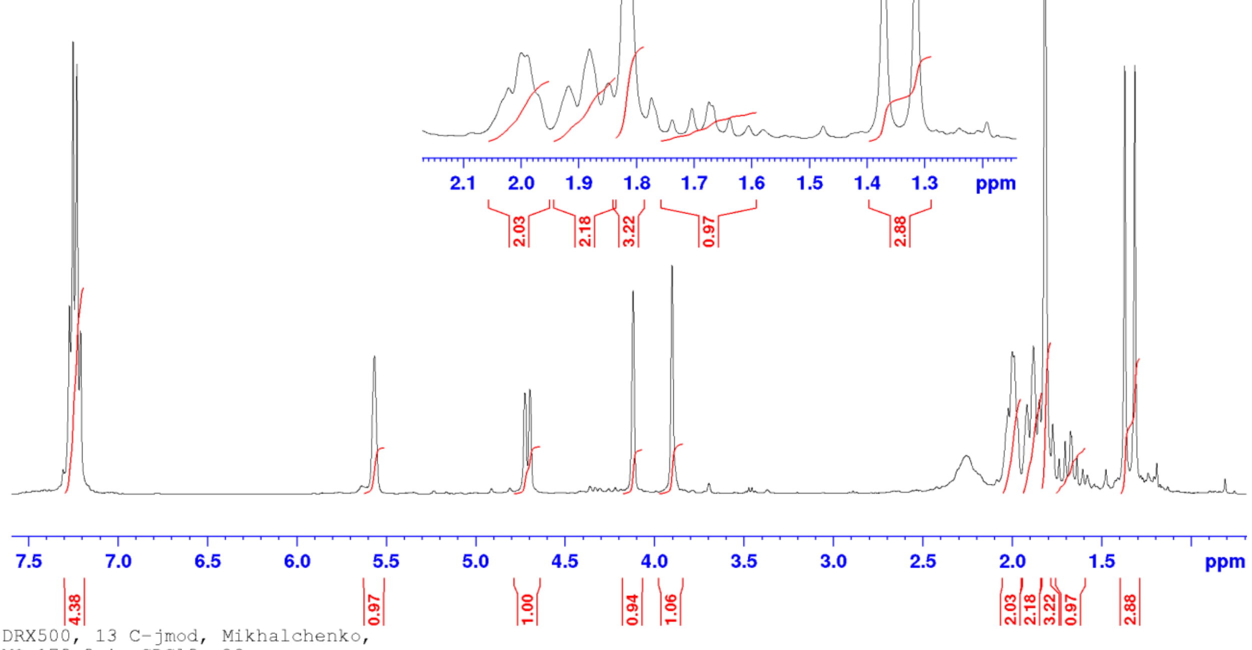


DRX500, 13 C-jmod, Mikhalchenko,
MO-173-3 in CDCl3, 20mg

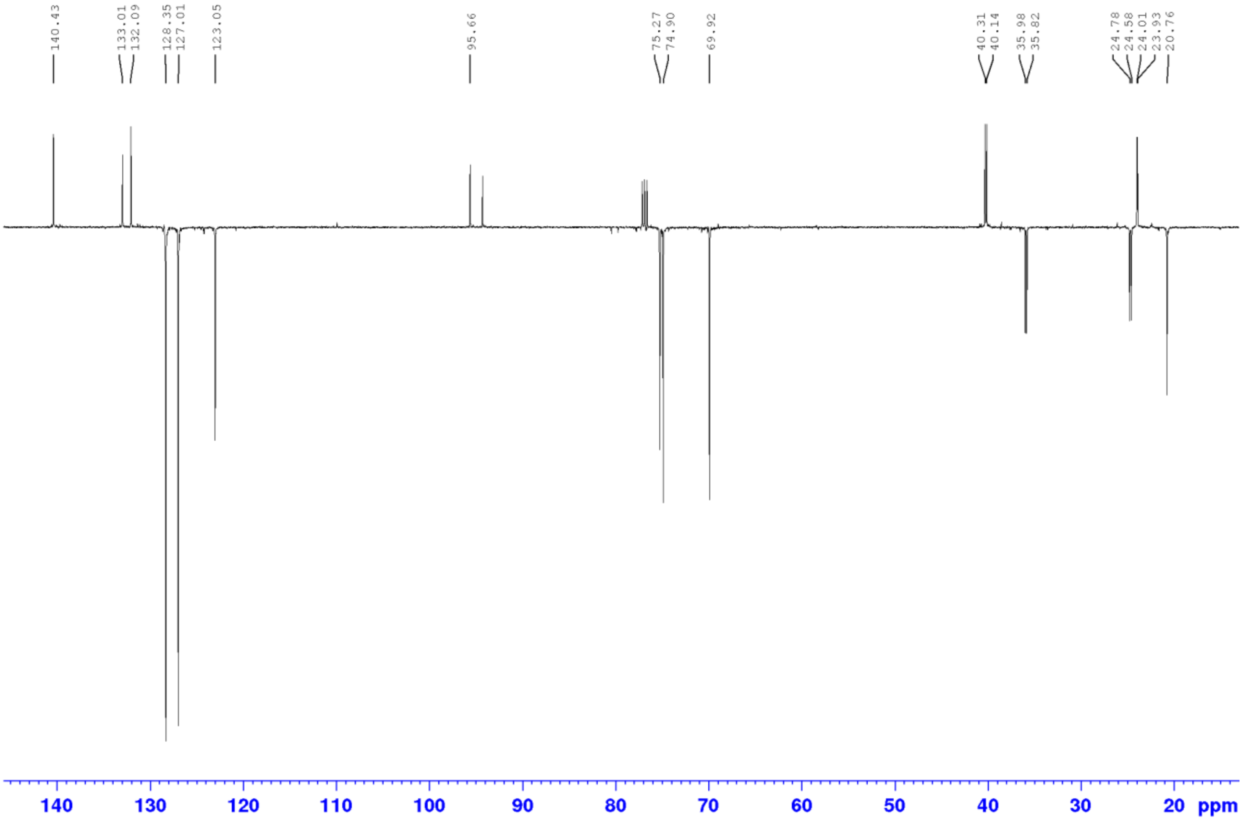

mo-173-4; CDCL3

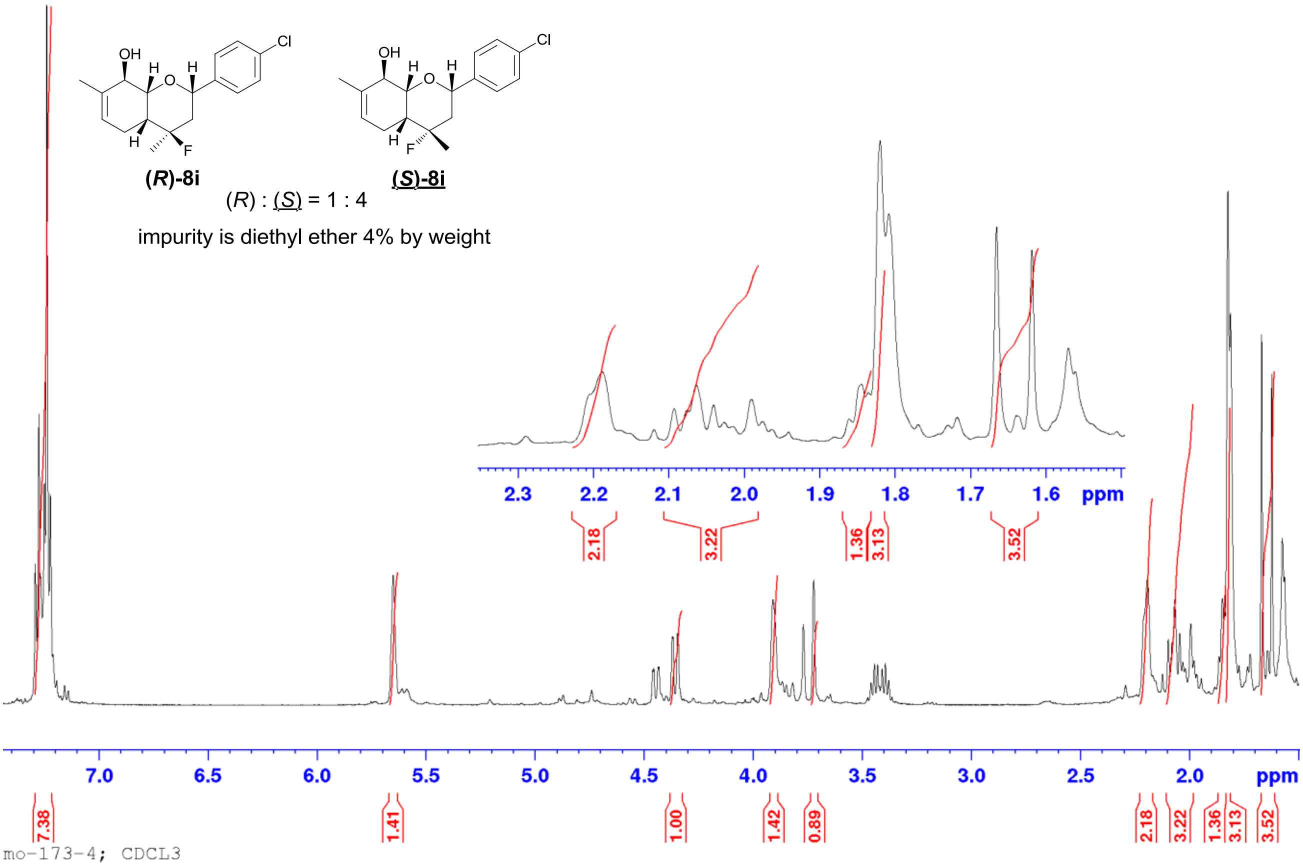


mo-173-4; CDCL3

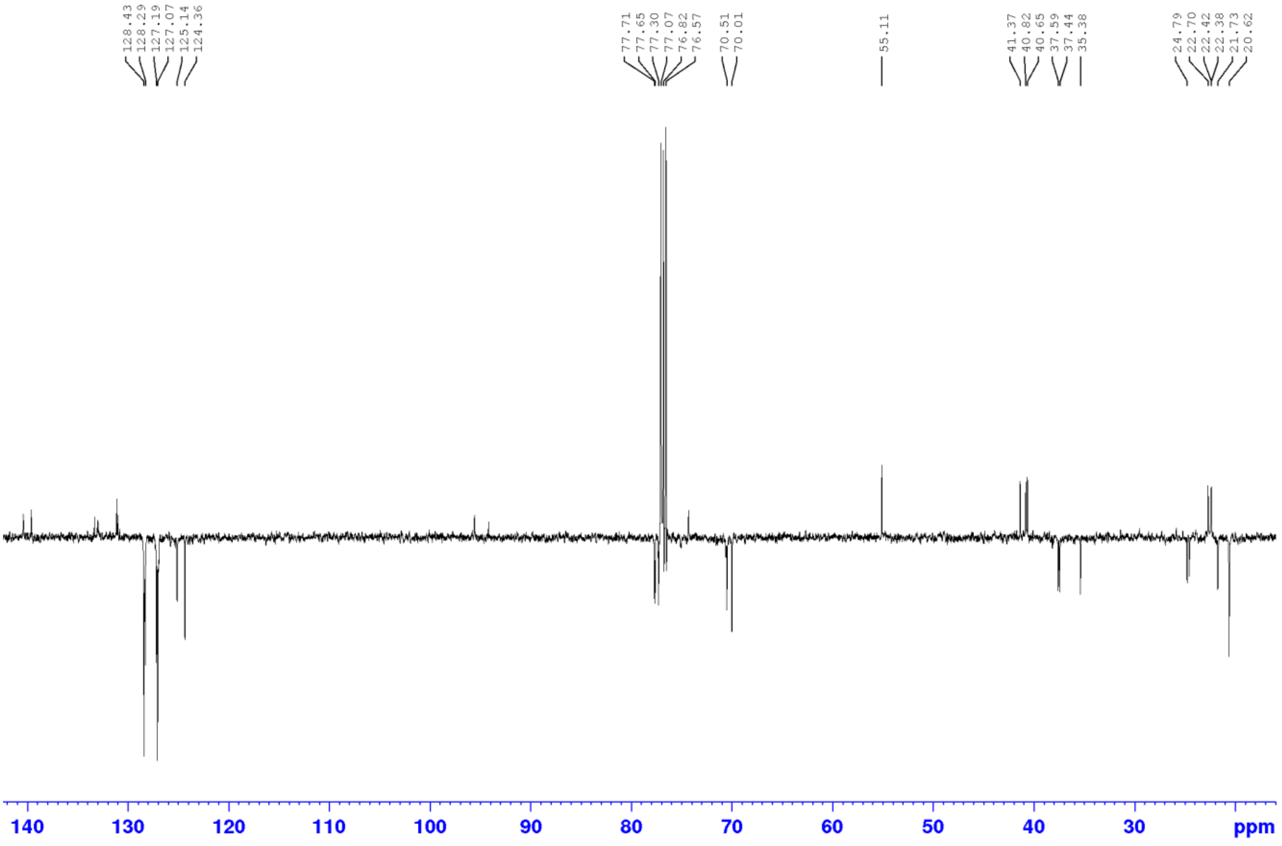

DRX500, 1H, Mikhalchenko, MO-175-5 in CD3OD, 20mg (14.01.2014g.

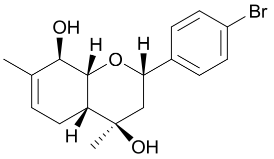


**(*R*)-2j**

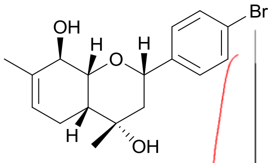


**(*S*)-2j**

(*R*) : (*S*) = 1 : 2

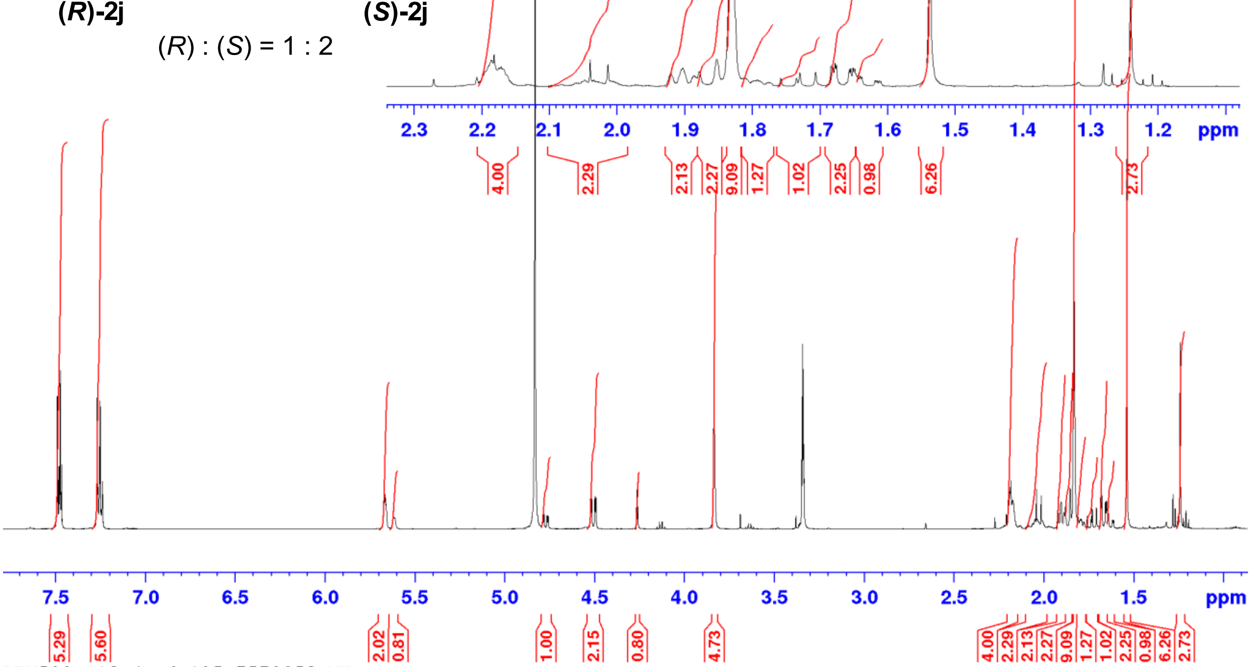


DRX500 C13 jmod 125.7578053 MHz,
Mikhalchenko, MO-175-5 in CD3OD, 20mg

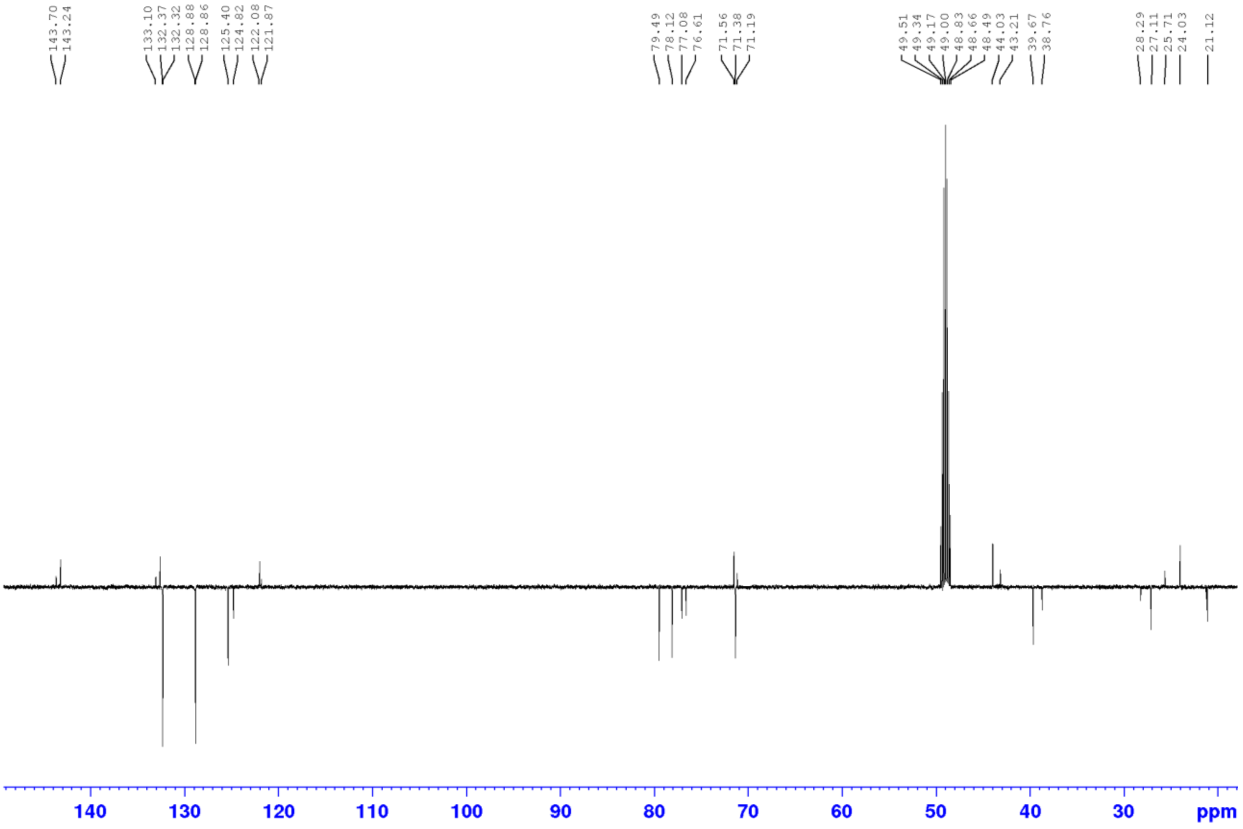

MO-175-3;CDCl3

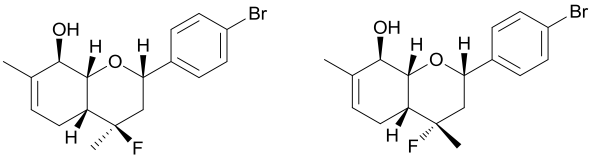


**(*R*)-8j** admixture **(*S*)-8j**

(*R*) : (*S*) = 10 : 1

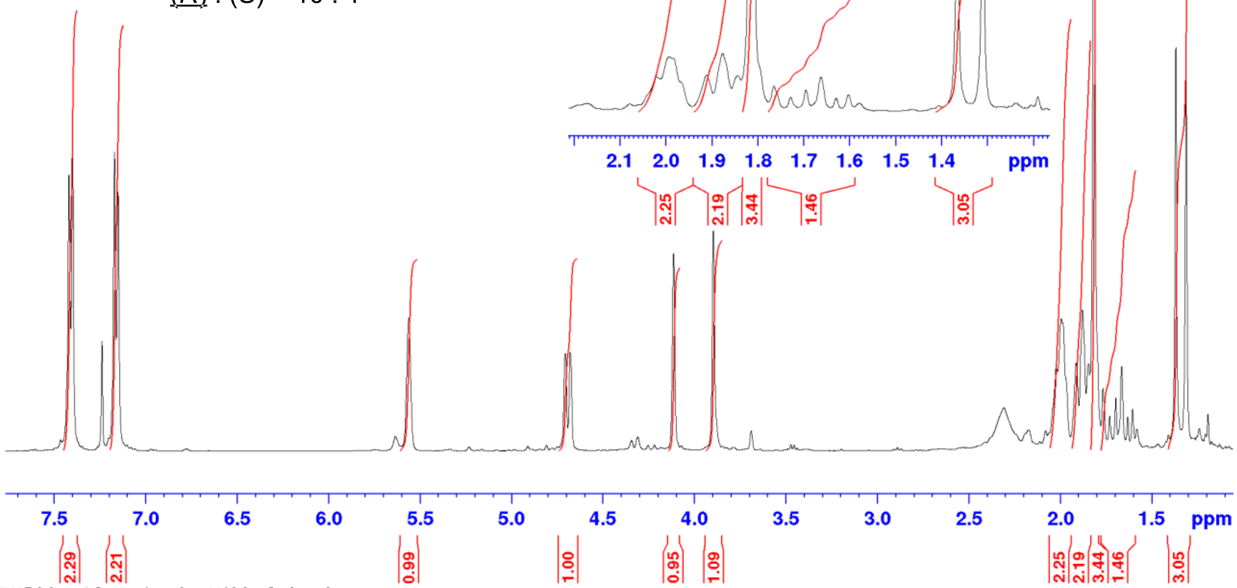


DRX500, 13 C-jmod, Mikhalchenko,
MO-175-3 in CDCl3, 20mg

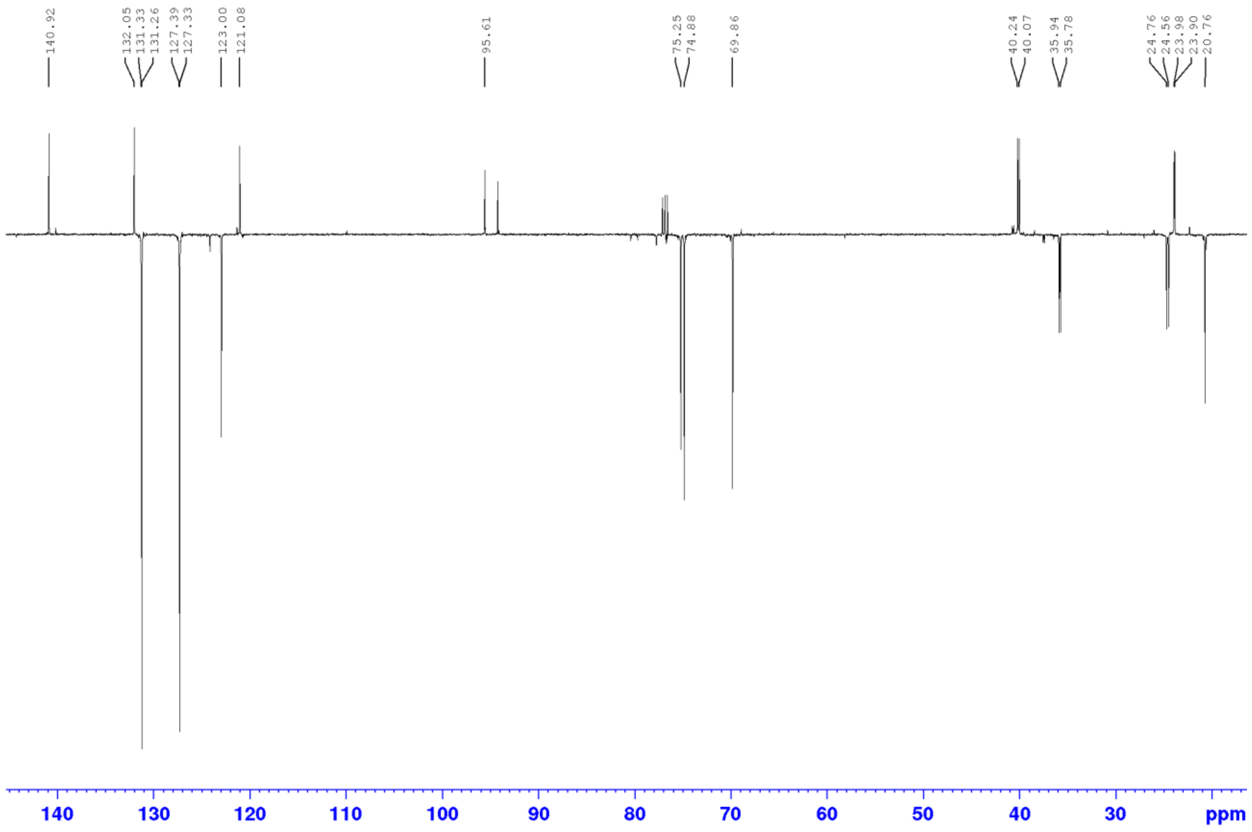

DRX500, 1H, Mikhalchenko, MO-234-3 in CDCl3+CD3OD, 60mg (27.05.2014g.)

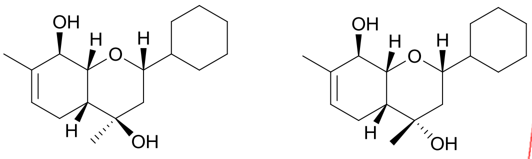


**(*R*)-2k** **(*S*)-2k**

(*R*) : (*S*) = 1 : 3

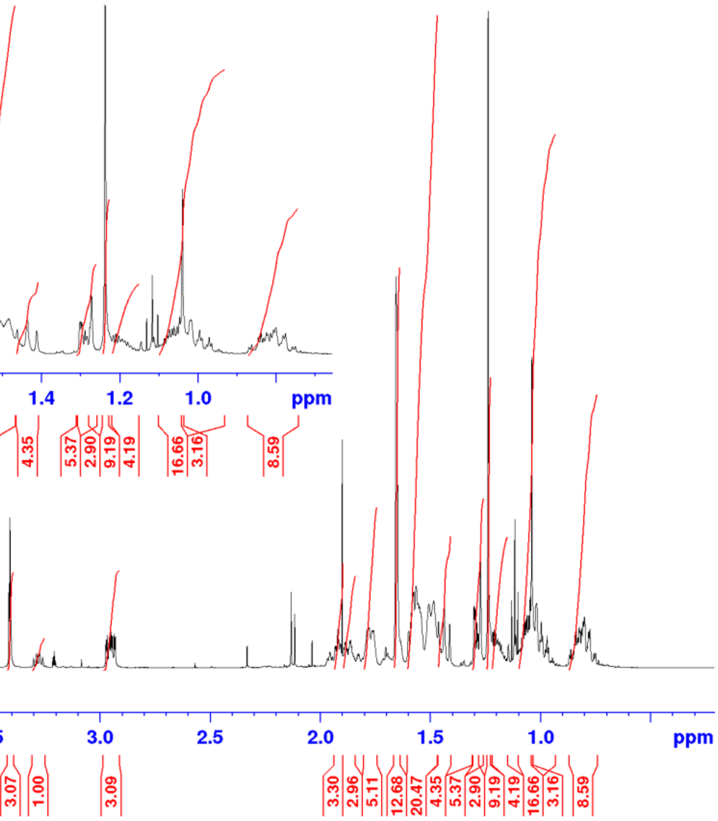


DRX500 C13 jmod 125.7578053 MHz, Mikhalchenko, MO-234-3 in CDCl3+CD3OD, 60mg

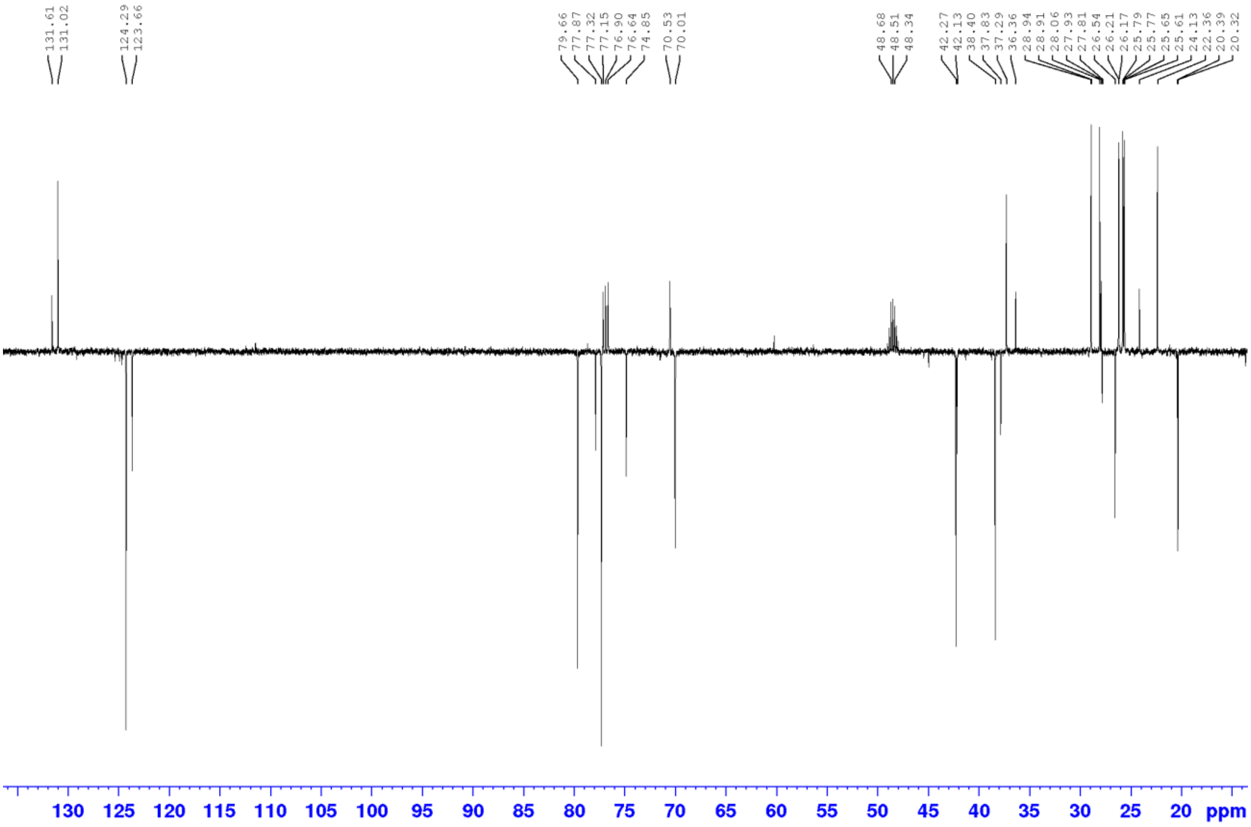

DRX500, 1H, Mikhalchenko, MO-234-1 in CDCl3, 60mg (27.05.2014g.)

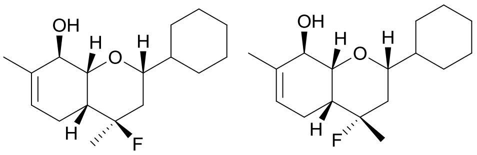


**(*R*)-8k** **(*S*)-8k**

(*R*) : (*S*) = 3 : 1

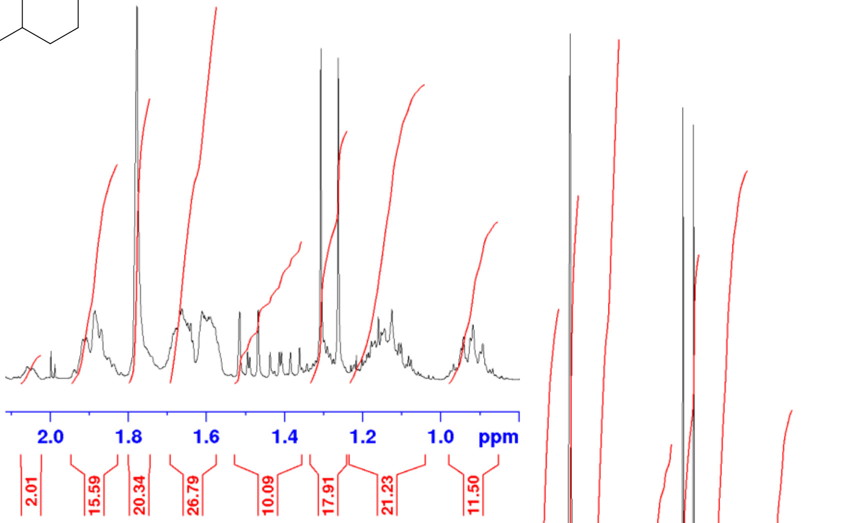


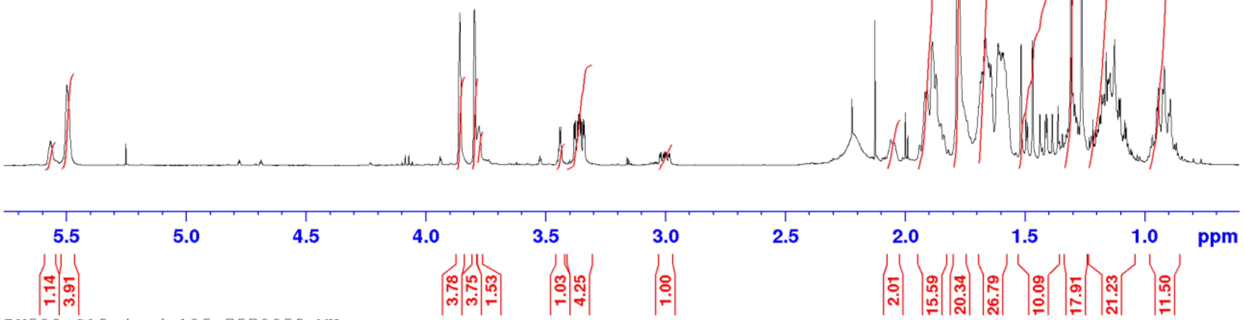


DRX500 C13 jmod 125.7578053 MHz, Mikhalchenko, MO-234-1 in CDCl3, 60mg

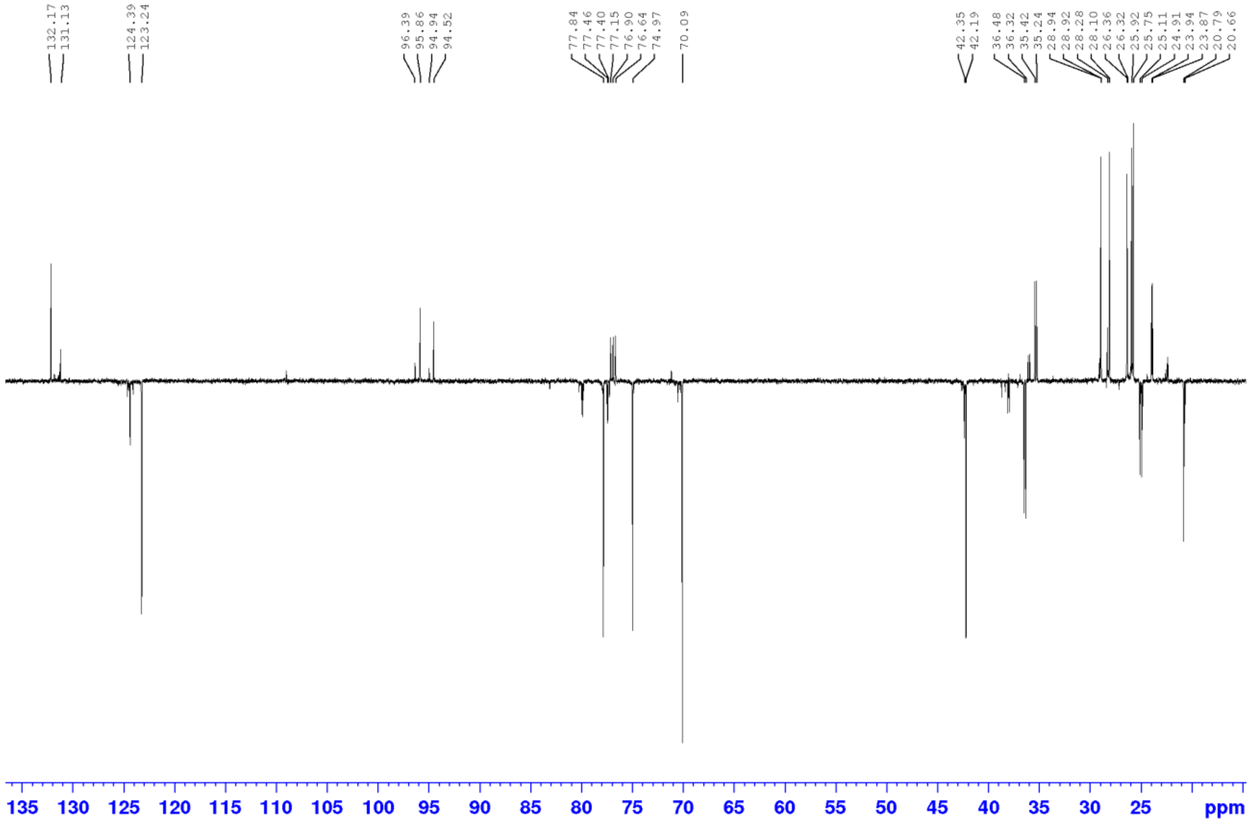

DRX500, 1H, Mikhalchenko, MO-230-1 in
CDCl3, 60mg (04.06.2014g.)

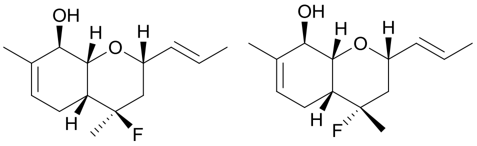

**(*R*)-8l** **(*S*)-8l**

(*R*) : (*S*) = 3 : 1

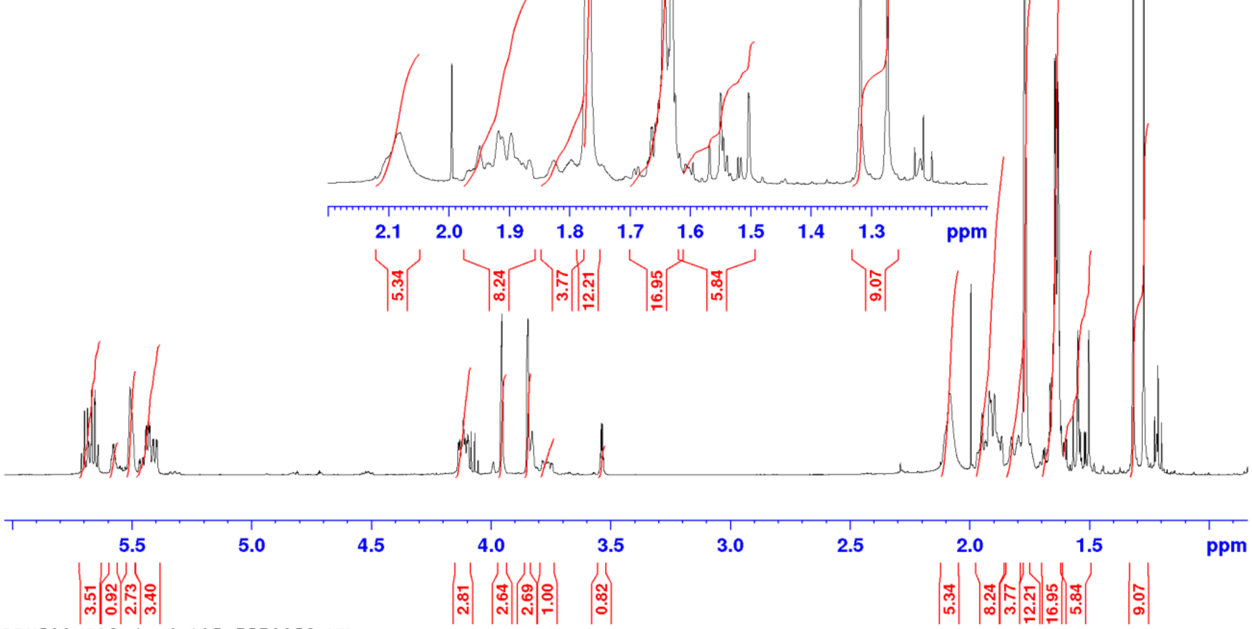

DRX500 C13 jmod 125.7578053 MHz,
Mikhalchenko, MO-230-1 in CDCl3, 60mg

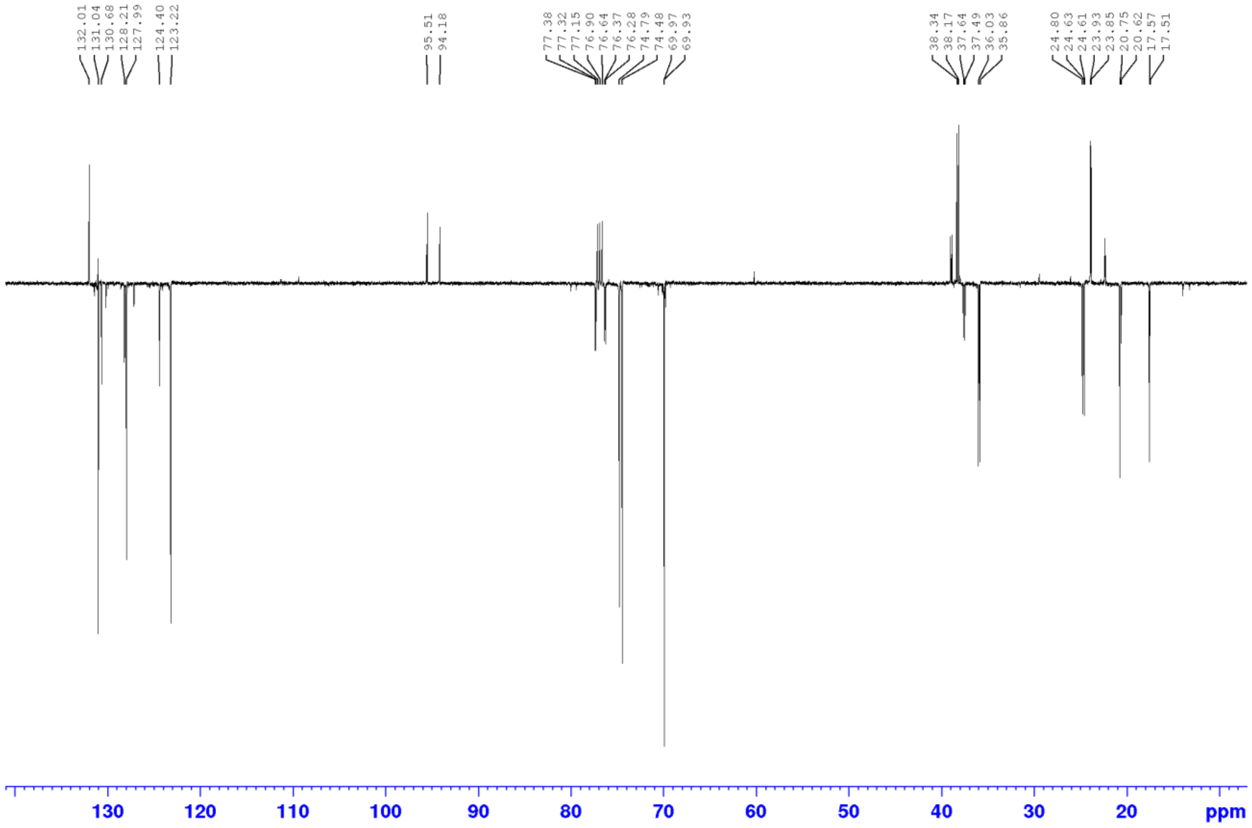

DRX500, 1H, Mikhalchenko,
MO-263-2-1 in CDCl3, 32mg (07.04.2015g.)

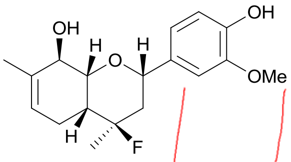


**(*R*)-8m**

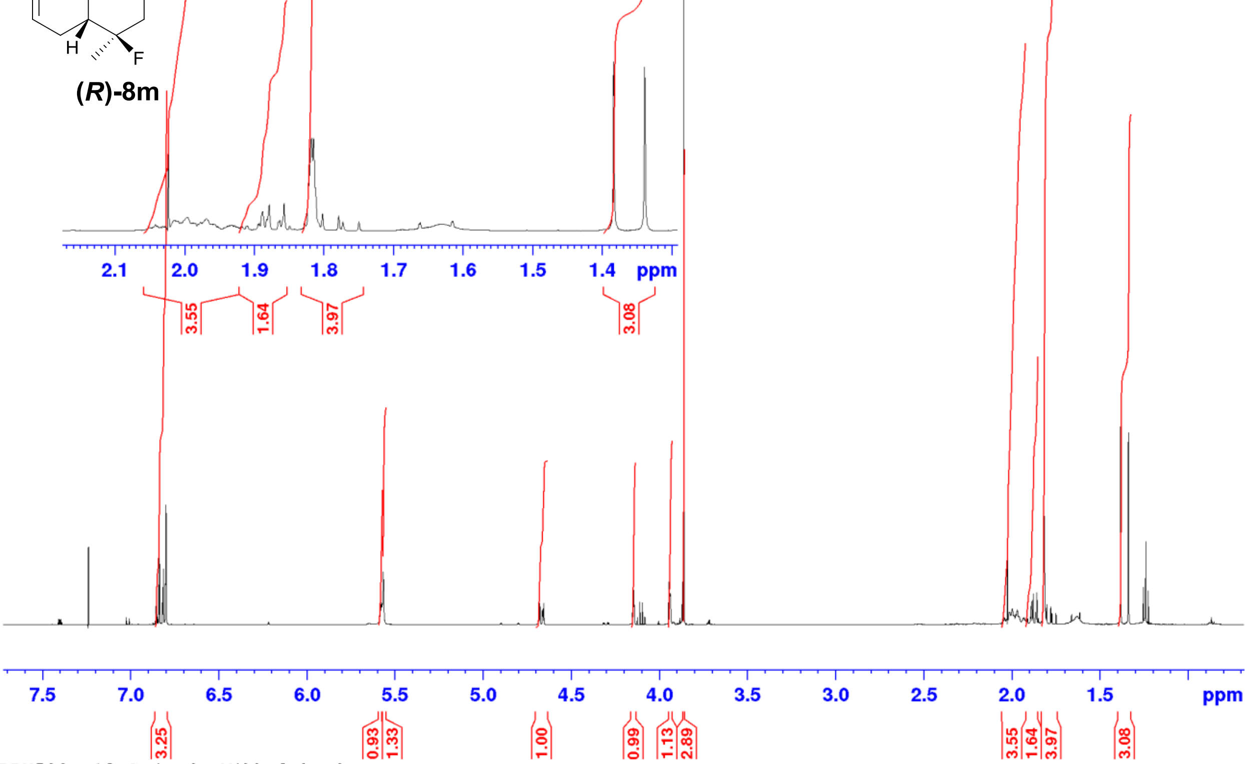


DRX500, 13 C-jmod, Mikhalchenko,
MO-263-2-1 in CDCl3, 32mg

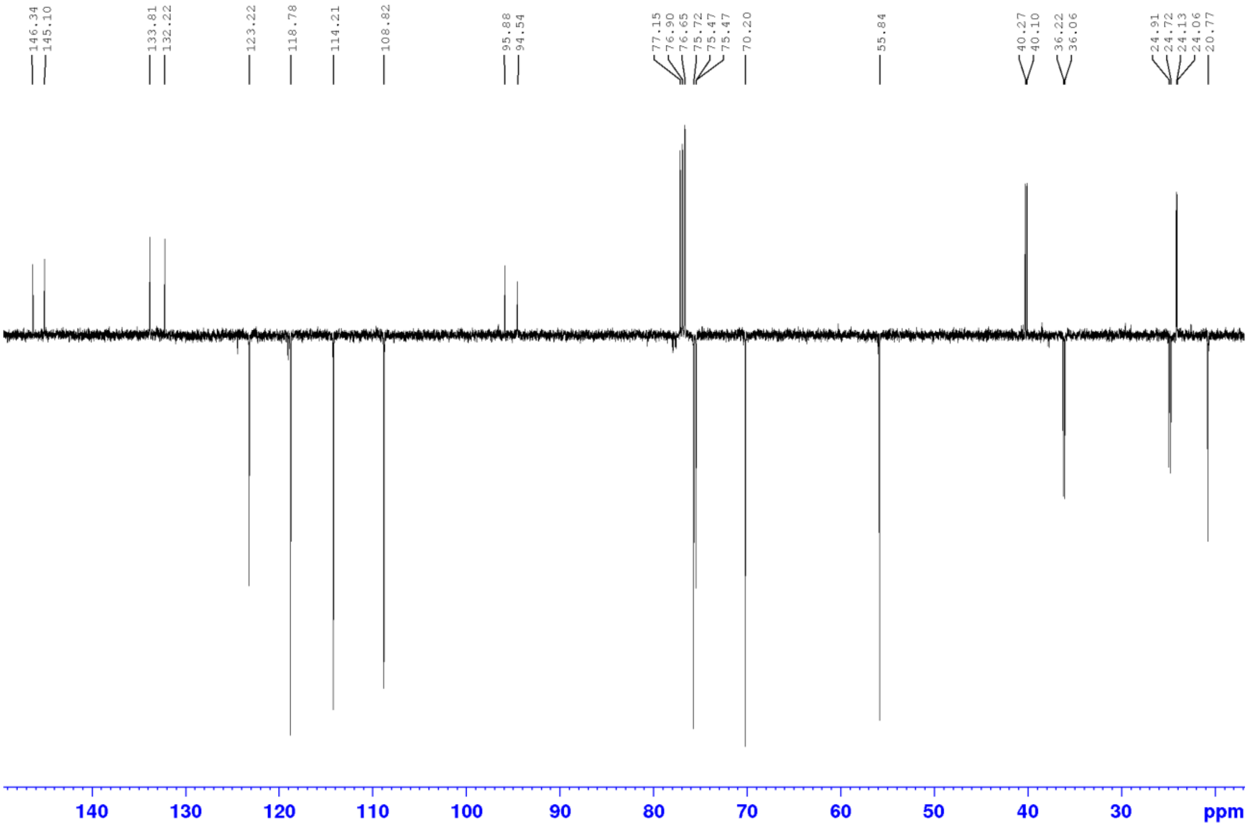

DRX500, 1H, Mikhalchenko,
MO-263-2-2 in CDCl3, 22mg (08.04.2015g.)

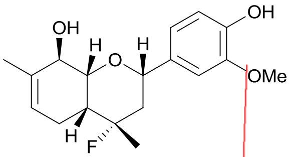


**(*S*)-8m**

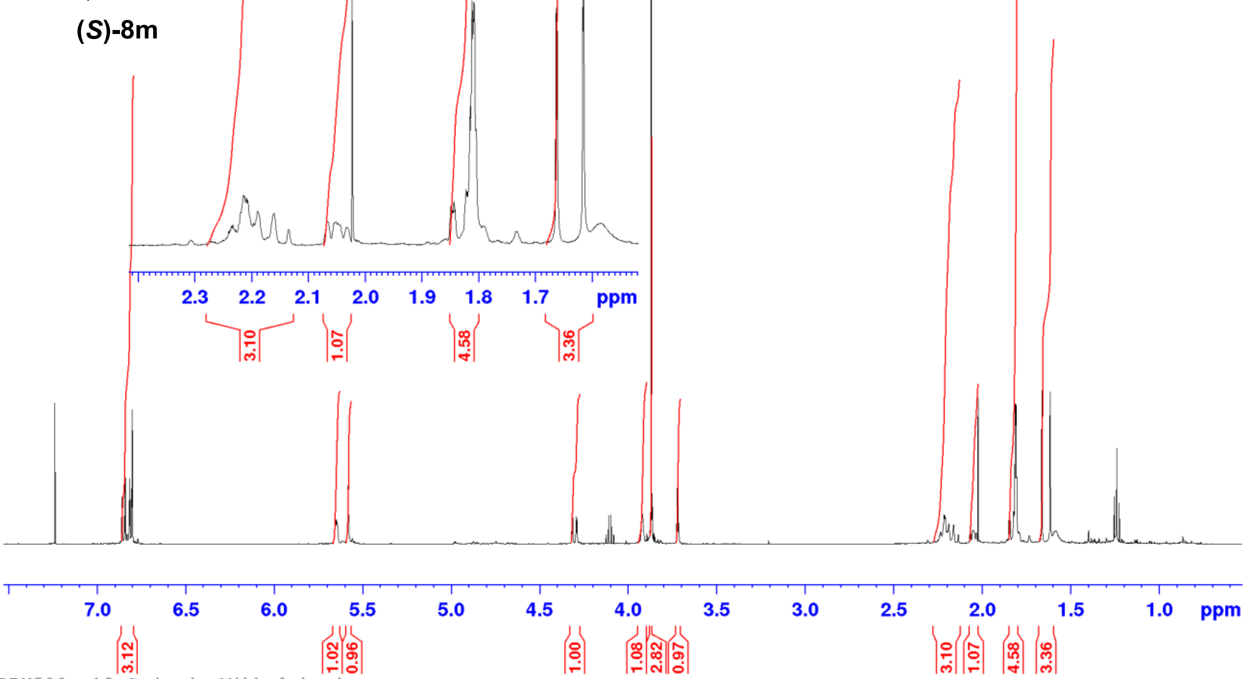


DRX500, 13 C-jmod, Mikhalchenko,
MO-263-2-2 in CDCl3, 22mg

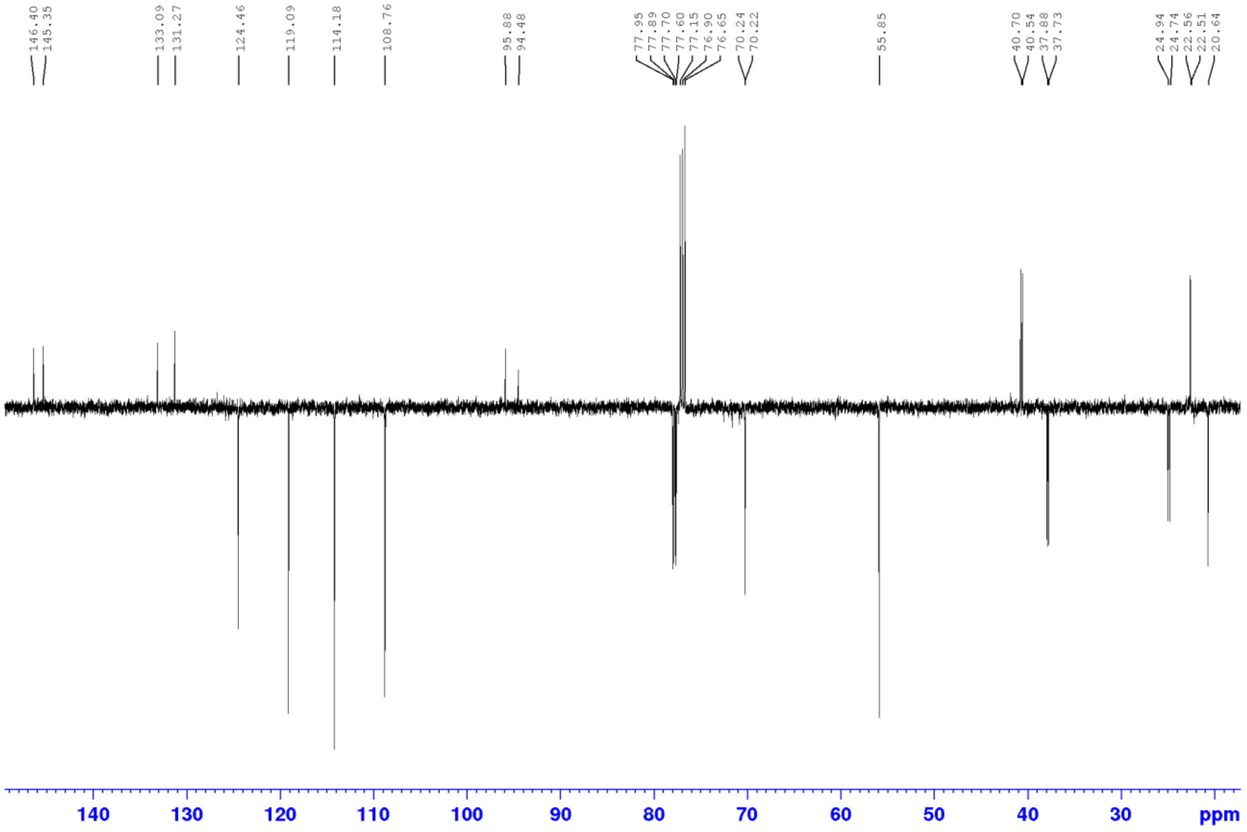

DRX500, 1H, Mikhalchenko, MO-227-3 in
CDCl3, 30mg (25.03.2014g.)

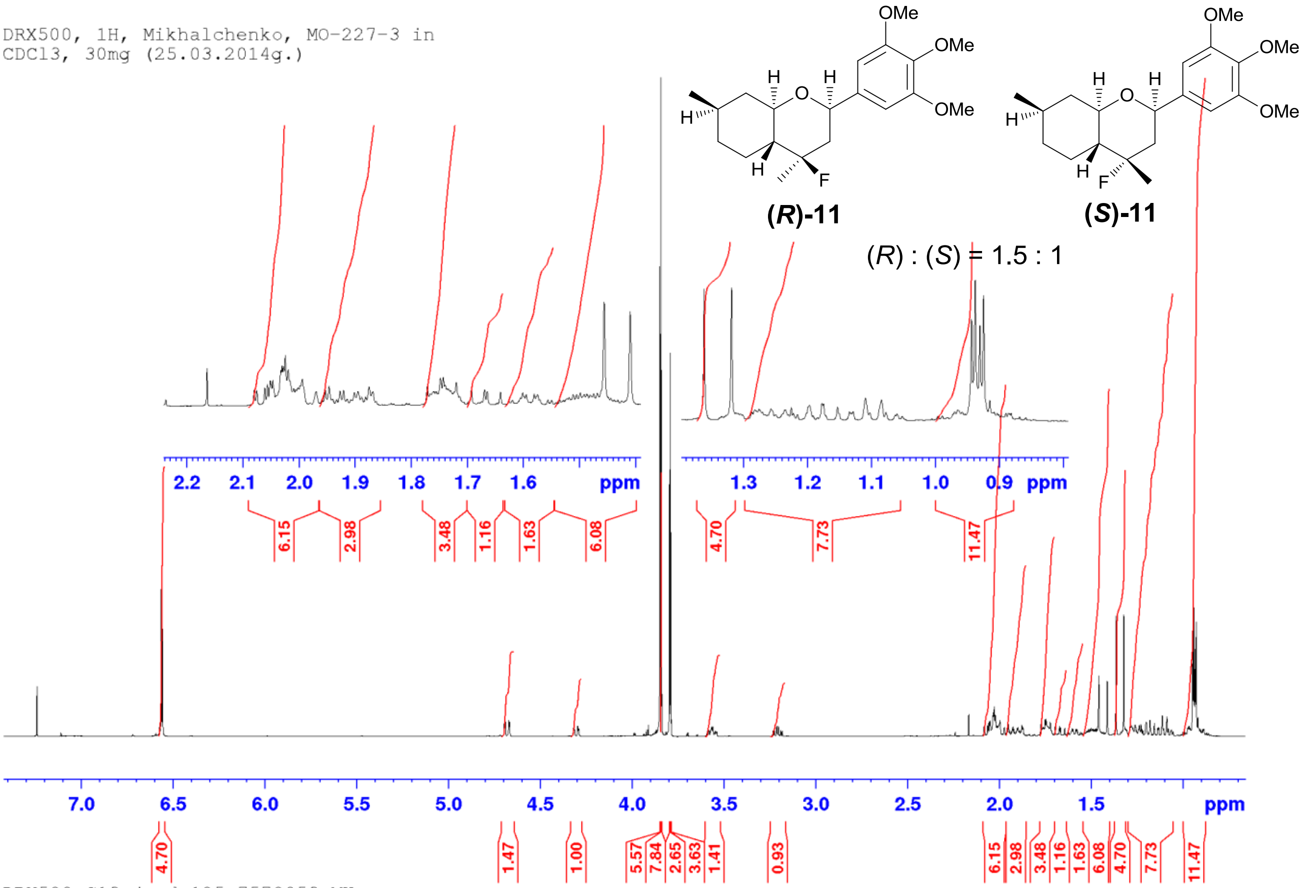


DRX500 C13 jmod 125.7578053 MHz,
Mikhalchenko, MO-227-3 in CDCl3, 30mg

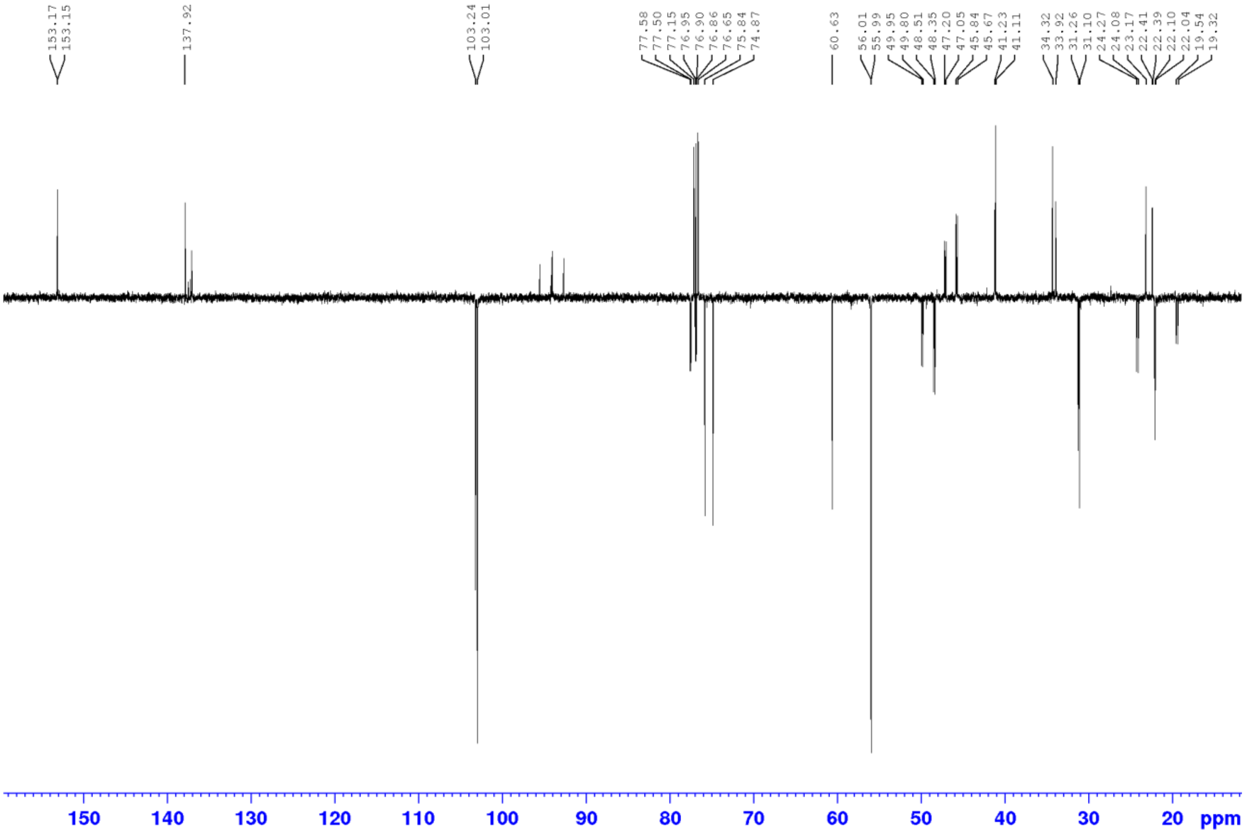

**References**

1. Il’ina, I. V.; Volcho, K. P.; Korchagina, D. V.; Barkhash, V. A.; Salakhutdinov, N. F. *Helv. Chim. Acta.* **2007**, *90*, 353-368;

2. Il'ina, I. V.; Volcho, K. P.; Mikhalchenko, O. S.; Korchagina, D. V.; Salakhutdinov N. F. *Helv. Chim. Acta,* **2011**, *94*, 502-513;

3. Mikhalchenko, O. S.; Korchagina, D. V.; Volcho, K. P.; Salakhutdinov, N. F. *Helv. Chim. Acta* **2014**, *97*, 1406-1421

4. Mikhalchenko, O.; Il’ina, I.; Pavlova, A.; Morozova, E.; Korchagina, D.; Tolstikova, T.; Pokushalov, E.; Volcho, K.; Salahutdinov, N. *Med. Chem. Res.* **2013**, *22*, 3026-3034;

5. Anikeev, V. I.; Sivcev, V. P.; Il’ina, I. V.; Korchagina, D. V.; Statsenko, O. B.; Volcho, K. P.; Salakhutdinov N. F. *Russ. J. Phys. Chem. A* **2013**, *87*, 382-387.
